# Supplementary material for: Protocol for a randomised feasibility study of Point-Of-care HIV viral load testing to Enhance Re-suppression in South Africa: the POwER study
Source: BMJ Open. 2021 Feb 16;11(2):e045373. doi: 10.1136/bmjopen-2020-045373 (PMC7888322; doi:10.1136/bmjopen-2020-045373)
Supplement: Supplementary data [file bmjopen-2020-045373supp001.pdf]

Study Title: POwER: Point Of care viral load testing to Enhance Re-suppression

Internal Reference Number / Short title: POwER

OxTREC Ref: 66-19

Date and Version No: Version 2.0 24th June 2020

**Chief Investigator:**	Dr Jienchi Dorward, Nuffield Department of Primary Care Health Sciences, University of Oxford
	Dr Nigel Garrett, Centre for the AIDS Programme of Research in South Africa, University of KwaZulu-Natal
**Investigators:**	Prof Chris Butler, Nuffield Department of Primary Care Health Sciences, University of Oxford
	Mrs Hope Ngobese, eThekwini Municipality Health Unit, eThekwini Municipality
	Dr Yukteshwar Sookrajh, eThekwini Municipality Health Unit, eThekwini Municipality
	Prof Gail Hayward, Nuffield Department of Primary Care Health Sciences, University of Oxford
	Dr Paul Drain, Department of Global Health, University of Washington
	Dr Richard Lessells, KwaZulu-Natal Research Innovation & Sequencing Platform, University of KwaZulu-Natal

**Sponsor:**	University of Oxford
**Funder:**	Internal Research England Global Challenges Research Fund, Wellcome Trust PhD Programme for Primary Care Clinicians, Africa Oxford Initiative.

Chief Investigator Signature:

Conflict of interest statement

We have no conflicts of interest to declare.

Confidentiality Statement

This document contains confidential information that must not be disclosed to anyone other than the authorised individuals from the University of Oxford, the Investigator Team and members of the Oxford Tropical Research Ethics Committee (OxTREC), unless authorised to do so.

Dorward J, *et al. BMJ Open* 2021; 11:e045373. doi: 10.1136/bmjopen-2020-045373

TABLE OF CONTENTS

1. SYNOPSIS 4
2. ABBREVIATIONS 5
3. BACKGROUND AND RATIONALE 6
4. OBJECTIVES AND OUTCOME MEASURES 7
5. STUDY DESIGN 8
6. STUDY SETTING 9
7. PARTICIPANT IDENTIFICATION AND RECRUITMENT 9
7.1. Study Participants 9
7.2. Inclusion Criteria 9
7.3. Exclusion Criteria 10
8. STUDY PROCEDURES 10
8.1. Recruitment 10
8.2. Informed Consent 10
8.3. Screening and Eligibility Assessment 10
8.4. Randomisation, blinding and code-breaking 11
8.5. Baseline Assessments 11
8.5.1. Baseline demographics and medical history 11
8.5.2. Laboratory testing 11
8.6. Follow-up Routine Clinic Visits 11
8.6.1. Viral load testing at follow-up routine clinic visits 11
8.6.2. Management of viral load results at follow-up routine clinic visits 12
8.6.3. Data captures at routine clinic visits 12
8.7. Study Exit Visit 12
8.8. Extended follow up of routine data 13
8.9. Sample Handling 13
8.9.1. Point-of-care testing 13
8.9.2. Standard laboratory viral load testing 13
8.9.3. Other standard laboratory tests 13
8.9.4. Storage samples and HIV drug resistance testing 13
8.9.5. Potential evaluation of newer point-of-care assays 14
8.10. Discontinuation/Withdrawal of Participants from Study 14
8.11. Definition of End of Study 14

9. SAFETY REPORTING 14
10. STATISTICS AND ANALYSIS 14
10.1. Description of Statistical Methods 14
10.2. Analysis of Outcome Measures 15
10.2.1. Primary outcome 15
10.2.2. Secondary outcomes 15
10.2.3. Tertiary Outcomes 15
10.3. The Number of Participants 15
11. DATA MANAGEMENT 16
11.1. Access to Data 16
11.2. Data Handling and Record Keeping 16
12. QUALITATIVE STUDY 16
12.1. Background 16
12.2. Aim 16
12.3. Study design 17
12.4. Participants 17
12.5. Procedures 17
12.5.1. Informed consent 17
12.5.2. Interviews and focus groups 17
12.5.3. Analysis 17
13. QUALITY CONTROL AND QUALITY ASSURANCE PROCEDURES 17
14. ETHICAL AND REGULATORY CONSIDERATIONS 18
14.1. Declaration of Helsinki 18
14.2. Guidelines for Good Clinical Practice 18
14.3. Approvals 18
14.4. Participant Confidentiality 18
14.5. Expenses and Benefits 19
14.6. Reporting 19
14.7. Other Ethical Considerations 19
15. FINANCE AND INSURANCE 19
15.1. Funding 19
15.2. Insurance 20
16. PUBLICATION POLICY 20
17. REFERENCES 21

18. APPENDIX A: STUDY FLOW CHART 23
19. APPENDIX B: SCHEDULE OF STUDY PROCEDURES 24
20. APPENDIX C: SOUTH AFRICAN GUIDELINES FOR MANAGEMENT OF VIRAEMIA 25
21. APPENDIX D: SOUTH AFRICAN GUIDELINES FOR CHOICE OF SECOND-LINE ART 26
22. APPENDIX E: AMENDMENT HISTORY 26

1. SYNOPSIS

**Study Title**	POwER: Point Of care viral load testing to Enhance Re-suppression	
**Internal ref. no.**	POwER	
**Study Design**	This will be a single-site, open-label, individually randomized feasibility study	
**Study Participants**	HIV-positive adults aged ≥18 years, receiving first-line antiretroviral therapy (ART), with latest viral load in the past 6 weeks ≥1000 copies/ml, at the Prince Cyril Zulu Clinic in Durban, South Africa.	
**Planned Sample Size**	Approximately 100	
**Planned Study Period**	Two years	
	**Objectives**	**Outcome Measures**
**Primary**	To estimate the effect of implementing point-of-care viral load testing versus standard laboratory viral load testing on the proportion of HIV positive participants with viraemia who subsequently achieve viral resuppression	The proportion of participants in each arm with viral load <50 copies/mL after 24 weeks
**Secondary**	To assess whether it is feasible to perform a randomised implementation trial of point-of-care viral load testing to manage viraemia in a routine South African clinic, by determining:	
	a) What proportion of people with HIV viraemia is feasible to enrol?	a) Proportion of patients with viraemia at the study clinic who are enrolled in the study
	b) What proportion of those enrolled is feasible to follow up?	b) Proportion of enrolled participants who attend the 24 week study exit visit
	c) What proportion of point-of-care viral load results are received and acted on in the same day?	c) The proportion of point-of-care viral load tests that are communicated to participants on the same day, and the proportion resulting in same day enhanced adherence counselling and same day switch to second-line ART

**Tertiary Objectives**	To estimate the effect of point-of-care viral load testing versus standard laboratory viral load testing on:	
	a) Time to detection of viral failure (consecutive viral loads ≥1000 copies/mL) in each arm	a) Days from enrolment to availability of viral load result ≥1000 copies/mL
	b) Time to switch to second-line ART in each arm	b) Days from enrolment to appropriate switching to second-line ART, amongst participants with viral failure
	c) Dependent on dolutegravir rollout during the study period: Time to appropriate switching to dolutegravir in each arm	c) Days from enrolment to appropriate switching to dolutegravir-based ART according to South African guidelines
	d) HIV drug resistance in each arm	d) The proportion of participants in each arm with HIV drug resistance at 24 weeks
**Qualitative evaluation**	What changes in clinic systems are required and what are the views and experiences of staff in implementing point-of-care viral load testing to manage viraemia?	Staff perspectives regarding implementation of point-of-care viral load testing to manage viraemia

2. ABBREVIATIONS

BREC	Biomedical Research Ethics Committee
CAPRISA	Centre for the AIDS Programme of Research in South Africa
CI	Chief Investigator
CRF	Case Report Form
eGFR	Estimated glomerular filtration rate
GCP	Good Clinical Practice
HIV	Human Immunodeficiency Virus
ICF	Informed Consent Form
LMIC	Low- and middle-income country
MDRD	Modification of Diet in Renal Disease
NHLS	National Health Laboratory Service
OxTREC	Oxford Tropical Research Ethics Committee
PI	Principal Investigator
PIS	Participant Information Sheet
SOP	Standard Operating Procedure

WHO	World Health Organization

3. BACKGROUND AND RATIONALE

Initiating antiretroviral therapy (ART) to achieve viral load suppression amongst all people with HIV is crucial to achieve the UNAIDS target of ending AIDS by 2030. However, globally, 20% of people with HIV on ART have viraemia ≥1000 copies/mL, due to poor adherence and/or HIV drug resistance.[1] For these people, current World Health Organisation (WHO) guidelines recommend enhanced adherence counselling, with a repeat viral load within three months. If viraemia persists, HIV drug resistance is assumed, and second-line ART is recommended.[2,3] However, in low- and middle-income countries (LMICs), the majority of ART is provided in primary care, where there is limited laboratory viral load capacity, weak clinic systems to act on viral load results and a paucity of evidence based interventions to improve adherence.[4–9] Poor adherence can be due to complex social issues (e.g. sexual violence, alcohol use, migrant labour and long distances to clinics).[10] Therefore, multiple visits for blood draw, result review and repeated enhanced adherence counselling confers an additional burden for this vulnerable population. Laboratory viral load results are often not reviewed until the next routine clinic visit after several months, by which time adherence problems may have worsened.[4–9] Overall, these health system and individual factors result in ongoing viraemia, with associated increases in morbidity, mortality, onward HIV transmission and development and spread of HIV drug resistance.[4]

Point-of-care viral load testing could overcome some of these challenges by allowing a) immediate enhanced adherence counselling to address contemporary adherence b) same-day switching to second-line ART, and c) more efficient, person-centred care by reducing the burden of clinic visits for review of blood results; thereby leading to faster viral load re-suppression. WHO has approved the Xpert® HIV-1 VL (Cepheid, Sunnyvale, USA)[11] and the m-PIMA HIV-1/2 VL (Abbott, Chicago, USA)[12] as quantitative point-of-care HIV viral load assays for use in LMICs. The Xpert HIV-1 VL has been validated in multiple settings, including the proposed clinical site,[13] with good correlation and sensitivity of 96.5% and specificity of 96.6% to detect viraemia at a threshold of 1000 copies/mL.[14] This assay uses the GeneXpert platform which is widely available in LMICs for tuberculosis diagnostics. We performed the first pilot randomized controlled trial[15] of the Xpert HIV-1 VL, and demonstrated that in a research setting, point-of-care viral load testing helped to rapidly triage people with viral load suppression into more efficient care pathways. We found a 14% increase in retention in care and viral load suppression in the point-of-care testing arm compared to standard laboratory viral load testing.[15] However, this study **excluded clinically unstable patients** (only 5% had viraemia at enrolment). Therefore, we have inadequate data on the effect of point-of-care viral load testing amongst people with viraemia who are a vulnerable, priority population and could be difficult to enrol and follow up in trials. Furthermore, implementing point-of-care viral load testing requires multiple steps including assay maintenance, quality assurance and reorganisation of clinic flow, to ensure the result is used to provide immediate enhanced adherence counselling or switching of ART regimen in the same clinic visit, by the appropriate healthcare worker. In STREAM, research staff provided point-of-care viral load testing and clinical care, meaning that we do not know if implementation is feasible in routine primary care services. Therefore, we aim to test whether implementing point-of-care viral load testing to improve the management of viraemia is feasible, and whether the effect of point-of-care viral load testing on management of viraemia can be tested in a trial.

Lastly, many LMICs are now replacing current non-nucleoside reverse transcriptase inhibitors (NNRTIs)

with the new drug dolutegravir for first-line ART.[16] Dolutegravir is an integrase strand transfer inhibitor that, in high income settings, has been found to have better tolerability, efficacy, and durability than current NNRTI regimens. In South Africa, the rollout of dolutegravir is expected in late 2019/early 2020. Patients currently on NNRTI based first-line ART will require viral load testing to guide switching to dolutegravir-based first-line or second-line ART.[17] Our implementation study will likely coincide with the dolutegravir rollout, and presents an opportunity to assess whether point-of-care viral load testing can assist with the viral load informed transition to this newer regimen.

4. OBJECTIVES AND OUTCOME MEASURES

**Objectives**	**Outcome Measures**	**Timepoint(s) of evaluation of this outcome measure (if applicable)**
**Primary Objective**<br>To estimate the effect of implementing point-of-care viral load testing versus standard laboratory viral load testing on the proportion of HIV positive participants with viraemia who subsequently achieve viral resuppression	The proportion of participants in each arm with viral load <50 copies/mL measured on laboratory reference assay	24 weeks after enrolment
**Secondary Objectives**<br>To assess whether it is feasible to perform a randomised implementation trial of point-of-care viral load testing to manage viraemia in a routine South African clinic, by determining:		
a) What proportion of people with HIV viraemia is feasible to enrol?	a) Proportion of patients with viraemia at the study clinic who are successfully enrolled in the study	a) During study enrolment period
b) What proportion of those enrolled is feasible to follow up?	b) Proportion of enrolled participants who attend the study exit visit	b) 24 weeks after enrolment
c) What proportion of point-of-care viral load results are received and acted on in the same day?	c1) The proportion of point-of-care viral load tests that are communicated to participants on the same day<br>AND<br>c2) The proportion of point-of-care viral load tests ≥1000 copies/mL that result in same day enhanced adherence counselling<br>AND	c) 12 weeks after enrolment when point-of-care testing is performed

	c3) The proportion of point-of-care viral load tests ≥1000 copies/mL that result in same day switch to second-line ART	
**Tertiary Objectives**<br>To estimate the effect of point-of-care viral load testing versus standard laboratory viral load testing on:		
a) Time to detection of viral failure (consecutive viral loads ≥1000 copies/mL) in each arm	a) Days from enrolment to availability of viral load result ≥1000 copies/mL	a) By 24 weeks after enrolment
b) Time to switch to second-line ART in each arm	b) Days from enrolment to appropriate switching to second-line ART, amongst participants with viral failure	b) By 24 weeks after enrolment
c) Dependent on dolutegravir rollout during the study period: Appropriate switching to dolutegravir in each arm	c) The proportion of participants in each arm appropriately switched to dolutegravir-based ART according to South African guidelines	c) 24 weeks after enrolment
d) HIV drug resistance in each arm	d) The proportion of participants in each arm with HIV drug resistance	d) 24 weeks after enrolment
**Qualitative Study Objectives:**<br>What changes in clinic systems are required and what are the views and experiences of staff in implementing point-of-care viral load testing to manage viraemia?	Staff perspectives regarding implementation of point-of-care viral load testing in a routine clinic	During enrolment when point-of-care testing is being implemented, and after study conclusion

5. STUDY DESIGN

This will be a single-site, open-label, individually randomized feasibility study amongst people with recent HIV viraemia, comparing point-of-care viral load testing with standard laboratory viral load testing. We will also assess implementation of point-of-care viral load testing using process evaluation data, interviews and focus groups.

Eligible people will be HIV-positive, aged ≥18 years, receiving first-line ART, with latest viral load in the past 6 weeks ≥1000 copies/ml, and who have not received enhanced adherence counselling. Participants who provide written consent will be screened, enrolled and randomized in a 1:1 ratio to point-of-care viral load testing versus standard laboratory viral load testing (Appendix A). After randomization and during follow up, participants in both arms will continue to have routine clinic visits with clinical care, enhanced adherence counselling and blood draws from clinic staff as per South African and local guidelines, which recommend monthly visits until viral suppression is achieved. Repeat viral load testing will be performed at the clinician's discretion in accordance with South African guidelines, which recommend a repeat viral load after three months of enhanced adherence counselling. The Xpert® HIV-1 VL (or similar WHO approved assay e.g. m-PIMA HIV-1/2 VL) will be used in the point-of-care arm, and standard laboratory viral load testing in the standard of care arm. Clinic staff will be encouraged to use point-of-care viral load

Dorward J, et al. BMJ Open 2021; 11:e045373. doi: 10.1136/bmjopen-2020-045373

results to provide immediate enhanced adherence counselling in the point-of-care arm, while in the SOC arm laboratory viral load results will be available to guide enhanced adherence counselling at the next clinic visit, which can be scheduled at the healthcare worker and client's discretion (typically after 28 days to coincide with next ART collection). Those with viral load ≥1000 copies/ml will meet criteria for viral failure (two consecutive viral loads ≥1000 copies/mL over 3 months apart), and will be managed according to South African viral failure guidelines, which recommend consideration of switching to a second-line ART regimen.[17] Those with viral loads <1000 copies/mL will continue their current first-line ART .[17] At the 24 week study exit visit a study outcome viral load will be measured using a laboratory reference assay (e.g. Alinity m HIV-1 or similar validated assay) for comparison between the two arms.

At enrolment, participants demographics, socioeconomic status and medical history will be recorded by research staff using standard questionnaires and review of clinical notes. During follow up, research staff will review participants clinic and laboratory notes to record time timing of receipt of viral load results, enhanced adherence counselling and switches to second-line ART. Data will be captured using electronic case report forms (CRFs) in REDCap.

During implementation of point-of-care viral load testing and after the study ends, a research assistant will perform in depth interviews and focus group discussions with clinic staff to explore implementation of point-of-care testing for management of viraemia (see section 12). Interviews and focus groups will be audio recorded, transcribed and analysed using Framework analysis.

6. STUDY SETTING

The study will take place at the Prince Cyril Zulu Clinic with support from the adjacent CAPRISA eThekwini Clinical Research Site. The Prince Cyril Zulu Clinic is a large public clinic situated next to the main transport hub in central Durban and provides HIV, tuberculosis sexual health and primary care services to a large, diverse, urban population. Prince Cyril Zulu Clinic sees approximately 40 people per month with viraemia ≥1000 copies/mL on first-line ART.

7. PARTICIPANT IDENTIFICATION AND RECRUITMENT

7.1. Study Participants

HIV-positive adults aged ≥18 years, receiving first-line ART, with latest viral load in the past 6 weeks ≥1000 copies/ml, and not having received enhanced adherence counselling.

7.2. Inclusion Criteria

The participant may enter the study if ALL of the following apply:

- Participant is willing and able to give informed consent for participation in the study.
- HIV positive adult, Male or Female, aged ≥18 years.
- Receiving first-line ART
- Latest viral load in the past 6 weeks ≥1000 copies/mL

Dorward J, *et al. BMJ Open* 2021; 11:e045373. doi: 10.1136/bmjopen-2020-045373

7.3. Exclusion Criteria

The participant may not enter the study if ANY of the following apply:

- Has already received enhanced adherence counselling at a previous visit
- Requires hospital admission
- Pregnant

Pregnant women will not be enrolled in the study as they are routinely referred out of PCZ CDC for antenatal care. Participants who become pregnant during the study will be referred for antenatal care but will continue to be offered follow up in the study.

8. STUDY PROCEDURES

8.1. Recruitment

HIV-positive patients on first-line ART and with recent viraemia will be identified by clinic staff using National Health Laboratory Service (NHLS) 'High Viral Load Reports', electronic clinic information systems and clinic notes, and offered referral to the study team.

8.2. Informed Consent

A research assistant or research nurse/designee will approach patients at the clinic who have been identified as having a high viral load. The research assistant or research nurse/designee will describe the study, address any questions and ask for voluntary participation. If the participant is willing, they will be invited to a private room in the clinic where the research assistant or designee will present written and verbal versions of the Participant Information and Informed Consent detailing no less than: the exact nature of the study; what it will involve for the participant; the implications and constraints of the protocol; the known side effects and any risks involved in taking part. It will be clearly stated that the participant is free to withdraw from the study at any time for any reason without prejudice to future care, and with no obligation to give the reason for withdrawal.

The participant will be allowed as much time as they wish to consider the information, and the opportunity to question the Investigator or other independent parties to decide whether they will participate in the study. Written Informed Consent will then be obtained by means of participant dated signature and dated signature of the person who presented and obtained the Informed Consent. The person who obtained the consent must be suitably qualified and experienced, and have been authorised to do so by the Chief Investigator. A copy of the signed Informed Consent will be offered to the participant. The original signed form will be retained at the study site.

The participant must personally sign and date the latest approved version of the Informed Consent form before any study specific procedures are performed.

8.3. Screening and Eligibility Assessment

After informed consent has been obtained, a research nurse will review the study inclusion/exclusion criteria with the patient. Contact details, demographics, a medical history and vital signs will be recorded. The patients clinical and laboratory record will be reviewed. Pregnancy testing will be performed for all

Dorward J, *et al. BMJ Open* 2021; 11:e045373. doi: 10.1136/bmjopen-2020-045373

women aged 18-55 who have not yet had a pregnancy test on that clinic visit. Patients deemed eligible for the study will be enrolled within 7 days of screening.

8.4. Randomisation, blinding and code-breaking

A CAPRISA statistician will generate the allocation sequence using random numbers generated in a statistical software program such as STATA 14.0 (Statacorp, Texas, USA) or SAS 9.4 (SAS Institute Inc., Cary, USA). Randomisation will be stratified by first-line ART regimen at enrolment (efavirenz-based or dolutegravir-based). The allocation sequence will contain variable block sizes, with participants randomised in a 1:1 ratio to the intervention or standard of care arm. The allocation sequence will be programmed into the REDCap (Vanderbilt University, Nashville, USA) enrolment electronic case report form (eCRF). All study staff apart from the statistician and data manager will be blinded to the allocation sequence. At enrolment, the research assistant or research nurse will complete the enrolment eCRF, which will automatically assign the intervention allocation and generate a unique participant identification number (PID). As this is an open label study, study staff, clinic staff and participants will not be blinded to intervention allocation.

8.5. Baseline Assessments

8.5.1. Baseline demographics and medical history

At enrolment, all participants will have a baseline sociodemographic questionnaire, clinical history including ART history, and examination. South African guidelines for clinical assessment and management of patients with a high viral load will be followed,[17] including enhanced adherence counselling performed by clinic staff using the pre-enrolment viral load result. In both arms, enhanced adherence counselling will follow South African guidelines, which include ART education, evaluation of social support, mental health screening and use of treatment supporters where appropriate.[18]

8.5.2. Laboratory testing

For all participants, a phlebotomist or nurse will draw venous blood for tests recommended by South African guidelines for investigation of viraemia, which currently include CD4 count testing, hepatitis B surface antigen, creatinine and haemoglobin. Venous blood for storage and retrospective HIV drug resistance testing will also be drawn.

8.6. Follow-up Routine Clinic Visits

Participants in both arms will have routine clinic visits scheduled at the healthcare worker and participants discretion, but normally every 28 days to coincide with ART collection. At these visits, clinic staff will provide ongoing enhanced adherence guidelines, clinical support and ART when necessary, as per the standard of care in South African National Guidelines. Clinicians will be able to request viral load testing as per South African guidelines, which recommend a repeat viral load 3 months after the first high result ≥1000 copies/mL.

8.6.1. Viral load testing at follow-up routine clinic visits

For participants randomised to the point-of-care arm, a phlebotomist or nurse will draw venous blood for point-of-care viral load testing in the clinic. Results should be available in approximately two hours. Staff will be encouraged to provide results in the same clinic visit to inform ongoing management. If results are

not available in the same visit, or if the client cannot wait, they will be available at the next clinic visit scheduled at staff and patient's discretion.

In the standard of care arm, a phlebotomist or nurse will draw venous blood for laboratory viral load testing which will be sent to the standard of care National Health Laboratory Service (NHLS) as per routine clinic procedures. Results are normally available after 7 days and will be provided to the participant at the next clinic visit, arranged at the participants and healthcare worker's discretion (typically after 7 -28 days for results, depending on the participants availability, ART supply and clinic schedules).

For participants in both arms, a venous blood sample will also be drawn for storage alongside the repeat viral load result.

8.6.2. Management of viral load results at follow-up routine clinic visits

Viral load results will be managed in accordance with South African Department of Health guidelines, which include guidance for management of both NNRTI and dolutegravir-based regimens (Appendix C).[17] Participants with a viral load ≥1000 copies/ml may meet criteria for viral failure (two consecutive viral loads ≥1000 copies/mL over 3 months apart) and will be considered for switch to second-line ART. Switch to second-line ART and the choice of regimen will be at the clinician's discretion and will depend on the participant's first-line ART, previous ART history, participant's preferences, perceived adherence and current South African national and local clinical guidelines (Appendices C and D).[17] The risks and benefits of potential new regimens will be discussed with the participant as per usual practice. Participants with a second viral load <1000 copies/mL will continue first-line ART, with enhanced adherence counselling for those with low level viraemia between 50-999 copies/mL (Table 1). If South Africa introduces dolutegravir during the study period, then study participants on NNRTI-based first-line ART with a suppressed viral load <50 copies/mL will be eligible for dolutegravir-based first-line ART.

Table 1: Management of viral load results during follow-up

HIV viral load result	Definition	Action
≥1000 copies/mL	Potential viral failure	Consider switch to second-line ART
50-999 copies/mL	Low level viraemia	Continue current first-line ART and enhanced adherence counselling
<50 copies/mL	Viral suppression	Continue first-line ART, if on NNRTI then may be eligible for dolutegravir based regimen.

8.6.3. Data captures at routine clinic visits

Research staff will monitor participants clinical records to capture visit dates, whether enhanced adherence counselling was performed, medication prescriptions including ART, and laboratory results.

8.7. Study Exit Visit

Clinical follow up will end at 24 weeks when a study outcome viral load will be measured using a laboratory reference assay (e.g. Alinity m HIV-1 or similar validated assay) for comparison between the two arms. A venous blood sample for storage and retrospective HIV drug resistance testing will also be taken. At 24 weeks, any participant who has not attended the study exit visit will be called by the research team and asked to attend. If by 30 weeks it has not been possible to contact the participant, they will be exited from the study.

8.8. Extended follow up of routine data

For participants who agree, research staff may access routine medical records, prescription data and laboratory results for up to five years after the study exit visit, in order to determine longer term retention in care, ART adherence and viral load results. For participants who are lost to follow up, and who provided consent at enrolment, the participants vital status may be checked on the South African National Population Register.

8.9. Sample Handling

8.9.1. Point-of-care testing

Point-of-care VL testing will be performed by a nurse, phlebotomist or technician in the clinic, who will have received comprehensive training on all aspects of performing point-of-care viral load testing and been assessed as competent by the CAPRISA laboratory site manager. All point-of-care viral load testing procedures will be performed in a separate, specially prepared room in the clinic, using a small centrifuge and the Xpert HIV-1 VL assay on the GeneXpert platform, or a similar WHO approved point-of-care assay (e.g. the m-PIMA HIV-1/2 VL). Testing will be performed following manufacturer instructions, using approximately 4 mLs of venous blood, drawn using aseptic technique. The sample will be centrifuged to separate approximately 1 mL of plasma which will be pipetted into the assay cartridge. The cartridge will be placed in the fully automated assay platform, with results available after approximately 90 minutes. In the case of an invalid result, a repeat blood sample may need to be drawn for point-of-care testing. If repeat point-of-care testing is not possible (e.g. machine failure) the repeat sample will be sent for standard laboratory testing. Error codes will be checked regularly and reported to the CAPRISA laboratory staff and manufacturer to identify and correct sample processing or machine errors.

8.9.2. Standard laboratory viral load testing

Laboratory viral load testing will be performed by a nurse or phlebotomist in the clinic according to standard clinic procedures. In brief, approximately 4 mLs of venous will be drawn using aseptic technique into an EDTA tube and transported on the same day to the National Health Laboratory Service for testing using their routine laboratory assays. Currently, the National Health Service performs viral load tests on the Alinity m HIV-1 assay (Abbott, Chicago, USA) at the Addington Hospital Laboratory, or the Inkosi Albert Luthuli Hospital Laboratory, depending on machine availability and capacity.

8.9.3. Other standard laboratory tests

At enrolment, approximately 20 mLs of venous blood will be drawn for CD4 count testing (4 mLs EDTA), hepatitis B surface antigen (5 mLs SST), creatinine (5 mLs SST) and haemoglobin (4 mLs EDTA). Samples will be transported on the same day to the National Health Laboratory Service for testing using their routine laboratory assays.

8.9.4. Storage samples and HIV drug resistance testing

Participants who provide additional consent will also have 10 mLs of venous blood for storage and retrospective ART drug level and HIV drug resistance testing, taken at enrolment, during follow up alongside the repeat viral load test, and at study exit. At enrolment, 5 mLs of urine will also be taken for storage and ART drug level testing. Samples will be stored in the CAPRISA biorepository according to Good Clinical Laboratory Practice. At the end of the study, HIV drug resistance testing will be performed on viraemic samples. The threshold of viraemia for HIV drug resistance testing will be determined by the likelihood of amplification on the assay being used. We will use standard Sanger sequencing or next-

generation sequencing with the Illumina MiSeq platform. Remaining blood will be stored for ten years after the end of the study and will be made available to the study team or other investigators who have obtained ethical approval for additional projects. After ten years, remaining stored samples will be disposed of.

8.9.5. Potential evaluation of newer point-of-care assays

If new point-of-care assays that may improve HIV care become available (such as viral load, HIV drug resistance, and ART drug level assays), we may use samples from this study to compare results from the new assay to the laboratory reference assay. At blood draw visits we may ask participants to provide up to 8 mLs venous blood and/or a finger-prick blood sample for evaluation of these new point-of-care assays. The new assay results would be for validation purposes only and would not be used to guide clinical management.

8.10. Discontinuation/Withdrawal of Participants from Study

Each participant has the right to withdraw from the study at any time. In addition, the Investigator may discontinue a participant from the study at any time if the Investigator considers it necessary for any reason including:

- Ineligibility (either arising during the study or retrospectively having been overlooked at screening)
- Significant protocol deviation
- Significant non-compliance with treatment regimen or study requirements
- Withdrawal of Consent

Participants who withdraw from the study will not be replaced. Data for participants who withdraw will be included in intention to treat analysis. The reason for withdrawal will be recorded in the Case Report Form.

8.11. Definition of End of Study

The end of study is the date 30 weeks after enrolment of the last participant.

9. SAFETY REPORTING

This study does not involve an investigational medicinal product or intervention that affects physiology, and therefore will not require serious adverse event reporting. However, for completeness we will report all cases of mortality in the study to the University of KwaZulu-Natal's Biomedical Research Ethics Committee (BREC).

10. STATISTICS AND ANALYSIS

10.1. Description of Statistical Methods

At the conclusion of the study, we will assess the proportion of participants achieving study outcomes in each arm, with 95% confidence intervals calculated using the Wilson method. We will use Fisher's exact test to compare the proportions achieving study outcome measures in each arm. However, as this is a

pilot study, we will not be powered to test the hypotheses that there is a difference in proportions between the two arms for most of our outcome measures.

10.2. Analysis of Outcome Measures

10.2.1. Primary outcome

We will calculate the proportion of participants in each arm achieving the binary primary outcome of viral suppression <50 copies/mL at 24 weeks (Table 2). Participants who are lost to follow up with no viral load result will be included the analysis as not having achieved the primary outcome, meaning all participants enrolled in each arm will be included in the analysis.

10.2.2. Secondary outcomes

We will calculate the proportion of patients with viraemia at the Prince Cyril Zulu clinic who are enrolled in the study. The denominator will be the number of patients with viraemia during the study enrolment period, which will be calculated by clinic staff using routinely generated NHLS high viral load reports. These reports are routinely used for monitoring and evaluation of clinic performance and include the number of first high viral loads > 1000 copies/mL taken from Prince Cyril Zulu each week. To assess study follow up, we will calculate the proportion of participants who attend the study exit visit at 24 weeks after enrolment. To assess implementation of point-of-care viral load testing, we will calculate the proportion of point-of-care viral load tests which are provided to participants on the same day, the proportion of viral loads ≥1000 copies/mL which resulted in same day enhanced adherence counselling, and the proportion of viral loads ≥1000 copies/mL resulting in appropriate same-day switch to second-line ART.

10.2.3. Tertiary Outcomes

We will assess the median number of days (and interquartile range) from enrolment to detection of viral failure, and from enrolment to appropriate switch to second-line ART, amongst participants with viral failure. We will also calculate the proportion of participants in each arm who are appropriately switched to a dolutegravir-based first or second line ART regimen by 24 weeks, and also the proportion of those with HIV drug resistance detected at 24 weeks after enrolment.

10.3. The Number of Participants

PCZC sees approximately 40 patients per month with viraemia on first-line ART. We conservatively estimate (based on our previous study) that we could recruit approximately 40% of these in 6 months, a total of 100 participants. However, as this is a feasibility study, the exact number that can be enrolled cannot be determined. The COVID-19 pandemic adds further uncertainty, as interruptions to care may decrease potential enrolment, or lead to higher numbers of people with viraemia during the study period, which could also increase enrolment. Therefore, while we anticipate 100 participants, the final number

Table 2: Study & process evaluation outcomes with precision estimates

Outcome	Estimated n/N, %	95% CI (Wilson)
Percentage with viral load <50 copies/ml at 24 weeks in POC arm	35/50, 70.0	56.2-80.9
Percentage with viral load <50 copies/ml at 24 weeks in SOC arm	25/50, 50.0	36.6-63.4
Percentage of eligible patients successfully enrolled in study	100/240*, 41.7	35.6-48.0
Percentage of those enrolled who are retained at 24 weeks	90/100, 90.0	82.6-94.5
Percentage in POC arm with same-day viral load testing	40/50, 79.5	70.0-88.8

*Assuming 240 eligible patients during enrolment period. POC = point-of-care, SOC = standard-of-care

may be between approximately 80-180 participants, and will be determined by the time and resources available. We will use NHLS, PCZC and study data to assess outcomes, with estimated precision, in Table 2. Although not the aim of this feasibility study, assuming 50% of SOC participants achieve the main outcome of viral load suppression <50 copies/ml at 24 weeks, a sample size of 50 participants per arm would provide 54% power to detect a +20% difference in the point-of-care testing arm, using a 2-sided alpha of 0.05. A difference of +10% would have a Newcombe-Wilson 95% confidence interval of -9 to +28%. A sample size of 90 participants per arm would provide 80% power to detect a +20% difference in the point-of-care testing arm, using a 2-sided alpha of 0.05. As all enrolled participants will be included in the final analysis, we have not adjusted for loss to follow up or potential withdrawals.

11. DATA MANAGEMENT

11.1. Access to Data

Direct access will be granted to authorised representatives from the University of Oxford and CAPRISA for monitoring and/or audit of the study to ensure compliance with regulations.

11.2. Data Handling and Record Keeping

The research team will capture study data using standardised electronic CRFs in REDCap. All data entry will undergo three stages of quality control including pre-programmed data validity checks in electronic CRFs, immediate source document review, and weekly quality reports generated by REDCap. The participants will be identified by a unique study specific number and/or code in any database. The name and any other identifying detail will NOT be included in any study data electronic file.

12. QUALITATIVE STUDY

12.1. Background

Point-of-care diagnostics have been used widely in healthcare systems in many low and middle income countries.[19] In HIV programmes, some tests such as rapid, lateral flow assays for diagnosis of HIV, have been evaluated, endorsed and incorporated into guidelines by the World Health Organization and successfully adopted in many settings.[20] However, other assays, such as more complex molecular polymerase chain reaction technologies for tuberculosis, have remained as laboratory tests despite being marketed as point-of-care assays.[19] A large systematic review of barriers to point-of-care HIV diagnostic implementation in low and middle income countries found that in 132 studies, integration of the point-of-care test into clinical work flows was the most commonly identified challenge to test utilisation.[21] However, there are few published implementation projects that have used a theory-based approach to assess implementation of point-of-care tests in healthcare systems, particularly in LMICs. One theory that has been widely used to assess implementation of new technologies in healthcare is Normalisation Process Theory. Normalisation Process Theory aims to identify what is needed to ‘normalise’ use of a technology in a healthcare system.

12.2. Aim

We aim to assess staff perspectives on implementation of point-of-care viral load testing for management of viraemia in a routine South African clinic.

12.3. Study design

During trial enrolment when implementation of point-of-care viral load testing is taking place, a trained research assistant with experience of qualitative research will perform the first round of semi-structured interviews with approximately 8-10 staff, and a focus group with approximately 8-10 staff. After completion of enrolment, we will perform a second round of interviews and focus group, to assess perspectives on implementation of point-of-care viral load testing and management of viraemia.

12.4. Participants

Approximately 10-15 staff involved in viral load testing and management of viraemia will be approached to participate in this qualitative study. Staff will include counsellors, phlebotomists, nurses, pharmacy staff, laboratory staff, doctors and health service managers. Focus groups and in-depth interviews may consist of the same or different staff members. However, we will endeavour to include the same staff members in the first and second round of interviews and focus groups.

12.5. Procedures

12.5.1. Informed consent

A trained research assistant will approach staff in the clinic, explain the nature of the project and offer participation. Should the staff member be interested, they will be taken to a private space and provided with written and verbal versions of the Qualitative Study Staff Participant Information Sheet and Informed Consent Form. The research assistant or other study team member will answer any questions that the staff member may have, and it will be clearly stated that staff are able to refuse to participate without adverse consequences for their working life. Once written informed consent has been provided, a copy will be offered to staff participant, and the original will remain with the research team.

12.5.2. Interviews and focus groups

Once the Informed Consent Form has been signed, the research assistant will use predetermined topic guides to conduct interviews and/or focus groups in a private room at the study clinic, or if necessary, at another suitable private venue of the participant’s choice. Interviews will last approximately 30-60 minutes, and focus groups 45-75 minutes. We will make an audio recording of interviews and focus group discussions. If a participant does not consent to audio recording, we will take a written recording of the interview or focus group.

12.5.3. Analysis

Audio and written recordings will be anonymised and translated from isiZulu (when necessary) and transcribed. Data will be analysed using thematic analysis as described by Attride-Stirling and reported accordingly.[22]

13. QUALITY CONTROL AND QUALITY ASSURANCE PROCEDURES

Study documentation including participant information sheets, informed consent forms, regulatory files, standard operating procedures, source documentation and electronic CRFs will be subject to internal quality audits by the CAPRISA Quality Assurance team, in accordance with CAPRISA standard operating procedures. CAPRISA routinely measure quality control and retention rates on all studies. The study will be conducted in accordance with relevant regulations and standard operating procedures.

14. ETHICAL AND REGULATORY CONSIDERATIONS

14.1. Declaration of Helsinki

The Investigator will ensure that this study is conducted in accordance with the principles of the Declaration of Helsinki.

14.2. Guidelines for Good Clinical Practice

The Investigator will ensure that this study is conducted in accordance with relevant regulations and with Good Clinical Practice.

14.3. Approvals

The protocol, participant information sheet and informed consent form, topic guides and any proposed advertising material will be submitted to OxTREC and BREC for written approval.

The Investigator will submit and, where necessary, obtain approval from the above parties for all amendments to the original approved documents.

14.4. Participant Confidentiality

The study staff will ensure that the participants' anonymity is maintained. The participants will be identified only by a participant ID number on all study documents and any electronic database. All documents will be stored in a secure cabinet in the locked research office, and will only accessible by study staff and authorised personnel. The electronic data will be stored in REDCap, which is a password protected data management service. All computers with access to the data will be password protected. The study will comply with the General Data Protection Regulation (GDPR), which requires that personal data must not be kept as identifiable data for longer than necessary for the purposes concerned.

For the qualitative evaluation of implementation processes, interviews and focus group discussions will take place in a private room. We will make a written record during the focus group or interview, or from the recorded audio, but we will not write down any information that might identify participants or others. For example, if the participant mentions names of colleagues, we will not write those names down. The recording and notes from interviews and focus group discussions will be stored in a locked cabinet and only the staff working on this study will be able to use them. We will destroy the recordings when the research is completed. Data will never be reported in a way that could potentially reveal a single participant's identity. If during interviews or focus groups a staff member reveals practice which is not in

line with professional standards and may cause harm to patients, we will discuss this with the staff member and the clinic manager.

14.5. Expenses and Benefits

Patient participants will be paid ZAR150 (approximately £8.00) after the enrolment visit and ZAR100 (approximately £5.50) after the 24 week visit for their participation in the research, in accordance with South African research guidelines.[23] Reasonable travel expenses for any visits additional to normal care will be reimbursed.

Staff participants will not be reimbursed as interviews or focus groups will take place during working hours, at a mutually convenient time that has been agreed with the clinic manager.

14.6. Reporting

The CI shall submit an Annual Progress Report to OxTREC and BREC on the anniversary of the date of approval of the study. In addition, the CI shall submit an End of Study Report to OxTREC and BREC within 12 months of completion of the study. If we extend follow up of routinely collected clinic and laboratory data, for up to five years after the last study exit visit, the study will remain under annual review by OxTREC and BREC while those data are being collected.

14.7. Other Ethical Considerations

All study participants will be HIV-positive and may face HIV-related stigma. Therefore, maintaining confidentiality is particularly important in this study (see section 14.4).

For staff interviews and focus groups, there is the possibility that they will feel unable to refuse participation, or that they will feel challenged at being interviewed about aspects of their work. While every effort will be made to maintain staff confidentiality, due to the small numbers of staff participating in the study it may be possible for superiors and colleagues to infer their identity indirectly. Therefore, the option to refuse participation without implications in their work will be made clear in the informed consent process. We will also warn staff about the risk of colleagues and superiors being able to infer their identity indirectly. Staff will not be asked questions which could threaten their work position or personal questions about their lives. Questions will focus work-related issues that are already likely to have been discussed in other settings, as the clinic team have implemented point-of-care viral load testing for management of viraemia.

15. FINANCE AND INSURANCE

15.1. Funding

This study is funded by a fellowship from Wellcome Trust PhD Programme for Primary Care Clinicians, the Internal Research England Global Challenges Research Fund (0007365) and the Africa Oxford Initiative.

15.2. Insurance

The University of Oxford has a specialist insurance policy in place which would operate in the event of any participant suffering harm as a result of their involvement in the research (Newline Underwriting Management Ltd, at Lloyd's of London).

16. PUBLICATION POLICY

Findings from this study will be presented at academic conferences and submitted to peer reviewed journals for Open Access publication in accordance with Wellcome Trust policies for Wellcome Trust funded research. Authorship and acknowledgements will follow the International Committee of Medical Journal Editors guidelines. Author accepted manuscripts will be posted on the investigators' institutional websites.

17. REFERENCES

1 Joint United Programme on HIV/AIDS (UNAIDS). Miles to Go: Global AIDS Update 2018. Geneva, 2018.

2 South African National Department of Health. National consolidated guidelines for the prevention of mother to child transmission of HIV (PMTCT) and the management of HIV in children, adolescents and adults. Pretoria, South Africa, 2015 http://www.sahivsoc.org/Files/ART Guidelines 15052015.pdf (accessed Feb 13, 2017).

3 World Health Organisation. Updated recommendations on first-line and second-line antiretroviral regimens and post-exposure prophylaxis and recommendations on early infant diagnosis of HIV: interim guidance. Geneva, 2018.

4 Murphy RA, Court R, Maartens G, Sunpath H. Second-line antiretroviral therapy in sub-Saharan Africa: It's time to mind the gaps. *AIDS Res Hum Retroviruses* 2017; **33**: 1181–4.

5 Labhardt ND, Ringera I, Lejone TI, *et al.* When patients fail UNAIDS' last 90 - the "failure cascade" beyond 90-90-90 in rural Lesotho, Southern Africa: a prospective cohort study. *J Int AIDS Soc* 2017; **20**: 1–10.

6 Lessells RJ. Virological monitoring – how it relates to adherence. In: 4th Southern African HIV Clinicians Society Conference. Johannesburg, 2018. http://www.sahivsoc2018.co.za/wp-content/uploads/2018/11/23B_Richard-Lessells-NV_VL-monitoring.pdf.

7 Jobanputra K, Parker LA, Azih C, *et al.* Impact and Programmatic Implications of Routine Viral Load Monitoring in Swaziland. *JAIDS J Acquir Immune Defic Syndr* 2014; **67**: 45–51.

8 Etoori D, Ciglenecki I, Ndlangamandla M, *et al.* Successes and challenges in optimizing the viral load cascade to improve antiretroviral therapy adherence and rationalize second-line switches in Swaziland. *J Int AIDS Soc* 2018; **21**: e25194.

9 Hermans LE, Tempelman HA, Carmona S, *et al.* High rates of viral resuppression on first-line ART after initial virological failure. In: Conference on Retroviruses and Opportunistic Infections. Boston, 2018. http://www.croiconference.org/sessions/high-rates-viral-resuppression-first-line-art-after-initial-virological-failure.

10 Shubber Z, Mills EJ, Nachega JB, *et al.* Patient-Reported Barriers to Adherence to Antiretroviral Therapy: A Systematic Review and Meta-Analysis. *PLOS Med* 2016; **13**: e1002183.

11 Dorward J, Drain PK, Garrett N. Point-of-care viral load testing and differentiated HIV care. *Lancet HIV* 2018; **5**: e8–9.

12 Bwana P, Ageng'o J, Danda J, Mbugua J, Handa A, Mwau M. Performance and Usability of mPIMATM HIV 1/2 Viral Load test in point of care settings in Kenya. *J Clin Virol* 2019; published online Oct. DOI:10.1016/j.jcv.2019.104202019.

13 Garrett NJ, Drain PK, Werner L, Samsunder N, Abdool Karim SS. Diagnostic Accuracy of the Point-of-Care Xpert HIV-1 Viral Load Assay in a South African HIV Clinic. *JAIDS J Acquir Immune Defic Syndr* 2016; **72**: e45–8.

14 Sacks JA, Fong Y, Gonzalez MP, *et al.* Performance of Cepheid Xpert HIV-1 viral load plasma assay to accurately detect treatment failure. *AIDS* 2019; **33**: 1881–9.

15 Drain PK, Dorward J, Violette L, *et al.* Point-of-care viral load testing improves HIV viral suppression and retention in care. In: Conference on Retroviruses and Opportunistic Infections.

Seattle, Washington, 2019.

16 Dorward J, Lessells R, Drain PK, *et al.* Dolutegravir for first-line antiretroviral therapy in low-income and middle-income countries: uncertainties and opportunities for implementation and research. *Lancet HIV* 2018; **5**: e400–4.

17 The South African National Department of Health. 2019 ART Clinical Guidelines for the management of HIV in Adults, Pregnancy, Adolescents, Children, Infants and Neonates. Pretoria, South Africa, 2019.

18 South African National Department of Health. Adherence Guidelines for HIV, TB and NCDs. Pretoria, 2016.

19 Jani I V., Peter TF. How Point-of-Care Testing Could Drive Innovation in Global Health. *N Engl J Med* 2013; **368**: 2319–24.

20 Drain PK, Rousseau C. Point-of-care diagnostics: Extending the laboratory network to reach the last mile. *Curr Opin HIV AIDS* 2017; **12**: 175–81.

21 Pai NP, Wilkinson S, Deli-Houssein R, *et al.* Barriers to Implementation of Rapid and Point-of-Care Tests for Human Immunodeficiency Virus Infection: Findings From a Systematic Review (1996-2014). *Point Care* 2015; **14**: 81–7.

22 Attride-Stirling J. Thematic networks: an analytic tool for qualitative research. *Qual Res* 2001; **1**: 385–405.

23 National Health Research Ethics Council (NHREC). Payment of trial participants in South Africa: Ethical considerations for Research Ethics Committees. South African Department of Health, 2012 http://www.nhrec.org.za/docs/Documents/Paymentconsiderations.pdf (accessed March 10, 2017).

18. APPENDIX A: STUDY FLOW CHART

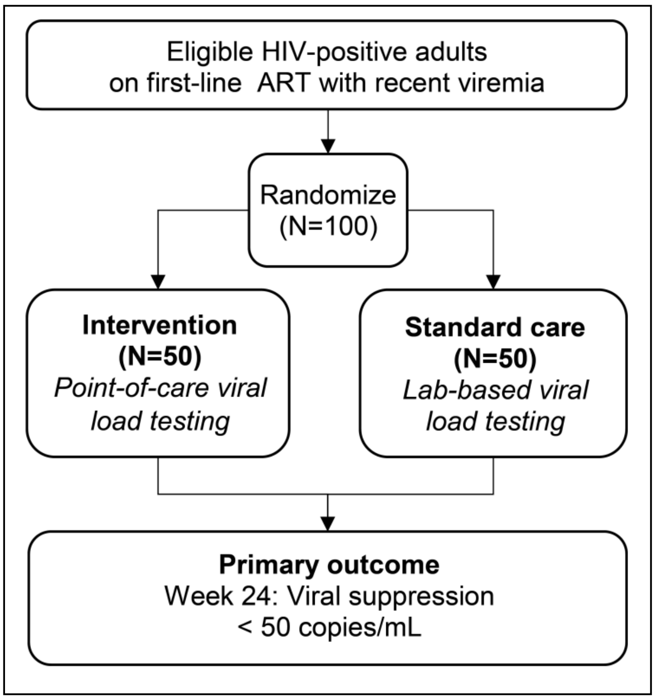

19. APPENDIX B: SCHEDULE OF STUDY PROCEDURES

	Enroll	Routine clinic visit follow-up[a]					Study exit
**Weeks in study**	**0**	**4**	**8**	**12**	**16**	**20**	**24**
Informed consent	X						
Locator information	X						
Demographics	X						
Medical history	X						
Vital signs	X	X	X	X	X	X	X
Physical examination	X						
Urine pregnancy test	X						
Eligibility screen	X						
Enhanced adherence counselling [b]	X	X	X	X	X†	X†	X†
Randomization	X						
Routine Department of Health testing [c]	X						
POC viral load (intervention arm)				X			
Laboratory viral load (standard of care arm)				X			
Reference viral load outcome measure							X
Evaluation of new POC assays[d]	X			X			X
Stored blood & HIV drug resistance testing[e]	X			X			X
Stored urine for ART drug level testing	X						

[a] Scheduled at participants and healthcare worker's discretion but typically every 28 days.
[b] Ongoing enhanced adherence counselling for participants with viral load ≥50 copies/mL.
[c] CD4 count testing (4 mLs EDTA), hepatitis B surface antigen (5 mLs SST), creatinine (5 mLs SST) and haemoglobin (4 mLs EDTA).
[d] Maximum of 8 mLs venous blood and/or a finger-prick capillary blood sample
[e§] Retrospective drug resistance testing for viraemic participants at enrolment, follow up or study exit (10 mLs EDTA)
ART = antiretroviral therapy, POC = point-of-care

20. APPENDIX C: SOUTH AFRICAN GUIDELINES FOR MANAGEMENT OF VIRAEMIA

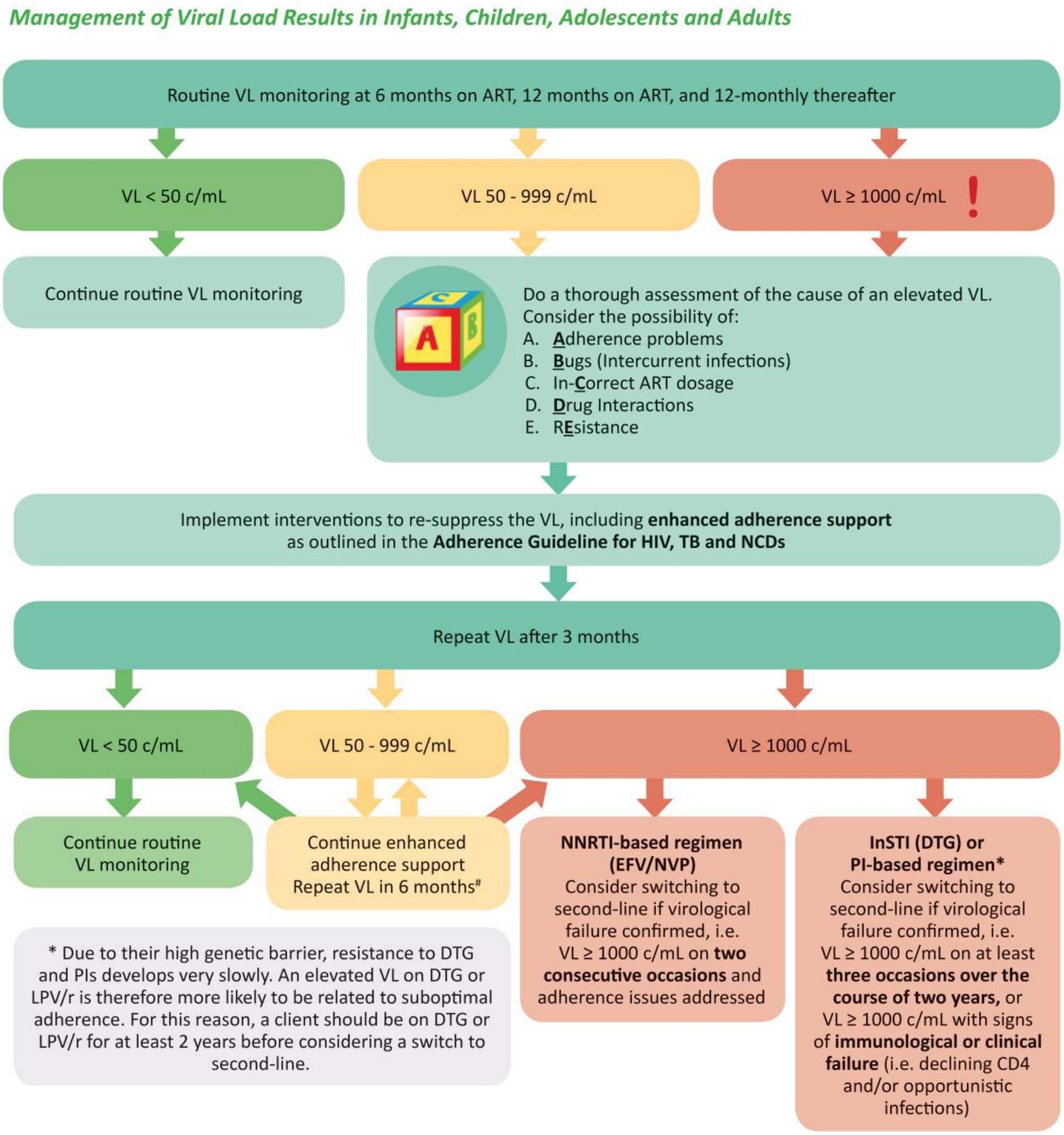

Dorward J, et al. BMJ Open 2021; 11:e045373. doi: 10.1136/bmjopen-2020-045373

21. APPENDIX D: SOUTH AFRICAN GUIDELINES FOR CHOICE OF SECOND-LINE ART

Second-line ART Regimens for Adults with Confirmed Virological Failure

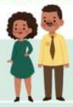

	First-Line Regimens			
	NNRTI-based Regimen		InSTI-based Regimen for > 2 years	
Regimen	TDF + 3TC/FTC + EFV/NVP		TDF + 3TC/FTC + DTG	
Resistance Testing	Resistance test not required		Resistance testing may be required under expert consultation [5]	
Resistance Test results	Not applicable		Not applicable	
HBV Co-infection Status [1]	HBV-negative	HBV-positive	HBV-negative	HBV-positive
New Regimen	AZT + 3TC/FTC + DTG[2]	TDF[1] + AZT + 3TC/FTC + DTG[2]	AZT + 3TC/FTC + LPV/r	TDF + 3TC/FTC + LPV/r [4]
	If DTG not suitable[2], AZT + 3TC/FTC + LPV/r	If DTG not suitable[2], TDF + 3TC + LPV/r[4]		

22. APPENDIX E: AMENDMENT HISTORY

Amendment No.	Protocol Version No.	Date issued	Author(s) of changes	Details of Changes made
1	1.1	10th November 2019	Jienchi Dorward	1. Addition of co-investigators Mrs H Ngobese and Dr Y Sookrajh<br>2. Addition of alternative, World Health Organization approved point-of-care viral load assays that could be used in the study<br>3. Addition of optional extended follow up of participants routinely collected clinic data and vital status in the South African National Population Register.<br>4. Change in the duration of blood storage from two to ten years<br>5. Addition of the potential evaluation of new point-of-care viral load assays<br>6. Minor clarifications regarding i) storage blood being drawn at week 12 visit, ii) tertiary objective c) changing from a proportion to time to event outcome iii) simplified description of procedures for invalid point-of-care results
2	2.0	24 June 2020	Jienchi Dorward	1. Broader range for sample size (80-180) to allow more flexibility due to COVID-19 in this feasibility study

				2. Clarification that enhanced adherence counselling at a previous visit (rather than the current visit) is an exclusion.<br>3. Addition of randomisation stratified by first-line ART regimen at enrolment<br>4. Addition of stored urine samples at enrolment<br>5. Inclusion of validation of other point-of-care tests (including assays that use finger-prick capillary blood samples) that may improve HIV care, in addition to viral load assays.<br>6. Minor clarifications around i) the threshold of viraemia for retrospective HIV drug resistance testing and adherence counselling ii) the assays used for routine NHLS laboratory viral load testing iii) and the definition of the end of the study
