# Supplementary material for: Protocol for a randomised feasibility study of Point-Of-care HIV viral load testing to Enhance Re-suppression in South Africa: the POwER study
Source: BMJ Open. 2021 Feb 16;11(2):e045373. doi: 10.1136/bmjopen-2020-045373 (PMC7888322; doi:10.1136/bmjopen-2020-045373)
Supplement: Author's manuscript [file bmjopen-2020-045373.draft_revisions.pdf]

BMJ Open is committed to open peer review. As part of this commitment we make the peer review history of every article we publish publicly available.

When an article is published we post the peer reviewers' comments and the authors' responses online. We also post the versions of the paper that were used during peer review. These are the versions that the peer review comments apply to.

The versions of the paper that follow are the versions that were submitted during the peer review process. They are not the versions of record or the final published versions. They should not be cited or distributed as the published version of this manuscript.

BMJ Open is an open access journal and the full, final, typeset and author-corrected version of record of the manuscript is available on our site with no access controls, subscription charges or pay-per-view fees (http://bmjopen.bmj.com).

If you have any questions on BMJ Open's open peer review process please email info.bmjopen@bmj.com

BMJ Open

Protocol for a randomised feasibility study of Point-Of-care HIV viral load testing to Enhance Re-suppression in South Africa: The POwER study

Journal:	*BMJ Open*
Manuscript ID	bmjopen-2020-045373
Article Type:	Protocol
Date Submitted by the Author:	29-Sep-2020
Complete List of Authors:	Dorward, Jienchi; University of Oxford, Nuffield Department of Primary Care Health Sciences; Centre for the Aids Programme of Research in South Africa,<br>Sookrajh, Yukteshwar; eThekwini Municipality Health Unit<br>Ngobese, Hope; Ethekwini Municipality Health Unit<br>Lessells, Richard; Centre for the Aids Programme of Research in South Africa, ; University of KwaZulu-Natal, KwaZulu-Natal Research and Innovation Sequencing Platform (KRISP)<br>Sayed, Fathima; Centre for the Aids Programme of Research in South Africa,<br>Bulo, Elliot; eThekwini Municipality Health Unit<br>Moodley, P; University of KwaZulu-Natal, Virology<br>Samsunder, Natasha; Centre for the Aids Programme of Research in South Africa<br>Lewis, Lara; CAPRISA<br>Tonkin-Crine, Sarah ; University of Oxford, Nuffield Department of Primary Care Health Science<br>Drain, Paul; University of Washington, Department of Global Health; Havard Medical School, Department of Surgery, Massachusetts General Hospital<br>Hayward, Gail; University of Oxford, Nuffield Department of Primary Care Health Sciences<br>Butler, Christopher C.; University of Oxford,<br>Garrett, Nigel; 1. Centre for the AIDS Programme of Research in South Africa (CAPRISA),
Keywords:	HIV & AIDS < INFECTIOUS DISEASES, Molecular diagnostics < INFECTIOUS DISEASES, VIROLOGY, PRIMARY CARE

SCHOLARONE™
Manuscripts

I, the Submitting Author has the right to grant and does grant on behalf of all authors of the Work (as defined in the below author licence), an exclusive licence and/or a non-exclusive licence for contributions from authors who are: i) UK Crown employees; ii) where BMJ has agreed a CC-BY licence shall apply, and/or iii) in accordance with the terms applicable for US Federal Government officers or employees acting as part of their official duties; on a worldwide, perpetual, irrevocable, royalty-free basis to BMJ Publishing Group Ltd ("BMJ") its licensees and where the relevant Journal is co-owned by BMJ to the co-owners of the Journal, to publish the Work in this journal and any other BMJ products and to exploit all rights, as set out in our licence.

The Submitting Author accepts and understands that any supply made under these terms is made by BMJ to the Submitting Author unless you are acting as an employee on behalf of your employer or a postgraduate student of an affiliated institution which is paying any applicable article publishing charge ("APC") for Open Access articles. Where the Submitting Author wishes to make the Work available on an Open Access basis (and intends to pay the relevant APC), the terms of reuse of such Open Access shall be governed by a Creative Commons licence – details of these licences and which Creative Commons licence will apply to this Work are set out in our licence referred to above.

Other than as permitted in any relevant BMJ Author's Self Archiving Policies, I confirm this Work has not been accepted for publication elsewhere, is not being considered for publication elsewhere and does not duplicate material already published. I confirm all authors consent to publication of this Work and authorise the granting of this licence.

For peer review only

TITLE: Protocol for a randomised feasibility study of Point-Of-care HIV viral load testing to Enhance Re-suppression in South Africa: The POwER study

Authors: Jienchi Dorward[1,2], Yukteshwar Sookrajh[3], Hope Ngobese[3], Richard Lessells[2,4], Fathima Sayed[2], Elliot Bulo[3], Pravikrishnen Moodley[5], Natasha Samsunder[2], Lara Lewis[2], Sarah Tonkin-Crine[1,6], Paul K. Drain[7,8,9], Gail Hayward[1], Christopher C Butler[1], Nigel Garrett[2,10]

Affiliations:

1. Nuffield Department of Primary Care Health Sciences, University of Oxford, Oxford, UK
2. Centre for the AIDS Programme of Research in South Africa (CAPRISA), University of KwaZulu–Natal, Durban, South Africa.
3. eThekwini Municipality Health Unit, Durban, South Africa
4. KwaZulu-Natal Research and Innovation Sequencing Platform (KRISP), University of KwaZulu-Natal, Durban, South Africa
5. Department of Virology, University of KwaZulu-Natal and National Health Laboratory Service, Inkosi Albert Luthuli Central Hospital, KwaZulu-Natal, South Africa
6. NIHR Health Protection Research Unit in Healthcare Associated Infections and Antimicrobial Resistance, University of Oxford, Oxford, UK
7. Department of Global Health, Schools of Medicine and Public Health, University of Washington, Seattle, USA.
8. Department of Medicine, School of Medicine, University of Washington, Seattle, USA.
9. Department of Epidemiology, School of Public Health, University of Washington, Seattle, USA.
10. Discipline of Public Health Medicine, School of Nursing and Public Health, University of KwaZulu-Natal, Durban, South Africa

Corresponding Author:

Jienchi Dorward

Nuffield Department of Primary Care Health Sciences, University of Oxford, Radcliffe Primary Care Building, Radcliffe Observatory Quarter, Woodstock Road, Oxford, OX2 6GG; Email: jienchi.dorward@phc.ox.ac.uk

Word count: 3998/4000

For peer review only - http://bmjopen.bmj.com/site/about/guidelines.xhtml

Keywords: HIV, point-of-care, viral load, viral failure, primary care

For peer review only

For peer review only - http://bmjopen.bmj.com/site/about/guidelines.xhtml

ABSTRACT (299/300)

Introduction: Access to HIV viral load testing remains difficult for many people on antiretroviral therapy (ART) in low- and middle-income countries. Weak laboratory and clinic systems often delay the detection and management of viremia, which can lead to morbidity, drug resistance and HIV transmission. Point-of-care testing could become an important strategy to overcome these challenges. We aim to assess whether it is feasible to conduct a randomised trial of point-of-care viral load testing to manage viraemia.

Methods and analysis: We will conduct an open-label, single site, individually randomized, feasibility study of Point-Of-care HIV viral load testing to Enhance Re-suppression (POwER), in Durban, South Africa. We will enrol people living with HIV who are aged ≥18 years, receiving first-line ART but with recent viraemia ≥1000 copies/mL, and randomize them 1:1 to receive point-of-care viral load or standard laboratory viral load monitoring, after 12 weeks. All participants will continue to receive care from public sector healthcare workers following South African HIV management guidelines. Participants with persistent viraemia ≥1000 copies/ml will be considered for switching to second-line ART. We will compare the proportion in each study arm who achieve the primary outcome of viral suppression <50 copies/mL at 24 weeks after enrolment. Additional outcomes include proportions retained in the study, proportions with HIV drug resistance, time to viral load results, and time to switching to second-line ART. We will assess implementation of point-of-care viral load testing using process evaluation data, and through interviews and focus groups with healthcare workers.

Ethics and dissemination: University of Oxford Tropical Research Ethics Committee and the Biomedical Research Ethics Committee of the University of KwaZulu-Natal have approved the study. We will present results to stakeholders, and through conferences and open access, peer-reviewed journals.

Trial registration and status: Pan African Clinical Trials Registry: PACTR202001785886049, registered 21st January 2020.

For peer review only - http://bmjopen.bmj.com/site/about/guidelines.xhtml

ARTICLE SUMMARY

Strengths and limitations of this study

- POwER will provide new evidence on using point-of-care HIV viral load testing to improve the management of viraemia in low- and middle-income countries.
- The study will be conducted in a public health facility and will inform the utility and ease of implementation of point-of-care viral load testing in a routine clinical setting.
- The study is limited by a moderate sample size and may therefore have reduced power to detect an effect of point-of-care viral load testing on viral re-suppression.

For peer review only

For peer review only - http://bmjopen.bmj.com/site/about/guidelines.xhtml

INTRODUCTION

Initiating antiretroviral therapy (ART) to achieve viral load suppression amongst all people with HIV is crucial to achieve the Joint United Nations Programme on HIV/AIDS (UNAIDS) target of ending AIDS by 2030. However, poor adherence to ART and/or HIV drug resistance can lead to HIV viraemia, with associated increases in morbidity, mortality, onward HIV transmission and the development and spread of HIV drug resistance.[1] Viral load testing can identify viraemia and guide adherence counselling, and/or switching to second-line ART regimens if HIV drug resistance is suspected. However, in low- and middle-income countries (LMICs), the majority of ART is provided in primary care, where there is limited laboratory viral load capacity, weak clinic systems to act on viral load results and a paucity of evidence based interventions to improve adherence.[1-5] Poor adherence can be due to complex social and psychological issues (e.g. gender-based violence, alcohol use, migrant labour and long distances to clinics).[6] Therefore, multiple visits for blood draws and result review confers an additional burden for these vulnerable populations. Laboratory viral load results are often not reviewed until the next routine clinic visit after several months, by which time adherence problems may have worsened.[1-4]

Point-of-care viral load testing could allow same-day adherence counselling and switching to second-line ART, and more efficient, person-centred care by reducing the burden of clinic visits for review of blood results; thereby leading to faster viral load re-suppression. The World Health Organization (WHO) has approved the Xpert HIV-1 VL (Cepheid, Sunnyvale, USA)[7] and the m-PIMA HIV-1/2 VL (Abbott, Chicago, USA)[8] as quantitative point-of-care HIV viral load assays for use in LMICs. We recently performed the STREAM point-of-care viral load trial, in which 390 adults stable on ART for six months were randomised to receive either usual care with laboratory viral load testing, or care by an enrolled nurse and point-of-care viral load testing.[9] After 12 months, people in the intervention arm were more likely to have been referred into a community ART delivery programme, and had 14% higher retention in care and viral suppression.[10] However, this study excluded clinically unstable patients (only 5% had viraemia at enrolment), and point-of-care viral load testing was provided in a site laboratory with research staff providing clinical care. Therefore, we have inadequate data on the effect of point-of-care viral load testing amongst people with viraemia who are a vulnerable, priority population and could be difficult to enrol and follow up in trials. Furthermore, we do not know if point-of-care viral load testing can be used effectively in routine primary care services.

Objectives

We aim to assess:

For peer review only - http://bmjopen.bmj.com/site/about/guidelines.xhtml

1. The estimated effect size of point-of-care viral load testing compared to standard laboratory viral load testing on viral re-suppression <50 copies/ml after 24 weeks.
2. The feasibility of recruiting, randomising and following up patients in a randomised trial of point-of-care viral load testing to manage HIV viraemia.
3. The perceptions of staff and the practical changes in clinic systems that are required to implement point-of-care viral load testing in a primary care clinic in South Africa.

METHODS AND ANALYSIS

Trial design

This will be a an open-label, single site, individually randomized, feasibility study of point-of-care viral load testing for the management of HIV viraemia (Figure 1). We will also assess the implementation of point-of-care viral load testing using process evaluation data, in-depth interviews and focus group discussions.

Study setting

The study will take place at the Prince Cyril Zulu Clinic; a large public sector clinic run by the eThekwini Municipality Health Unit in central Durban, South Africa. The clinic manages approximately 11,000 people receiving ART and follows South African National Department of Health guidelines.[11] Since December 2019, South Africa has started replacing current non-nucleoside reverse transcriptase inhibitors (NNRTIs), with the new drug dolutegravir for first-line ART.[12 13] Dolutegravir is an integrase strand transfer inhibitor that, in high-income settings, has been found to have better tolerability, efficacy, and durability than regimens based on NNRTIs such as efavirenz.[12 14] Current first-line ART regimens at Prince Cyril Zulu Clinic are therefore tenofovir disoproxil fumarate, emtricitabine and efavirenz, or tenofovir disoproxil fumarate, lamivudine and dolutegravir.[11 12] Viral load testing is performed at six and twelve months after ART initiation, and annually thereafter. Similar to WHO guidelines, patients with viraemia are given enhanced adherence counselling, with a repeat viral load within three months. If viraemia persists on NNRTI-based regimens, then HIV drug resistance is more likely, and so a switch to second-line ART should be considered.[11 15] For dolutegravir-based regimens, HIV drug resistance is less likely, and so a switch to second-line ART is only recommended if there are signs of clinical or immunological failure, or after two years of persistent viraemia.[11]

Eligibility criteria

Eligible patients will be HIV-positive adults aged ≥18 years, receiving first-line NNRTI- or dolutegravir-based ART, with the latest viral load in the past 6 weeks ≥1000 copies/ml, and with

For peer review only

no previous enhanced adherence counselling for this episode of viraemia. Pregnant women will not be eligible as they are routinely referred out of the Prince Cyril Zulu Clinic for antenatal care.

Study procedures

Recruitment

Potential participants will be identified by clinic staff using laboratory reports, electronic clinic information systems and clinic notes.

Informed consent, screening and enrolment

A research assistant or nurse will describe the study to potential participants, address any questions and seek written informed consent for participation. A research nurse will then assess eligibility using the patient's demographics, and a brief medical history and examination, including pregnancy testing for all women.

Randomisation and blinding

A statistician will generate the allocation sequence using random numbers generated in SAS 9.4 (SAS Institute Inc., Cary, USA). We will stratify randomisation by first-line ART regimen at enrolment (NNRTI-based or dolutegravir-based). The allocation sequence will contain variable block sizes, with participants randomised in a 1:1 ratio to the intervention or standard of care arm. All study staff apart from the statistician and data manager will be blinded to the allocation sequence. The allocation sequence will be programmed into the REDCap version 8.7.1 (Vanderbilt University, Nashville, USA)[16] enrolment electronic case report form (eCRF). At enrolment, the research assistant or research nurse will complete the enrolment eCRF, which will automatically assign the intervention allocation. As this is an open label study, study staff, clinic staff and participants will not be blinded to intervention allocation.

For peer review only

Baseline assessments

At enrolment, a nurse will administer a baseline sociodemographic questionnaire, and take a clinical history and examination, including an ART history. Throughout the study, South African Department of Health guidelines for managing viraemia will be used.[11] This includes enhanced adherence counselling, which will be performed by clinic staff with participants in both arms using the pre-enrolment viral load result. Techniques used during the counselling include ART education, evaluation of social support, mental health screening and the use of treatment supporters where appropriate.[17] A nurse will also draw blood for routine tests for investigation of

For peer review only - http://bmjopen.bmj.com/site/about/guidelines.xhtml

viraemia, which currently include CD4 count testing, hepatitis B surface antigen, creatinine and haemoglobin (Table 1).[11]

Follow-up

Participants in both arms will have routine clinic visits scheduled at the healthcare worker and participant's discretion, which is typically every 28 days to coincide with ART collection. At these visits, clinic staff will provide ongoing enhanced adherence guidelines, clinical support and ART when necessary. South African guidelines recommend a repeat viral load three months after the first high result ≥1000 copies/mL. As this study is taking place within a routine care setting, we anticipate that not all participants will attend within the three month visit window. In these cases we will allow repeat viral load to be done at another time, at the attending clinician's discretion.

Viral load testing interventions

For participants in the point-of-care arm, the repeat viral load test will be performed by a nurse, phlebotomist or laboratory technician in the clinic, using a WHO-approved, fully automated point-of-care viral load assay (such as the Xpert HIV-1 VL). Assay run times are ≤90 minutes. Clinic staff will be encouraged to provide results in the same clinic visit to inform ongoing management. If the participant cannot wait, or results are not available in the same visit, they will be available at the next clinic visit scheduled at staff and participant's discretion.

In the standard of care arm, samples for viral load testing will be transported on the same day to the National Health Laboratory Service (NHLS) for testing using their routine laboratory assays (e.g. Alinity m HIV-1 assay [Abbott, Chicago, USA]). Results are normally available after 7 days and will be provided to the participant at the next clinic visit, arranged at the participants and healthcare worker's discretion (typically after 7-28 days for results, depending on the participant's availability, ART supply and clinic schedules).

Management of viral load results at routine follow-up clinic visits

Viral load results will be managed by clinic staff in accordance with South African guidelines, which include guidance for management of both efavirenz and dolutegravir-based regimens, and do not advise routine HIV drug resistance testing.[18] Participants with a viral load ≥1000 copies/ml may meet criteria for viral failure (two consecutive viral loads ≥1000 copies/mL over 3 months apart) and will be considered for a switch to second-line ART. Switching to second-line ART and the choice of regimen will be at the clinician's discretion and will depend on the participant's first-line ART regimen, previous ART history, participant's preferences, perceived

For peer review only - http://bmjopen.bmj.com/site/about/guidelines.xhtml

adherence and current guidelines.[18] Participants with a second viral load <1000 copies/mL will remain on first-line ART. In accordance with guidelines for the transition to dolutegravir, those receiving an NNRTI may have this switched to a dolutegravir-based first-line regimen.[11]

Data capture during routine clinic visits

Research staff will monitor participants' clinical records to capture visit dates, whether enhanced adherence counselling was performed, medication prescriptions including ART, and laboratory results.

Study Exit Visit

The study exit visit will occur at 24 weeks (visit window 22-30 weeks), after which participants will continue receiving standard care at the Prince Cyril Zulu Clinic.

Participant Reimbursement

We will reimburse study participants ZAR150 (approximately GBP8.00) after the enrolment visit and ZAR100 (GBP5.50) after the 24 week visit, to cover any additional time spent at the clinic and any inconvenience due to study participation.[19]

Extended follow up of routine data

For participants who provide additional consent, research staff may access routine medical records for up to five years after the study exit visit, in order to determine longer term retention in care, ART adherence and viral load results. For participants who are lost to follow up, and who provided consent at enrolment, the participants mortality status may be checked on the South African National Population Register.

Additional laboratory specimens

Participants who provide additional consent will also have 10 mL of venous blood taken for storage and retrospective ART drug level and HIV drug resistance testing. Plasma will be stored at enrolment, during follow up alongside the repeat viral load test, and at study exit. In addition, at enrolment, 5 mL of urine will be taken for storage and ART drug level testing. We may use study samples to validate new point-of-care viral load, HIV drug resistance, or ART drug level assays. These results would not be used to guide clinical management.

Study outcomes

For peer review only - http://bmjopen.bmj.com/site/about/guidelines.xhtml

The primary outcome is viral suppression < 50 copies/mL at 24 weeks after enrolment, measured on a laboratory reference assay (e.g. Alinity m HIV-1) in both arms. Additional outcome measures are listed in Table 2.

Data collection and management

The research team will capture study data using standardised electronic CRFs in REDCap, which is a password protected data management service stored on a secure server.[16] All data entry will undergo three stages of quality control including pre-programmed data validity checks in electronic CRFs, immediate source document review, and weekly quality reports generated using REDCap. To protect confidentiality, all study-specific documents, other than the signed consent, will refer to the participant using their study participant number rather than their name. All documents will be stored in a secure cabinet in the locked research office and will only be accessible by study staff and authorised personnel.

Statistical methods

At the conclusion of the study, we will assess the proportion of participants achieving study outcomes in each arm, with 95% confidence intervals calculated using the Wilson method. We will use Fisher's exact test to compare the proportions achieving binary study outcome measures in each arm, using an intention to treat analysis. As this is a feasibility study, we may not be powered to test the hypotheses that there is a difference between the two arms for some outcome measures.

Primary outcome

We will calculate the proportion of participants in each arm achieving the binary primary outcome of viral suppression <50 copies/mL at 24 weeks (Table 2). Participants who are lost to follow up with no viral load result will be included in the analysis as not having achieved the primary outcome.

Secondary outcomes

We will calculate the proportion of potentially eligible patients with viraemia at the Prince Cyril Zulu clinic who are enrolled in the study (Table 3). The denominator will be the number of patients with viraemia during the study enrolment period, which will be calculated by clinic staff using NHLS high viral load reports. These reports are routinely used for monitoring and evaluation of clinic performance and include the number of first high viral loads ≥1000 copies/mL taken from the Prince Cyril Zulu Clinic each week. To assess study follow-up, we will

For peer review only - http://bmjopen.bmj.com/site/about/guidelines.xhtml

calculate the proportion of participants who attend the study exit visit at 24 weeks after enrolment. To assess implementation of point-of-care viral load testing, we will calculate the proportion of point-of-care viral load tests which are provided to participants on the same day, the proportion of viral loads ≥1000 copies/mL which resulted in same day enhanced adherence counselling, and the proportion of viral loads ≥1000 copies/mL resulting in appropriate same-day switch to second-line ART.

Tertiary Outcomes

In each arm we will assess the median number of days (and interquartile range) from enrolment to detection of viral failure, and from enrolment to appropriate switch to second-line ART (among participants with viral failure), and from enrolment to appropriate switching to dolutegravir (among participants on an NNRTI-based regimen). We will compare time to event outcomes using Cox proportional hazards. We will also calculate the proportion of participants with HIV drug resistance detected at 24 weeks after enrolment.

Sample size considerations

We conservatively estimate (based on our previous study)[9 10] that we could enroll approximately 100 participants in 6 months. However, the COVID-19 pandemic adds further uncertainty, as interruptions to care may decrease potential enrolment, or lead to higher numbers of people with viraemia during the study period.[20] Therefore, while we anticipate 100 participants, the final number may be between approximately 80-180 participants, and will be determined by the time and resources available. We will use NHLS, Prince Cyril Zulu Clinic and study data to assess outcomes, with estimated precision, in Table 3. Although not the primary aim of this feasibility study, assuming 50% of SOC participants achieve the main outcome of viral load suppression <50 copies/ml at 24 weeks, a sample size of 100 participants (50 per study arm) would provide 91% power to detect a +30% difference in the point-of-care testing arm, using a 2-sided alpha of 0.05. A sample size of 180 participants (90 per study arm) would provide 80% power to detect a +20% difference in the point-of-care testing arm, using a 2-sided alpha of 0.05. As all enrolled participants will be included in the final analysis, we have not adjusted for loss to follow up or potential withdrawals.

Quality assurance procedures

Study documentation will be subject to internal quality audits by the Centre for the AIDS Programme of Research (CAPRISA) Quality Assurance team, in accordance with CAPRISA standard operating procedures.

For peer review only - http://bmjopen.bmj.com/site/about/guidelines.xhtml

Qualitative sub-study

We will undertake a qualitative sub-study within POwER to assess what changes in clinic systems are required and what the views of staff are in implementing point-of-care viral load testing and managing viraemia. We will conduct semi-structured interviews and a focus group discussion with approximately 8-10 staff during, and then again after, implementation of point-of-care viral load testing in the study. Staff will include counsellors, phlebotomists, nurses, pharmacy staff, laboratory staff, doctors and health service managers. Topic guides will be informed by Normalisation Process Theory which aims to identify what is needed to 'normalise' use of a technology in a healthcare system.[21 22] Discussions will be transcribed and thematically analysed using NVIVO software (QSR International, Melbourne, Australia).

Patient and public involvement

Patients were not involved in the design of this study.

ETHICS AND DISSEMINATION

We will conduct the trial in accordance with the principles of the Declaration of Helsinki. The eThekwini Municipality Health Unit Research Committee, the KwaZulu-Natal Provincial Health Research Ethics Committee (KZ_202002_005), the University of KwaZulu-Natal Biomedical Research Ethics Committee (BREC/00000836/2019) and the University of Oxford Tropical Research Ethics Committee (OxTREC 66-19) have approved the study. We will present study results to front-line clinical workers and programme managers in eThekwini Municipality and KwaZulu-Natal Provincial Department of Health. We will also submit our findings to academic conferences and open-access, peer-reviewed journals.

Trial status

We registered POwER on the Pan African Clinical Trials Registry (PACTR202001785886049) on the 21st January 2020 and on the South African Clinical Trials (DOH-27-072020-6890) on the 17th July 2020. Enrolment started on 20th August 2020, under protocol version 2.0 dated 24th June 2020.

DISCUSSION

Evidence-based interventions to improve the management of viraemia are needed to achieve global HIV treatment targets, prevent the development and spread of HIV drug resistance, and improve patient outcomes. In this paper, we outline a feasibility trial of point-of-care viral load testing among people with viraemia who are receiving first-line ART in South Africa.

For peer review only - http://bmjopen.bmj.com/site/about/guidelines.xhtml

Problems with management of viraemia

Several studies highlight gaps in the management of viraemia in LMICs, and the resulting negative impacts on treatment outcomes. First, addressing potential poor adherence, which may have caused viraemia, is challenging. A meta-analysis of interventions to improve adherence did not find any strategies that resulted in better viral suppression in LMICs,[23] while a more recent cluster-randomised trial of the South African National Adherence Guidelines found no difference in long-term viral re-suppression among patients with viraemia.[5] Second, follow up viral load testing is often poorly performed. In the South African trial, less than 20% of patients had a repeat viral load within the recommended 3 months to confirm viral failure.[5] Similar delays have been reported in a large analysis of South African laboratory data; among 260,323 patients with viraemia, the median time to a repeat viral load was 6.2 months, (interquartile range [IQR] 4.0-11.1). Third, among people with confirmed viral failure, a systematic review,[24] and more recent individual studies,[4 25-27] have found that only 30-60% are switched to second-line ART. Even among patients who are switched, this can take a further 3 to 12 months,[25-27] with delays of even a few months being associated with increased risk of opportunistic infections and mortality, particularly among people with low CD4 counts.[1 4 25-27] Taken together, these data highlight the need for interventions to reduce delays and improve the management of viraemia.

Existing research regarding clinical effectiveness of point-of-care viral load testing

We conducted the STREAM trial, which was the first randomised trial of point-of-care HIV viral load testing for monitoring ART.[9 10] Overall, the STREAM intervention improved retention in care and viral suppression by 14.3% compared to the standard-of-care laboratory viral load arm. However, STREAM was designed to enroll stable patients; less than 5% of participants had viraemia at enrolment, and only 16 participants developed viral failure (seven in the point-of-care arm, nine in the standard care arm). Time to detection of viral failure was faster in the intervention arm (55 days [IQR 55–57] vs 123 days [IQR 98–162]) but numbers with viraemia were too small to determine whether point-of-care testing was effective at improving switching to second line ART, or subsequent viral load outcomes. In STREAM, the research team provided point-of-care testing and clinical care, so the observed benefits may also not be applicable to routine public sector settings.

Several more trials of point-of-care viral load testing are in progress, but none focus on management of viraemia. Studies in Zimbabwe[28 29], Kenya[30] and Haiti[31], are assessing point-of-care viral load for monitoring ART amongst pregnant women, children and/or adolescents. A

For peer review only - http://bmjopen.bmj.com/site/about/guidelines.xhtml

356 study in Nigeria is enrolling adults at ART initiation and will report on the effect of point-of-care
357 viral load testing after 12 months[32], while a trial in South Africa[33] is enrolling adults on ART who
358 are due for their annual viral load test. One trial in Uganda is assessing a viral load intervention
359 package that includes 'near point-of-care testing' (at the clinic or a nearby clinic), and is
360 enrolling 'high-risk groups' including patients with previous viraemia or no viral load in the past
361 year[34].

362 **Implementation of point-of-care viral load testing**

363 Point-of-care diagnostics have been used widely in healthcare systems in many LMICs.[35] In HIV
364 programmes, some tests such as rapid, lateral flow assays for diagnosis of HIV, have been
365 evaluated, endorsed and incorporated into WHO guidelines and successfully adopted in many
366 settings.[36] However, other assays, such as more complex molecular polymerase chain reaction
367 technologies for tuberculosis, have remained as laboratory tests despite being marketed as
368 point-of-care assays.[35] A large systematic review of barriers to point-of-care HIV diagnostic
369 implementation in LMICs found that in 132 studies, integration of the point-of-care test into
370 clinical work flows was the most commonly identified challenge to test utilization.[37] More
371 recently, studies have reported implementation of qualitative point-of-care viral load assays for
372 early infant diagnosis of HIV[38], and monitoring of ART in maternity wards in South Africa[39], and
373 de-centralised ART clinics in Malawi[40]. While these studies have reported quantitative outcomes
374 related to point-of-care viral load use, there is little qualitative work using theory-based
375 approaches to evaluate what is required for successful implementation.

376 **Strengths and limitations**

377 A major strength of this study is the focus on patients with viraemia, who are a priority
378 population at high risk of increased morbidity and mortality, onwards HIV transmission, and
379 development of HIV drug resistance.[1] Interventions to improve viral suppression amongst this
380 group have been prioritized by the WHO and align with several objectives in the Global Action
381 Plan on HIV Drug Resistance[41]. While our study may be under-powered to detect a significant
382 effect of point-of-care viral load testing on viral re-suppression, we will determine the feasibility
383 of conducting larger studies to answer this question in this setting. Our use of Normalisation
384 Process Theory will allow a rigorous assessment of what is needed to 'normalise' point-of-care
385 testing in the South African healthcare system.

386 Coinciding with the rollout of dolutegravir is both a strength and a weakness of our study. We
387 will enrol patients on either NNRTI- or dolutegravir-based ART, therefore allowing estimates of

For peer review only - http://bmjopen.bmj.com/site/about/guidelines.xhtml

the effect of point-of-care testing in either group. For patients enrolled on NNRTIs, this also presents an opportunity to assess whether point-of-care viral load testing can assist the transition to dolutegravir-based regimens. For patients enrolled on dolutegravir, a potential limitation is that point-of-care viral load testing will not be used to expedite a switch to second line, although it may still improve enhanced adherence counselling and allow more efficient care that can help ensure retention and virological suppression. Lastly, we will be able to assess early viral suppression outcomes and the frequency of HIV drug resistance among people with viraemia on dolutegravir. This is a pressing research question for global policy makers, because it determines the need for HIV drug resistance testing and second-line ART after introduction of a dolutegravir-based first-line in LMICs.

Conclusion

In the POwER study, we aim to provide evidence as to whether a trial of point-of-care viral load testing to manage viraemia is feasible, and how testing may be implemented in South African clinics. This work will contribute to the development of interventions to improve the management of viraemia amongst people receiving ART, thereby improving health and leading to longer and better lives, as well as reducing the spread of HIV and preventing drug resistance.

For peer review only

LIST OF ABBREVIATIONS

ART – antiretroviral therapy

UNAIDS - Joint United Nations Programme on HIV/AIDS

LMIC – low- and middle-income countries

WHO – World Health Organization

DECLARATIONS

Competing interests

Cepheid and Abbott have provided point-of-care viral load assays at no cost for use at the study site. The authors have no other competing interests to declare.

Funding

This work is supported by grants from the Wellcome Trust PhD Programme for Primary Care Clinicians (216421/Z/19/Z), the University of Oxford's Research England QR Global Challenges Research Fund (0007365) and the Africa Oxford Initiative (AfiOx-119). The University of Oxford is the study sponsor. The funders and sponsor have no role in study design, manuscript submission, or collection, management, analysis or interpretation of study data.

Author contributions

JD and NG conceived the study. YS, HN, RL, FS, EB, PM, NS, STC, PKD, GH and CB contributed to study design and implementation. STC contributed to design of the qualitative sub-study. JD wrote the first draft of the manuscript. All authors critically reviewed and edited the manuscript and consented to final publication.

Acknowledgements

The authors would like to thank all participants in the study and acknowledge the work and support of staff at the Prince Cyril Zulu Clinic, eThekwini Municipality, CAPRISA and the National Health Laboratory Services at Addington and Inkosi Albert Luthuli Hospitals.

Data sharing statement

Bona fide researchers will be able to request access to anonymised trial data by contacting the corresponding author.

For peer review only

For peer review only - http://bmjopen.bmj.com/site/about/guidelines.xhtml

REFERENCES

1. Murphy RA, Court R, Maartens G, et al. Second-line antiretroviral therapy in sub-Saharan Africa: It's time to mind the gaps. *AIDS Research and Human Retroviruses* 2017;33(12):1181-84. doi: 10.1089/aid.2017.0134
2. Labhardt ND, Ringera I, Lejone TI, et al. When patients fail UNAIDS' last 90 - the "failure cascade" beyond 90-90-90 in rural Lesotho, Southern Africa: a prospective cohort study. *Journal of the International AIDS Society* 2017;20(1):1-10. doi: 10.7448/IAS.20.1.21803
3. Etoori D, Ciglenecki I, Ndlangamandla M, et al. Successes and challenges in optimizing the viral load cascade to improve antiretroviral therapy adherence and rationalize second-line switches in Swaziland. *Journal of the International AIDS Society* 2018;21(10):e25194-e94. doi: 10.1002/jia2.25194
4. Hermans LE, Carmona S, Nijhuis M, et al. Virological suppression and clinical management in response to viremia in South African HIV treatment program: A multicenter cohort study. *PLoS Med* 2020;17(2):e1003037. doi: 10.1371/journal.pmed.1003037 [published Online First: 2020/02/26]
5. Fox MP, Pascoe SJS, Huber AN, et al. Effectiveness of interventions for unstable patients on antiretroviral therapy in South Africa: results of a cluster-randomised evaluation. *Tropical Medicine & International Health* 2018;23(12):1314-25. doi: 10.1111/tmi.13152
6. Shubber Z, Mills EJ, Nachega JB, et al. Patient-Reported Barriers to Adherence to Antiretroviral Therapy: A Systematic Review and Meta-Analysis. *PLOS Medicine* 2016;13(11):e1002183-e83. doi: 10.1371/journal.pmed.1002183
7. World Health Organization. WHO prequalification of in vitro diagnostics. Product : Xpert® HIV-1 Viral Load with GeneXpert® Dx, GeneXpert® Infinity- 48, GeneXpert® Infinity-48s and GeneXpert® Infinity-80, 2017.
8. World Health Organization. WHO Prequalification of In Vitro Diagnostics Public Report. Product: m-PIMA HIV-1/2 VL, 2019.
9. Dorward J, Garrett N, Quame-Amaglo J, et al. Protocol for a randomised controlled implementation trial of point-of-care viral load testing and task shifting: the Simplifying HIV TREAtment and Monitoring (STREAM) study. *BMJ Open* 2017;7(9):e017507-e07. doi: 10.1136/bmjopen-2017-017507
10. Drain PK, Dorward J, Violette LR, et al. Point-of-care HIV viral load testing combined with task shifting to improve treatment outcomes (STREAM): findings from an open-label, non-inferiority, randomised controlled trial. *Lancet HIV* 2020;7(4):e229-e37. doi: 10.1016/S2352-3018(19)30402-3 [published Online First: 2020/02/28]
11. South Africa National Department of Health. National Consolidated Guidelines for the Management of HIV in Adults, Adolescents, Children and Infants and Prevention of Mother-to-Child Transmission. Pretoria, South Africa: South African National Department of Health, 2020.
12. Dorward J, Lessells R, Drain PK, et al. Dolutegravir for first-line antiretroviral therapy in low-income and middle-income countries: uncertainties and opportunities for implementation and research. *The Lancet HIV* 2018;5(7):e400-e04. doi: 10.1016/S2352-3018(18)30093-6
13. Vitoria Marco HAFNFCCPVFRDDP. The transition to dolutegravir and other new antiretrovirals in low- and middle-income countries - what are the issues2018.
14. Hamers RL, Rinke de Wit TF, Holmes CB. HIV drug resistance in low-income and middle-income countries. *The Lancet HIV* 2018;5(10):e588-e96. doi: 10.1016/S2352-3018(18)30173-5

For peer review only - http://bmjopen.bmj.com/site/about/guidelines.xhtml

15. World Health Organization. Consolidated guidelines on the use of antiretroviral drugs for treating and preventing HIV infection: recommendations for a public health approach – 2nd edition. Geneva, Switzerland: World Health Organization, 2016:427-27.
16. Harris PA, Taylor R, Thielke R, et al. Research electronic data capture (REDCap)--a metadata-driven methodology and workflow process for providing translational research informatics support. *J Biomed Inform* 2009;42(2):377-81. doi: 10.1016/j.jbi.2008.08.010 [published Online First: 2008/10/22]
17. South Africa National Department of Health. Minimum package of interventions to support linkage to care, adherence and retention in care: South African National Department of Health, 2020.
18. The South African National Department of H. 2019 ART Clinical Guidelines for the management of HIV in Adults, Pregnancy, Adolescents, Children, Infants and Neonates. Pretoria, South Africa, 2019.
19. National Health Research Ethics C. Payment of trial participants in South Africa: Ethical considerations for Research Ethics Committees: South African Department of Health, 2012.
20. Madhi SA, Gray G, Ismail N, et al. COVID-19 lockdowns in low- and middle-income countries: Success against COVID-19 at the price of greater costs. *South African Medical Journal* 2020;110(8):724-26. doi: 10.7196/SAMJ.2020.v110i8.15055
21. May C, Finch T. Implementing, embedding, and integrating practices: An outline of normalization process theory. *Sociology* 2009;43(3):535-54. doi: 10.1177/0038038509103208
22. May CR, Mair F, Finch T, et al. Development of a theory of implementation and integration: Normalization Process Theory. *Implementation Science* 2009;4(1):29-29. doi: 10.1186/1748-5908-4-29
23. Kanters S, Park JJH, Chan K, et al. Interventions to improve adherence to antiretroviral therapy: a systematic review and network meta-analysis. *The Lancet HIV* 2017;4(1):e31-e40. doi: 10.1016/S2352-3018(16)30206-5
24. Ford N, Orrell C, Shubber Z, et al. HIV viral resuppression following an elevated viral load: a systematic review and meta-analysis. *Journal of the International AIDS Society* 2019;22(11):1-6. doi: 10.1002/jia2.25415
25. Rohr JK, Ive P, Robert Horsburgh C, et al. Marginal structural models to assess delays in second-line HIV treatment initiation in South Africa. *PLoS ONE* 2016;11(8):1-11. doi: 10.1371/journal.pone.0161469
26. Bell-Gorrod H, Fox MP, Boulle A, et al. The Impact of Delayed Switch to Second-Line Antiretroviral Therapy on Mortality, Depending on Definition of Failure Time and CD4 Count at Failure. *Am J Epidemiol* 2020;189(8):811-19. doi: 10.1093/aje/kwaa049 [published Online First: 2020/03/29]
27. Bell-Gorrod H, Court R, Schomaker M, et al. Increased Mortality With Delayed and Missed Switch to Second-Line Antiretroviral Therapy in South Africa. *J Acquir Immune Defic Syndr* 2020;84(1):107-13. doi: 10.1097/QAI.0000000000002313 [published Online First: 2020/02/08]
28. Munyati S. NCT03986099 Community Based Antiretroviral Therapy (CBART) Among Children on Chronic ART: ClinicalTrials.gov; 2019 [28 August 2020]. Available from: https://clinicaltrials.gov/ct2/show/NCT03986099 accessed 28 August 2020.
29. Clinton Health Access Initiative. NCT04048629 Impact of Point-of-care (POC) Viral Load (VL) Testing During Pregnancy in Zimbabwe ClinicalTrials.gov2019 [28 August 2020]. Available from: https://clinicaltrials.gov/ct2/show/NCT04048629 accessed 28 August 2020.

For peer review only

For peer review only - http://bmjopen.bmj.com/site/about/guidelines.xhtml

30. Patel R. NCT03820323 Optimizing Viral Load Suppression in Kenyan Children on Antiretroviral Therapy (Opt4Kids) ClinicalTrials.gov2019 [28 August 2020]. Available from: https://clinicaltrials.gov/ct2/show/NCT03820323 accessed 28 August 2020.
31. Fitzgerald D. NCT03288246: Point-of-care Viral Load Testing Among HIV-infected Adolescents in Haiti, 2017.
32. Meloni ST, Agbaji O, Chang CA, et al. The role of point-of-care viral load monitoring in achieving the target of 90 % suppression in HIV-infected patients in Nigeria : study protocol for a randomized controlled trial. 2019:1-11.
33. Theron G. NCT03187964: Xpert Ultra and Xpert HIV-VL in People Living With HIV (UltraHIV), 2017.
34. Jain V. NCT03553693: Rapid HIV Viral Load Monitoring in High Risk Patients In Uganda (RAPID-VL), 2018.
35. Jani IV, Peter TF. How Point-of-Care Testing Could Drive Innovation in Global Health. *The New England journal of medicine* 2013;368(24):2319-24. doi: 10.1056/NEJMsb1214197
36. Drain PK, Rousseau C. Point-of-care diagnostics: Extending the laboratory network to reach the last mile. *Current Opinion in HIV and AIDS* 2017;12(2):175-81. doi: 10.1097/COH.0000000000000351
37. Pai NP, Wilkinson S, Deli-Houssein R, et al. Barriers to Implementation of Rapid and Point-of-Care Tests for Human Immunodeficiency Virus Infection. *Point of Care* 2015;14(3):81-87. doi: 10.1097/POC.0000000000000056
38. Bianchi F, Cohn J, Sacks E, et al. Evaluation of a routine point-of-care intervention for early infant diagnosis of HIV: an observational study in eight African countries. *The Lancet HIV* 2019:1-9. doi: 10.1016/S2352-3018(19)30033-5
39. Kufa T, Mazanderani AH, Sherman GG, et al. Point-of-care HIV maternal viral load and early infant diagnosis testing around time of delivery at tertiary obstetric units in South Africa: a prospective study of coverage, results return and turn-around times. *J Int AIDS Soc* 2020;23(4):e25487. doi: 10.1002/jia2.25487 [published Online First: 2020/04/25]
40. Nicholas S, Poulet E, Wolters L, et al. Point-of-care viral load monitoring: outcomes from a decentralized programme in Malawi. *Journal of the International AIDS Society* 2019;22(8):1-9. doi: 10.1002/jia2.25387
41. World Health Organization. Global Action Plan for HIV Drug Resistance 2016-2021. Geneva, Switzerland, 2017.

For peer review only - http://bmjopen.bmj.com/site/about/guidelines.xhtml

TABLES

Table 1: Schedule of evaluation in the POwER study

	Enroll	Routine clinic visit follow-up[a]					Study exit
**Weeks in study**	**0**	**4**	**8**	**12**	**16**	**20**	**24[b]**
Informed consent	X						
Locator information	X						
Demographics	X						
Medical history	X						
Vital signs	X	X	X	X	X	X	X
Physical examination	X						
Urine pregnancy test	X						
Eligibility screen	X						
Enhanced adherence counselling	X	X	X	X	X[c]	X[c]	X[c]
Randomization	X						
Routine Department of Health testing [d]	X						
POC viral load (intervention arm)				X[e]			
Laboratory viral load (standard of care arm)				X[e]			
Reference viral load outcome measure							X
Evaluation of new POC assays[f]	X			X			X
Stored blood & HIV drug resistance testing[g]	X			X			X
Stored urine for ART drug level testing	X						

[a] Scheduled at participants and healthcare worker's discretion but typically every 28 days
[b] Study exit visit window 22-30 weeks
[c] Ongoing enhanced adherence counselling for participants with viral load ≥50 copies/mL

For peer review only - http://bmjopen.bmj.com/site/about/guidelines.xhtml

576 [d] CD4 count testing (4 mL EDTA), hepatitis B surface antigen (5 mL SST), creatinine (5 mL SST) and haemoglobin (4 mL EDTA)
578 [e] Repeat viral load testing recommended at 12 weeks but may be done any time during follow up at the attending clinician's discretion
580 [f] Maximum of 8 mL venous blood and/or a finger-prick capillary blood sample
581 [g] Retrospective drug resistance testing for viraemic participants at enrolment, follow up or study exit (10 mL EDTA)
583 ART = antiretroviral therapy, POC = point-of-care

584 **Table 2: Study objectives and outcomes in the POwER study**

**Objectives**	**Outcome Measures**	**Timepoint(s) of evaluation of this outcome measure (if applicable)**
**Primary Objective**<br>To estimate the effect of implementing point-of-care viral load testing versus standard laboratory viral load testing on the proportion of HIV positive participants with viraemia who subsequently achieve viral resuppression	The proportion of participants in each arm with viral load <50 copies/mL measured on laboratory reference assay	24 weeks after enrolment
**Secondary Objectives**<br>To assess whether it is feasible to perform a randomised implementation trial of point-of-care viral load testing to manage viraemia in a routine South African clinic, by determining:		
a) What proportion of people with HIV viraemia is feasible to enrol?	a) Proportion of patients with viraemia at the study clinic who are successfully enrolled in the study	a) During study enrolment period
b) What proportion of those enrolled is feasible to follow up?	b) Proportion of enrolled participants who attend the study exit visit	b) 24 weeks after enrolment

For peer review only - http://bmjopen.bmj.com/site/about/guidelines.xhtml

c) What proportion of point-of-care viral load results are received and acted on in the same day?	c1) The proportion of point-of-care viral load tests that are communicated to participants on the same day<br>AND<br>c2) The proportion of point-of-care viral load tests ≥1000 copies/mL that result in same day enhanced adherence counselling<br>AND<br>c3) The proportion of point-of-care viral load tests ≥1000 copies/mL that result in same day switch to second-line ART	c) 12 weeks after enrolment when point-of-care testing is performed
**Tertiary Objectives**<br>To estimate the effect of point-of-care viral load testing versus standard laboratory viral load testing on:		
a) Time to detection of viral failure (consecutive viral loads ≥1000 copies/mL) in each arm	a) Days from enrolment to availability of viral load result ≥1000 copies/mL	a) By 24 weeks after enrolment
b) Time to switch to second-line ART in each arm	b) Days from enrolment to appropriate switching to second-line ART, amongst participants with viral failure	b) By 24 weeks after enrolment
c) Appropriate switching to dolutegravir in each arm	c) Time to appropriate switching to dolutegravir in each arm	c) By 24 weeks after enrolment
d) HIV drug resistance in each arm	d) The proportion of participants in each arm with HIV drug resistance	d) 24 weeks after enrolment
**Qualitative Study Objectives:**<br>What changes in clinic systems are required and what are the views and experiences of staff in implementing point-of-care viral	Staff perspectives regarding implementation of point-of-care viral load testing in a routine clinic	During enrolment when point-of-care testing is being

For peer review only - http://bmjopen.bmj.com/site/about/guidelines.xhtml

load testing to manage viraemia?		implemented, and after study conclusion

Table 3: Study & process evaluation outcomes with precision estimates

Outcome	Estimated n/N, %	95% CI (Wilson)
Percentage with viral load <50 copies/ml at 24 weeks in POC arm	35/50, 70.0	56.2-80.9
Percentage with viral load <50 copies/ml at 24 weeks in SOC arm	25/50, 50.0	36.6-63.4
Percentage of eligible patients successfully enrolled in study	100/240*, 41.7	35.6-48.0
Percentage of those enrolled who are retained at 24 weeks	90/100, 90.0	82.6-94.5
Percentage in POC arm with same-day viral load testing	40/50, 79.5	70.0-88.8

*Assuming 240 eligible patients during enrolment period. POC = point-of-care, SOC = standard-of-care

For peer review only

For peer review only - http://bmjopen.bmj.com/site/about/guidelines.xhtml

591 **FIGURE LEGENDS**

592 Figure 1: Consort diagram of the POwER study

593 ART = antiretroviral therapy, VL = viral load

For peer review only

For peer review only - http://bmjopen.bmj.com/site/about/guidelines.xhtml

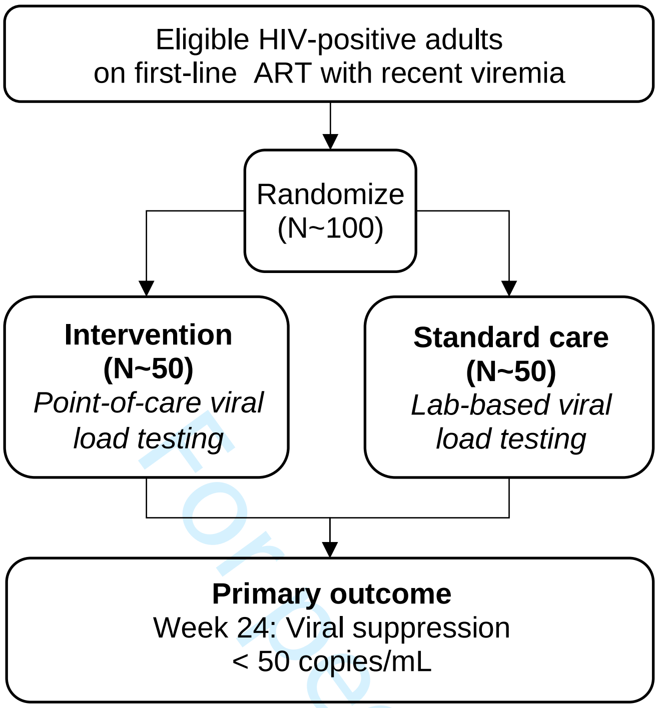

For peer review only

Study Title: POwER: Point Of care viral load testing to Enhance Re-suppression

Internal Reference Number / Short title: POwER

OxTREC Ref: 66-19

Date and Version No: Version 2.0 24th June 2020

**Chief Investigator:**	Dr Jienchi Dorward, Nuffield Department of Primary Care Health Sciences, University of Oxford
	Dr Nigel Garrett, Centre for the AIDS Programme of Research in South Africa, University of KwaZulu-Natal
**Investigators:**	Prof Chris Butler, Nuffield Department of Primary Care Health Sciences, University of Oxford
	Mrs Hope Ngobese, eThekwini Municipality Health Unit, eThekwini Municipality
	Dr Yukteshwar Sookrajh, eThekwini Municipality Health Unit, eThekwini Municipality
	Prof Gail Hayward, Nuffield Department of Primary Care Health Sciences, University of Oxford
	Dr Paul Drain, Department of Global Health, University of Washington
	Dr Richard Lessells, KwaZulu-Natal Research Innovation & Sequencing Platform, University of KwaZulu-Natal

**Sponsor:**	University of Oxford
**Funder:**	Internal Research England Global Challenges Research Fund, Wellcome Trust PhD Programme for Primary Care Clinicians, Africa Oxford Initiative.

Chief Investigator Signature:

Conflict of interest statement

We have no conflicts of interest to declare.

Confidentiality Statement

This document contains confidential information that must not be disclosed to anyone other than the authorised individuals from the University of Oxford, the Investigator Team and members of the Oxford Tropical Research Ethics Committee (OxTREC), unless authorised to do so.

For peer review only

TABLE OF CONTENTS

1. SYNOPSIS 4
2. ABBREVIATIONS 5
3. BACKGROUND AND RATIONALE 6
4. OBJECTIVES AND OUTCOME MEASURES 7
5. STUDY DESIGN 8
6. STUDY SETTING 9
7. PARTICIPANT IDENTIFICATION AND RECRUITMENT 9
7.1. Study Participants 9
7.2. Inclusion Criteria 9
7.3. Exclusion Criteria 10
8. STUDY PROCEDURES 10
8.1. Recruitment 10
8.2. Informed Consent 10
8.3. Screening and Eligibility Assessment 10
8.4. Randomisation, blinding and code-breaking 11
8.5. Baseline Assessments 11
8.5.1. Baseline demographics and medical history 11
8.5.2. Laboratory testing 11
8.6. Follow-up Routine Clinic Visits 11
8.6.1. Viral load testing at follow-up routine clinic visits 11
8.6.2. Management of viral load results at follow-up routine clinic visits 12
8.6.3. Data captures at routine clinic visits 12
8.7. Study Exit Visit 12
8.8. Extended follow up of routine data 13
8.9. Sample Handling 13
8.9.1. Point-of-care testing 13
8.9.2. Standard laboratory viral load testing 13
8.9.3. Other standard laboratory tests 13
8.9.4. Storage samples and HIV drug resistance testing 13
8.9.5. Potential evaluation of newer point-of-care assays 14
8.10. Discontinuation/Withdrawal of Participants from Study 14
8.11. Definition of End of Study 14

For peer review only

9. SAFETY REPORTING 14
10. STATISTICS AND ANALYSIS..... 14
10.1. Description of Statistical Methods 14
10.2. Analysis of Outcome Measures 15
10.2.1. Primary outcome 15
10.2.2. Secondary outcomes 15
10.2.3. Tertiary Outcomes 15
10.3. The Number of Participants 15
11. DATA MANAGEMENT 16
11.1. Access to Data 16
11.2. Data Handling and Record Keeping 16
12. QUALITATIVE STUDY..... 16
12.1. Background..... 16
12.2. Aim..... 16
12.3. Study design 17
12.4. Participants..... 17
12.5. Procedures..... 17
12.5.1. Informed consent 17
12.5.2. Interviews and focus groups..... 17
12.5.3. Analysis 17
13. QUALITY CONTROL AND QUALITY ASSURANCE PROCEDURES..... 17
14. ETHICAL AND REGULATORY CONSIDERATIONS..... 18
14.1. Declaration of Helsinki..... 18
14.2. Guidelines for Good Clinical Practice 18
14.3. Approvals..... 18
14.4. Participant Confidentiality..... 18
14.5. Expenses and Benefits..... 19
14.6. Reporting 19
14.7. Other Ethical Considerations..... 19
15. FINANCE AND INSURANCE 19
15.1. Funding 19
15.2. Insurance 20
16. PUBLICATION POLICY..... 20
17. REFERENCES 21

For peer review only

18. APPENDIX A: STUDY FLOW CHART 23
19. APPENDIX B: SCHEDULE OF STUDY PROCEDURES 24
20. APPENDIX C: SOUTH AFRICAN GUIDELINES FOR MANAGEMENT OF VIRAEMIA 25
21. APPENDIX D: SOUTH AFRICAN GUIDELINES FOR CHOICE OF SECOND-LINE ART 26
22. APPENDIX E: AMENDMENT HISTORY 26

1. SYNOPSIS

**Study Title**	POwER: Point Of care viral load testing to Enhance Re-suppression	
**Internal ref. no.**	POwER	
**Study Design**	This will be a single-site, open-label, individually randomized feasibility study	
**Study Participants**	HIV-positive adults aged ≥18 years, receiving first-line antiretroviral therapy (ART), with latest viral load in the past 6 weeks ≥1000 copies/ml, at the Prince Cyril Zulu Clinic in Durban, South Africa.	
**Planned Sample Size**	Approximately 100	
**Planned Study Period**	Two years	
	**Objectives**	**Outcome Measures**
**Primary**	To estimate the effect of implementing point-of-care viral load testing versus standard laboratory viral load testing on the proportion of HIV positive participants with viraemia who subsequently achieve viral resuppression	The proportion of participants in each arm with viral load <50 copies/mL after 24 weeks
**Secondary**	To assess whether it is feasible to perform a randomised implementation trial of point-of-care viral load testing to manage viraemia in a routine South African clinic, by determining:	
	a) What proportion of people with HIV viraemia is feasible to enrol?	a) Proportion of patients with viraemia at the study clinic who are enrolled in the study
	b) What proportion of those enrolled is feasible to follow up?	b) Proportion of enrolled participants who attend the 24 week study exit visit
	c) What proportion of point-of-care viral load results are received and acted on in the same day?	c) The proportion of point-of-care viral load tests that are communicated to participants on the same day, and the proportion resulting in same day enhanced adherence counselling and same day switch to second-line ART

For peer review only

**Tertiary Objectives**	To estimate the effect of point-of-care viral load testing versus standard laboratory viral load testing on:	
	a) Time to detection of viral failure (consecutive viral loads ≥1000 copies/mL) in each arm	a) Days from enrolment to availability of viral load result ≥1000 copies/mL
	b) Time to switch to second-line ART in each arm	b) Days from enrolment to appropriate switching to second-line ART, amongst participants with viral failure
	c) Dependent on dolutegravir rollout during the study period: Time to appropriate switching to dolutegravir in each arm	c) Days from enrolment to appropriate switching to dolutegravir-based ART according to South African guidelines
	d) HIV drug resistance in each arm	d) The proportion of participants in each arm with HIV drug resistance at 24 weeks
**Qualitative evaluation**	What changes in clinic systems are required and what are the views and experiences of staff in implementing point-of-care viral load testing to manage viraemia?	Staff perspectives regarding implementation of point-of-care viral load testing to manage viraemia

2. ABBREVIATIONS

BREC	Biomedical Research Ethics Committee
CAPRISA	Centre for the AIDS Programme of Research in South Africa
CI	Chief Investigator
CRF	Case Report Form
eGFR	Estimated glomerular filtration rate
GCP	Good Clinical Practice
HIV	Human Immunodeficiency Virus
ICF	Informed Consent Form
LMIC	Low- and middle-income country
MDRD	Modification of Diet in Renal Disease
NHLS	National Health Laboratory Service
OxTREC	Oxford Tropical Research Ethics Committee
PI	Principal Investigator
PIS	Participant Information Sheet
SOP	Standard Operating Procedure

For peer review only

WHO	World Health Organization

3. BACKGROUND AND RATIONALE

Initiating antiretroviral therapy (ART) to achieve viral load suppression amongst all people with HIV is crucial to achieve the UNAIDS target of ending AIDS by 2030. However, globally, 20% of people with HIV on ART have viraemia ≥1000 copies/mL, due to poor adherence and/or HIV drug resistance.[1] For these people, current World Health Organisation (WHO) guidelines recommend enhanced adherence counselling, with a repeat viral load within three months. If viraemia persists, HIV drug resistance is assumed, and second-line ART is recommended.[2,3] However, in low- and middle-income countries (LMICs), the majority of ART is provided in primary care, where there is limited laboratory viral load capacity, weak clinic systems to act on viral load results and a paucity of evidence based interventions to improve adherence.[4–9] Poor adherence can be due to complex social issues (e.g. sexual violence, alcohol use, migrant labour and long distances to clinics).[10] Therefore, multiple visits for blood draw, result review and repeated enhanced adherence counselling confers an additional burden for this vulnerable population. Laboratory viral load results are often not reviewed until the next routine clinic visit after several months, by which time adherence problems may have worsened.[4–9] Overall, these health system and individual factors result in ongoing viraemia, with associated increases in morbidity, mortality, onward HIV transmission and development and spread of HIV drug resistance.[4]

Point-of-care viral load testing could overcome some of these challenges by allowing a) immediate enhanced adherence counselling to address contemporary adherence b) same-day switching to second-line ART, and c) more efficient, person-centred care by reducing the burden of clinic visits for review of blood results; thereby leading to faster viral load re-suppression. WHO has approved the Xpert® HIV-1 VL (Cepheid, Sunnyvale, USA)[11] and the m-PIMA HIV-1/2 VL (Abbott, Chicago, USA)[12] as quantitative point-of-care HIV viral load assays for use in LMICs. The Xpert HIV-1 VL has been validated in multiple settings, including the proposed clinical site,[13] with good correlation and sensitivity of 96.5% and specificity of 96.6% to detect viraemia at a threshold of 1000 copies/mL.[14] This assay uses the GeneXpert platform which is widely available in LMICs for tuberculosis diagnostics. We performed the first pilot randomized controlled trial[15] of the Xpert HIV-1 VL, and demonstrated that in a research setting, point-of-care viral load testing helped to rapidly triage people with viral load suppression into more efficient care pathways. We found a 14% increase in retention in care and viral load suppression in the point-of-care testing arm compared to standard laboratory viral load testing.[15] However, this study **excluded clinically unstable patients** (only 5% had viraemia at enrolment). Therefore, we have inadequate data on the effect of point-of-care viral load testing amongst people with viraemia who are a vulnerable, priority population and could be difficult to enrol and follow up in trials. Furthermore, implementing point-of-care viral load testing requires multiple steps including assay maintenance, quality assurance and reorganisation of clinic flow, to ensure the result is used to provide immediate enhanced adherence counselling or switching of ART regimen in the same clinic visit, by the appropriate healthcare worker. In STREAM, research staff provided point-of-care viral load testing and clinical care, meaning that we do not know if implementation is feasible in routine primary care services. Therefore, we aim to test whether implementing point-of-care viral load testing to improve the management of viraemia is feasible, and whether the effect of point-of-care viral load testing on management of viraemia can be tested in a trial.

Lastly, many LMICs are now replacing current non-nucleoside reverse transcriptase inhibitors (NNRTIs)

For peer review only

with the new drug dolutegravir for first-line ART.[16] Dolutegravir is an integrase strand transfer inhibitor that, in high income settings, has been found to have better tolerability, efficacy, and durability than current NNRTI regimens. In South Africa, the rollout of dolutegravir is expected in late 2019/early 2020. Patients currently on NNRTI based first-line ART will require viral load testing to guide switching to dolutegravir-based first-line or second-line ART.[17] Our implementation study will likely coincide with the dolutegravir rollout, and presents an opportunity to assess whether point-of-care viral load testing can assist with the viral load informed transition to this newer regimen.

4. OBJECTIVES AND OUTCOME MEASURES

Objectives	Outcome Measures	Timepoint(s) of evaluation of this outcome measure (if applicable)
**Primary Objective**<br>To estimate the effect of implementing point-of-care viral load testing versus standard laboratory viral load testing on the proportion of HIV positive participants with viraemia who subsequently achieve viral resuppression	The proportion of participants in each arm with viral load <50 copies/mL measured on laboratory reference assay	24 weeks after enrolment
**Secondary Objectives**<br>To assess whether it is feasible to perform a randomised implementation trial of point-of-care viral load testing to manage viraemia in a routine South African clinic, by determining:		
a) What proportion of people with HIV viraemia is feasible to enrol?	a) Proportion of patients with viraemia at the study clinic who are successfully enrolled in the study	a) During study enrolment period
b) What proportion of those enrolled is feasible to follow up?	b) Proportion of enrolled participants who attend the study exit visit	b) 24 weeks after enrolment
c) What proportion of point-of-care viral load results are received and acted on in the same day?	c1) The proportion of point-of-care viral load tests that are communicated to participants on the same day<br>AND<br>c2) The proportion of point-of-care viral load tests ≥1000 copies/mL that result in same day enhanced adherence counselling<br>AND	c) 12 weeks after enrolment when point-of-care testing is performed

For peer review only

	c3) The proportion of point-of-care viral load tests ≥1000 copies/mL that result in same day switch to second-line ART	
**Tertiary Objectives** To estimate the effect of point-of-care viral load testing versus standard laboratory viral load testing on:		
a) Time to detection of viral failure (consecutive viral loads ≥1000 copies/mL) in each arm	a) Days from enrolment to availability of viral load result ≥1000 copies/mL	a) By 24 weeks after enrolment
b) Time to switch to second-line ART in each arm	b) Days from enrolment to appropriate switching to second-line ART, amongst participants with viral failure	b) By 24 weeks after enrolment
c) Dependent on dolutegravir rollout during the study period: Appropriate switching to dolutegravir in each arm	c) The proportion of participants in each arm appropriately switched to dolutegravir-based ART according to South African guidelines	c) 24 weeks after enrolment
d) HIV drug resistance in each arm	d) The proportion of participants in each arm with HIV drug resistance	d) 24 weeks after enrolment
**Qualitative Study Objectives:** What changes in clinic systems are required and what are the views and experiences of staff in implementing point-of-care viral load testing to manage viraemia?	Staff perspectives regarding implementation of point-of-care viral load testing in a routine clinic	During enrolment when point-of-care testing is being implemented, and after study conclusion

5. STUDY DESIGN

This will be a single-site, open-label, individually randomized feasibility study amongst people with recent HIV viraemia, comparing point-of-care viral load testing with standard laboratory viral load testing. We will also assess implementation of point-of-care viral load testing using process evaluation data, interviews and focus groups.

Eligible people will be HIV-positive, aged ≥18 years, receiving first-line ART, with latest viral load in the past 6 weeks ≥1000 copies/ml, and who have not received enhanced adherence counselling. Participants who provide written consent will be screened, enrolled and randomized in a 1:1 ratio to point-of-care viral load testing versus standard laboratory viral load testing (Appendix A). After randomization and during follow up, participants in both arms will continue to have routine clinic visits with clinical care, enhanced adherence counselling and blood draws from clinic staff as per South African and local guidelines, which recommend monthly visits until viral suppression is achieved. Repeat viral load testing will be performed at the clinician's discretion in accordance with South African guidelines, which recommend a repeat viral load after three months of enhanced adherence counselling. The Xpert® HIV-1 VL (or similar WHO approved assay e.g. m-PIMA HIV-1/2 VL) will be used in the point-of-care arm, and standard laboratory viral load testing in the standard of care arm. Clinic staff will be encouraged to use point-of-care viral load

For peer review only

results to provide immediate enhanced adherence counselling in the point-of-care arm, while in the SOC arm laboratory viral load results will be available to guide enhanced adherence counselling at the next clinic visit, which can be scheduled at the healthcare worker and client's discretion (typically after 28 days to coincide with next ART collection). Those with viral load ≥1000 copies/ml will meet criteria for viral failure (two consecutive viral loads ≥1000 copies/mL over 3 months apart), and will be managed according to South African viral failure guidelines, which recommend consideration of switching to a second-line ART regimen.[17] Those with viral loads <1000 copies/mL will continue their current first-line ART .[17] At the 24 week study exit visit a study outcome viral load will be measured using a laboratory reference assay (e.g. Alinity m HIV-1 or similar validated assay) for comparison between the two arms.

At enrolment, participants demographics, socioeconomic status and medical history will be recorded by research staff using standard questionnaires and review of clinical notes. During follow up, research staff will review participants clinic and laboratory notes to record time timing of receipt of viral load results, enhanced adherence counselling and switches to second-line ART. Data will be captured using electronic case report forms (CRFs) in REDCap.

During implementation of point-of-care viral load testing and after the study ends, a research assistant will perform in depth interviews and focus group discussions with clinic staff to explore implementation of point-of-care testing for management of viraemia (see section 12). Interviews and focus groups will be audio recorded, transcribed and analysed using Framework analysis.

6. STUDY SETTING

The study will take place at the Prince Cyril Zulu Clinic with support from the adjacent CAPRISA eThekwini Clinical Research Site. The Prince Cyril Zulu Clinic is a large public clinic situated next to the main transport hub in central Durban and provides HIV, tuberculosis sexual health and primary care services to a large, diverse, urban population. Prince Cyril Zulu Clinic sees approximately 40 people per month with viraemia ≥1000 copies/mL on first-line ART.

7. PARTICIPANT IDENTIFICATION AND RECRUITMENT

7.1. Study Participants

HIV-positive adults aged ≥18 years, receiving first-line ART, with latest viral load in the past 6 weeks ≥1000 copies/ml, and not having received enhanced adherence counselling.

7.2. Inclusion Criteria

The participant may enter the study if ALL of the following apply:

- Participant is willing and able to give informed consent for participation in the study.
- HIV positive adult, Male or Female, aged ≥18 years.
- Receiving first-line ART
- Latest viral load in the past 6 weeks ≥1000 copies/mL

For peer review only

7.3. Exclusion Criteria

The participant may not enter the study if ANY of the following apply:

- Has already received enhanced adherence counselling at a previous visit
- Requires hospital admission
- Pregnant

Pregnant women will not be enrolled in the study as they are routinely referred out of PCZ CDC for antenatal care. Participants who become pregnant during the study will be referred for antenatal care but will continue to be offered follow up in the study.

8. STUDY PROCEDURES

For peer review only

8.1. Recruitment

HIV-positive patients on first-line ART and with recent viraemia will be identified by clinic staff using National Health Laboratory Service (NHLS) 'High Viral Load Reports', electronic clinic information systems and clinic notes, and offered referral to the study team.

8.2. Informed Consent

A research assistant or research nurse/designee will approach patients at the clinic who have been identified as having a high viral load. The research assistant or research nurse/designee will describe the study, address any questions and ask for voluntary participation. If the participant is willing, they will be invited to a private room in the clinic where the research assistant or designee will present written and verbal versions of the Participant Information and Informed Consent detailing no less than: the exact nature of the study; what it will involve for the participant; the implications and constraints of the protocol; the known side effects and any risks involved in taking part. It will be clearly stated that the participant is free to withdraw from the study at any time for any reason without prejudice to future care, and with no obligation to give the reason for withdrawal.

The participant will be allowed as much time as they wish to consider the information, and the opportunity to question the Investigator or other independent parties to decide whether they will participate in the study. Written Informed Consent will then be obtained by means of participant dated signature and dated signature of the person who presented and obtained the Informed Consent. The person who obtained the consent must be suitably qualified and experienced, and have been authorised to do so by the Chief Investigator. A copy of the signed Informed Consent will be offered to the participant. The original signed form will be retained at the study site.

The participant must personally sign and date the latest approved version of the Informed Consent form before any study specific procedures are performed.

8.3. Screening and Eligibility Assessment

After informed consent has been obtained, a research nurse will review the study inclusion/exclusion criteria with the patient. Contact details, demographics, a medical history and vital signs will be recorded. The patients clinical and laboratory record will be reviewed. Pregnancy testing will be performed for all

women aged 18-55 who have not yet had a pregnancy test on that clinic visit. Patients deemed eligible for the study will be enrolled within 7 days of screening.

8.4. Randomisation, blinding and code-breaking

A CAPRISA statistician will generate the allocation sequence using random numbers generated in a statistical software program such as STATA 14.0 (Statacorp, Texas, USA) or SAS 9.4 (SAS Institute Inc., Cary, USA). Randomisation will be stratified by first-line ART regimen at enrolment (efavirenz-based or dolutegravir-based). The allocation sequence will contain variable block sizes, with participants randomised in a 1:1 ratio to the intervention or standard of care arm. The allocation sequence will be programmed into the REDCap (Vanderbilt University, Nashville, USA) enrolment electronic case report form (eCRF). All study staff apart from the statistician and data manager will be blinded to the allocation sequence. At enrolment, the research assistant or research nurse will complete the enrolment eCRF, which will automatically assign the intervention allocation and generate a unique participant identification number (PID). As this is an open label study, study staff, clinic staff and participants will not be blinded to intervention allocation.

8.5. Baseline Assessments

8.5.1. Baseline demographics and medical history

At enrolment, all participants will have a baseline sociodemographic questionnaire, clinical history including ART history, and examination. South African guidelines for clinical assessment and management of patients with a high viral load will be followed,[17] including enhanced adherence counselling performed by clinic staff using the pre-enrolment viral load result. In both arms, enhanced adherence counselling will follow South African guidelines, which include ART education, evaluation of social support, mental health screening and use of treatment supporters where appropriate.[18]

8.5.2. Laboratory testing

For all participants, a phlebotomist or nurse will draw venous blood for tests recommended by South African guidelines for investigation of viraemia, which currently include CD4 count testing, hepatitis B surface antigen, creatinine and haemoglobin. Venous blood for storage and retrospective HIV drug resistance testing will also be drawn.

8.6. Follow-up Routine Clinic Visits

Participants in both arms will have routine clinic visits scheduled at the healthcare worker and participants discretion, but normally every 28 days to coincide with ART collection. At these visits, clinic staff will provide ongoing enhanced adherence guidelines, clinical support and ART when necessary, as per the standard of care in South African National Guidelines. Clinicians will be able to request viral load testing as per South African guidelines, which recommend a repeat viral load 3 months after the first high result ≥1000 copies/mL.

8.6.1. Viral load testing at follow-up routine clinic visits

For participants randomised to the point-of-care arm, a phlebotomist or nurse will draw venous blood for point-of-care viral load testing in the clinic. Results should be available in approximately two hours. Staff will be encouraged to provide results in the same clinic visit to inform ongoing management. If results are

For peer review only - http://bmjopen.bmj.com/site/about/guidelines.xhtml

not available in the same visit, or if the client cannot wait, they will be available at the next clinic visit scheduled at staff and patient's discretion.

In the standard of care arm, a phlebotomist or nurse will draw venous blood for laboratory viral load testing which will be sent to the standard of care National Health Laboratory Service (NHLS) as per routine clinic procedures. Results are normally available after 7 days and will be provided to the participant at the next clinic visit, arranged at the participants and healthcare worker's discretion (typically after 7 -28 days for results, depending on the participants availability, ART supply and clinic schedules).

For participants in both arms, a venous blood sample will also be drawn for storage alongside the repeat viral load result.

8.6.2. Management of viral load results at follow-up routine clinic visits

Viral load results will be managed in accordance with South African Department of Health guidelines, which include guidance for management of both NNRTI and dolutegravir-based regimens (Appendix C).[17] Participants with a viral load ≥1000 copies/ml may meet criteria for viral failure (two consecutive viral loads ≥1000 copies/mL over 3 months apart) and will be considered for switch to second-line ART. Switch to second-line ART and the choice of regimen will be at the clinician's discretion and will depend on the participant's first-line ART, previous ART history, participant's preferences, perceived adherence and current South African national and local clinical guidelines (Appendices C and D).[17] The risks and benefits of potential new regimens will be discussed with the participant as per usual practice. Participants with a second viral load <1000 copies/mL will continue first-line ART, with enhanced adherence counselling for those with low level viraemia between 50-999 copies/mL (Table 1). If South Africa introduces dolutegravir during the study period, then study participants on NNRTI-based first-line ART with a suppressed viral load <50 copies/mL will be eligible for dolutegravir-based first-line ART.

Table 1: Management of viral load results during follow-up

HIV viral load result	Definition	Action
≥1000 copies/mL	Potential viral failure	Consider switch to second-line ART
50-999 copies/mL	Low level viraemia	Continue current first-line ART and enhanced adherence counselling
<50 copies/mL	Viral suppression	Continue first-line ART, if on NNRTI then may be eligible for dolutegravir based regimen.

8.6.3. Data captures at routine clinic visits

Research staff will monitor participants clinical records to capture visit dates, whether enhanced adherence counselling was performed, medication prescriptions including ART, and laboratory results.

8.7. Study Exit Visit

Clinical follow up will end at 24 weeks when a study outcome viral load will be measured using a laboratory reference assay (e.g. Alinity m HIV-1 or similar validated assay) for comparison between the two arms. A venous blood sample for storage and retrospective HIV drug resistance testing will also be taken. At 24 weeks, any participant who has not attended the study exit visit will be called by the research team and asked to attend. If by 30 weeks it has not been possible to contact the participant, they will be exited from the study.

For peer review only

8.8. Extended follow up of routine data

For participants who agree, research staff may access routine medical records, prescription data and laboratory results for up to five years after the study exit visit, in order to determine longer term retention in care, ART adherence and viral load results. For participants who are lost to follow up, and who provided consent at enrolment, the participants vital status may be checked on the South African National Population Register.

8.9. Sample Handling

8.9.1. Point-of-care testing

Point-of-care VL testing will be performed by a nurse, phlebotomist or technician in the clinic, who will have received comprehensive training on all aspects of performing point-of-care viral load testing and been assessed as competent by the CAPRISA laboratory site manager. All point-of-care viral load testing procedures will be performed in a separate, specially prepared room in the clinic, using a small centrifuge and the Xpert HIV-1 VL assay on the GeneXpert platform, or a similar WHO approved point-of-care assay (e.g. the m-PIMA HIV-1/2 VL). Testing will be performed following manufacturer instructions, using approximately 4 mLs of venous blood, drawn using aseptic technique. The sample will be centrifuged to separate approximately 1 mL of plasma which will be pipetted into the assay cartridge. The cartridge will be placed in the fully automated assay platform, with results available after approximately 90 minutes. In the case of an invalid result, a repeat blood sample may need to be drawn for point-of-care testing. If repeat point-of-care testing is not possible (e.g. machine failure) the repeat sample will be sent for standard laboratory testing. Error codes will be checked regularly and reported to the CAPRISA laboratory staff and manufacturer to identify and correct sample processing or machine errors.

8.9.2. Standard laboratory viral load testing

Laboratory viral load testing will be performed by a nurse or phlebotomist in the clinic according to standard clinic procedures. In brief, approximately 4 mLs of venous will be drawn using aseptic technique into an EDTA tube and transported on the same day to the National Health Laboratory Service for testing using their routine laboratory assays. Currently, the National Health Service performs viral load tests on the Alinity m HIV-1 assay (Abbott, Chicago, USA) at the Addington Hospital Laboratory, or the Inkosi Albert Luthuli Hospital Laboratory, depending on machine availability and capacity.

8.9.3. Other standard laboratory tests

At enrolment, approximately 20 mLs of venous blood will be drawn for CD4 count testing (4 mLs EDTA), hepatitis B surface antigen (5 mLs SST), creatinine (5 mLs SST) and haemoglobin (4 mLs EDTA). Samples will be transported on the same day to the National Health Laboratory Service for testing using their routine laboratory assays.

8.9.4. Storage samples and HIV drug resistance testing

Participants who provide additional consent will also have 10 mLs of venous blood for storage and retrospective ART drug level and HIV drug resistance testing, taken at enrolment, during follow up alongside the repeat viral load test, and at study exit. At enrolment, 5 mLs of urine will also be taken for storage and ART drug level testing. Samples will be stored in the CAPRISA biorepository according to Good Clinical Laboratory Practice. At the end of the study, HIV drug resistance testing will be performed on viraemic samples. The threshold of viraemia for HIV drug resistance testing will be determined by the likelihood of amplification on the assay being used. We will use standard Sanger sequencing or next-

For peer review only

generation sequencing with the Illumina MiSeq platform. Remaining blood will be stored for ten years after the end of the study and will be made available to the study team or other investigators who have obtained ethical approval for additional projects. After ten years, remaining stored samples will be disposed of.

8.9.5. Potential evaluation of newer point-of-care assays

If new point-of-care assays that may improve HIV care become available (such as viral load, HIV drug resistance, and ART drug level assays), we may use samples from this study to compare results from the new assay to the laboratory reference assay. At blood draw visits we may ask participants to provide up to 8 mLs venous blood and/or a finger-prick blood sample for evaluation of these new point-of-care assays. The new assay results would be for validation purposes only and would not be used to guide clinical management.

8.10. Discontinuation/Withdrawal of Participants from Study

Each participant has the right to withdraw from the study at any time. In addition, the Investigator may discontinue a participant from the study at any time if the Investigator considers it necessary for any reason including:

- Ineligibility (either arising during the study or retrospectively having been overlooked at screening)
- Significant protocol deviation
- Significant non-compliance with treatment regimen or study requirements
- Withdrawal of Consent

Participants who withdraw from the study will not be replaced. Data for participants who withdraw will be included in intention to treat analysis. The reason for withdrawal will be recorded in the Case Report Form.

8.11. Definition of End of Study

The end of study is the date 30 weeks after enrolment of the last participant.

9. SAFETY REPORTING

This study does not involve an investigational medicinal product or intervention that affects physiology, and therefore will not require serious adverse event reporting. However, for completeness we will report all cases of mortality in the study to the University of KwaZulu-Natal's Biomedical Research Ethics Committee (BREC).

10. STATISTICS AND ANALYSIS

10.1. Description of Statistical Methods

At the conclusion of the study, we will assess the proportion of participants achieving study outcomes in each arm, with 95% confidence intervals calculated using the Wilson method. We will use Fisher's exact test to compare the proportions achieving study outcome measures in each arm. However, as this is a

For peer review only

pilot study, we will not be powered to test the hypotheses that there is a difference in proportions between the two arms for most of our outcome measures.

10.2. Analysis of Outcome Measures

10.2.1. Primary outcome

We will calculate the proportion of participants in each arm achieving the binary primary outcome of viral suppression <50 copies/mL at 24 weeks (Table 2). Participants who are lost to follow up with no viral load result will be included the analysis as not having achieved the primary outcome, meaning all participants enrolled in each arm will be included in the analysis.

10.2.2. Secondary outcomes

We will calculate the proportion of patients with viraemia at the Prince Cyril Zulu clinic who are enrolled in the study. The denominator will be the number of patients with viraemia during the study enrolment period, which will be calculated by clinic staff using routinely generated NHLS high viral load reports. These reports are routinely used for monitoring and evaluation of clinic performance and include the number of first high viral loads > 1000 copies/mL taken from Prince Cyril Zulu each week. To assess study follow up, we will calculate the proportion of participants who attend the study exit visit at 24 weeks after enrolment. To assess implementation of point-of-care viral load testing, we will calculate the proportion of point-of-care viral load tests which are provided to participants on the same day, the proportion of viral loads ≥1000 copies/mL which resulted in same day enhanced adherence counselling, and the proportion of viral loads ≥1000 copies/mL resulting in appropriate same-day switch to second-line ART.

10.2.3. Tertiary Outcomes

We will assess the median number of days (and interquartile range) from enrolment to detection of viral failure, and from enrolment to appropriate switch to second-line ART, amongst participants with viral failure. We will also calculate the proportion of participants in each arm who are appropriately switched to a dolutegravir-based first or second line ART regimen by 24 weeks, and also the proportion of those with HIV drug resistance detected at 24 weeks after enrolment.

10.3. The Number of Participants

PCZC sees approximately 40 patients per month with viraemia on first-line ART. We conservatively estimate (based on our previous study) that we could recruit approximately 40% of these in 6 months, a total of 100 participants. However, as this is a feasibility study, the exact number that can be enrolled cannot be determined. The COVID-19 pandemic adds further uncertainty, as interruptions to care may decrease potential enrolment, or lead to higher numbers of people with viraemia during the study period, which could also increase enrolment. Therefore, while we anticipate 100 participants, the final number

Table 2: Study & process evaluation outcomes with precision estimates

Outcome	Estimated n/N, %	95% CI (Wilson)
Percentage with viral load <50 copies/ml at 24 weeks in POC arm	35/50, 70.0	56.2-80.9
Percentage with viral load <50 copies/ml at 24 weeks in SOC arm	25/50, 50.0	36.6-63.4
Percentage of eligible patients successfully enrolled in study	100/240*, 41.7	35.6-48.0
Percentage of those enrolled who are retained at 24 weeks	90/100, 90.0	82.6-94.5
Percentage in POC arm with same-day viral load testing	40/50, 79.5	70.0-88.8

*Assuming 240 eligible patients during enrolment period. POC = point-of-care, SOC = standard-of-care

For peer review only

may be between approximately 80-180 participants, and will be determined by the time and resources available. We will use NHLS, PCZC and study data to assess outcomes, with estimated precision, in Table 2. Although not the aim of this feasibility study, assuming 50% of SOC participants achieve the main outcome of viral load suppression <50 copies/ml at 24 weeks, a sample size of 50 participants per arm would provide 54% power to detect a +20% difference in the point-of-care testing arm, using a 2-sided alpha of 0.05. A difference of +10% would have a Newcombe-Wilson 95% confidence interval of -9 to +28%. A sample size of 90 participants per arm would provide 80% power to detect a +20% difference in the point-of-care testing arm, using a 2-sided alpha of 0.05. As all enrolled participants will be included in the final analysis, we have not adjusted for loss to follow up or potential withdrawals.

11. DATA MANAGEMENT

11.1. Access to Data

Direct access will be granted to authorised representatives from the University of Oxford and CAPRISA for monitoring and/or audit of the study to ensure compliance with regulations.

11.2. Data Handling and Record Keeping

The research team will capture study data using standardised electronic CRFs in REDCap. All data entry will undergo three stages of quality control including pre-programmed data validity checks in electronic CRFs, immediate source document review, and weekly quality reports generated by REDCap. The participants will be identified by a unique study specific number and/or code in any database. The name and any other identifying detail will NOT be included in any study data electronic file.

12. QUALITATIVE STUDY

12.1. Background

Point-of-care diagnostics have been used widely in healthcare systems in many low and middle income countries.[19] In HIV programmes, some tests such as rapid, lateral flow assays for diagnosis of HIV, have been evaluated, endorsed and incorporated into guidelines by the World Health Organization and successfully adopted in many settings.[20] However, other assays, such as more complex molecular polymerase chain reaction technologies for tuberculosis, have remained as laboratory tests despite being marketed as point-of-care assays.[19] A large systematic review of barriers to point-of-care HIV diagnostic implementation in low and middle income countries found that in 132 studies, integration of the point-of-care test into clinical work flows was the most commonly identified challenge to test utilisation.[21] However, there are few published implementation projects that have used a theory-based approach to assess implementation of point-of-care tests in healthcare systems, particularly in LMICs. One theory that has been widely used to assess implementation of new technologies in healthcare is Normalisation Process Theory. Normalisation Process Theory aims to identify what is needed to 'normalise' use of a technology in a healthcare system.

12.2. Aim

For peer review only

We aim to assess staff perspectives on implementation of point-of-care viral load testing for management of viraemia in a routine South African clinic.

12.3. Study design

During trial enrolment when implementation of point-of-care viral load testing is taking place, a trained research assistant with experience of qualitative research will perform the first round of semi-structured interviews with approximately 8-10 staff, and a focus group with approximately 8-10 staff. After completion of enrolment, we will perform a second round of interviews and focus group, to assess perspectives on implementation of point-of-care viral load testing and management of viraemia.

12.4. Participants

Approximately 10-15 staff involved in viral load testing and management of viraemia will be approached to participate in this qualitative study. Staff will include counsellors, phlebotomists, nurses, pharmacy staff, laboratory staff, doctors and health service managers. Focus groups and in-depth interviews may consist of the same or different staff members. However, we will endeavour to include the same staff members in the first and second round of interviews and focus groups.

12.5. Procedures

12.5.1. Informed consent

A trained research assistant will approach staff in the clinic, explain the nature of the project and offer participation. Should the staff member be interested, they will be taken to a private space and provided with written and verbal versions of the Qualitative Study Staff Participant Information Sheet and Informed Consent Form. The research assistant or other study team member will answer any questions that the staff member may have, and it will be clearly stated that staff are able to refuse to participate without adverse consequences for their working life. Once written informed consent has been provided, a copy will be offered to staff participant, and the original will remain with the research team.

12.5.2. Interviews and focus groups

Once the Informed Consent Form has been signed, the research assistant will use predetermined topic guides to conduct interviews and/or focus groups in a private room at the study clinic, or if necessary, at another suitable private venue of the participant's choice. Interviews will last approximately 30-60 minutes, and focus groups 45-75 minutes. We will make an audio recording of interviews and focus group discussions. If a participant does not consent to audio recording, we will take a written recording of the interview or focus group.

12.5.3. Analysis

Audio and written recordings will be anonymised and translated from isiZulu (when necessary) and transcribed. Data will be analysed using thematic analysis as described by Attride-Stirling and reported accordingly.[22]

13. QUALITY CONTROL AND QUALITY ASSURANCE PROCEDURES

For peer review only

Study documentation including participant information sheets, informed consent forms, regulatory files, standard operating procedures, source documentation and electronic CRFs will be subject to internal quality audits by the CAPRISA Quality Assurance team, in accordance with CAPRISA standard operating procedures. CAPRISA routinely measure quality control and retention rates on all studies. The study will be conducted in accordance with relevant regulations and standard operating procedures.

14. ETHICAL AND REGULATORY CONSIDERATIONS

14.1. Declaration of Helsinki

The Investigator will ensure that this study is conducted in accordance with the principles of the Declaration of Helsinki.

14.2. Guidelines for Good Clinical Practice

The Investigator will ensure that this study is conducted in accordance with relevant regulations and with Good Clinical Practice.

14.3. Approvals

The protocol, participant information sheet and informed consent form, topic guides and any proposed advertising material will be submitted to OxTREC and BREC for written approval.

The Investigator will submit and, where necessary, obtain approval from the above parties for all amendments to the original approved documents.

14.4. Participant Confidentiality

The study staff will ensure that the participants' anonymity is maintained. The participants will be identified only by a participant ID number on all study documents and any electronic database. All documents will be stored in a secure cabinet in the locked research office, and will only accessible by study staff and authorised personnel. The electronic data will be stored in REDCap, which is a password protected data management service. All computers with access to the data will be password protected. The study will comply with the General Data Protection Regulation (GDPR), which requires that personal data must not be kept as identifiable data for longer than necessary for the purposes concerned.

For the qualitative evaluation of implementation processes, interviews and focus group discussions will take place in a private room. We will make a written record during the focus group or interview, or from the recorded audio, but we will not write down any information that might identify participants or others. For example, if the participant mentions names of colleagues, we will not write those names down. The recording and notes from interviews and focus group discussions will be stored in a locked cabinet and only the staff working on this study will be able to use them. We will destroy the recordings when the research is completed. Data will never be reported in a way that could potentially reveal a single participant's identity. If during interviews or focus groups a staff member reveals practice which is not in

For peer review only

line with professional standards and may cause harm to patients, we will discuss this with the staff member and the clinic manager.

14.5. Expenses and Benefits

Patient participants will be paid ZAR150 (approximately £8.00) after the enrolment visit and ZAR100 (approximately £5.50) after the 24 week visit for their participation in the research, in accordance with South African research guidelines.[23] Reasonable travel expenses for any visits additional to normal care will be reimbursed.

Staff participants will not be reimbursed as interviews or focus groups will take place during working hours, at a mutually convenient time that has been agreed with the clinic manager.

14.6. Reporting

The CI shall submit an Annual Progress Report to OxTREC and BREC on the anniversary of the date of approval of the study. In addition, the CI shall submit an End of Study Report to OxTREC and BREC within 12 months of completion of the study. If we extend follow up of routinely collected clinic and laboratory data, for up to five years after the last study exit visit, the study will remain under annual review by OxTREC and BREC while those data are being collected.

14.7. Other Ethical Considerations

All study participants will be HIV-positive and may face HIV-related stigma. Therefore, maintaining confidentiality is particularly important in this study (see section 14.4).

For staff interviews and focus groups, there is the possibility that they will feel unable to refuse participation, or that they will feel challenged at being interviewed about aspects of their work. While every effort will be made to maintain staff confidentiality, due to the small numbers of staff participating in the study it may be possible for superiors and colleagues to infer their identity indirectly. Therefore, the option to refuse participation without implications in their work will be made clear in the informed consent process. We will also warn staff about the risk of colleagues and superiors being able to infer their identity indirectly. Staff will not be asked questions which could threaten their work position or personal questions about their lives. Questions will focus work-related issues that are already likely to have been discussed in other settings, as the clinic team have implemented point-of-care viral load testing for management of viraemia.

15. FINANCE AND INSURANCE

15.1. Funding

This study is funded by a fellowship from Wellcome Trust PhD Programme for Primary Care Clinicians, the Internal Research England Global Challenges Research Fund (0007365) and the Africa Oxford Initiative.

For peer review only

15.2. Insurance

The University of Oxford has a specialist insurance policy in place which would operate in the event of any participant suffering harm as a result of their involvement in the research (Newline Underwriting Management Ltd, at Lloyd's of London).

16. PUBLICATION POLICY

Findings from this study will be presented at academic conferences and submitted to peer reviewed journals for Open Access publication in accordance with Wellcome Trust policies for Wellcome Trust funded research. Authorship and acknowledgements will follow the International Committee of Medical Journal Editors guidelines. Author accepted manuscripts will be posted on the investigators' institutional websites.

For peer review only

17. REFERENCES

1 Joint United Programme on HIV/AIDS (UNAIDS). Miles to Go: Global AIDS Update 2018. Geneva, 2018.

2 South African National Department of Health. National consolidated guidelines for the prevention of mother to child transmission of HIV (PMTCT) and the management of HIV in children, adolescents and adults. Pretoria, South Africa, 2015 http://www.sahivsoc.org/Files/ART Guidelines 15052015.pdf (accessed Feb 13, 2017).

3 World Health Organisation. Updated recommendations on first-line and second-line antiretroviral regimens and post-exposure prophylaxis and recommendations on early infant diagnosis of HIV: interim guidance. Geneva, 2018.

4 Murphy RA, Court R, Maartens G, Sunpath H. Second-line antiretroviral therapy in sub-Saharan Africa: It's time to mind the gaps. *AIDS Res Hum Retroviruses* 2017; **33**: 1181–4.

5 Labhardt ND, Ringera I, Lejone TI, *et al.* When patients fail UNAIDS' last 90 - the "failure cascade" beyond 90-90-90 in rural Lesotho, Southern Africa: a prospective cohort study. *J Int AIDS Soc* 2017; **20**: 1–10.

6 Lessells RJ. Virological monitoring – how it relates to adherence. In: 4th Southern African HIV Clinicians Society Conference. Johannesburg, 2018. http://www.sahivsoc2018.co.za/wp-content/uploads/2018/11/23B_Richard-Lessells-NV_VL-monitoring.pdf.

7 Jobanputra K, Parker LA, Azih C, *et al.* Impact and Programmatic Implications of Routine Viral Load Monitoring in Swaziland. *JAIDS J Acquir Immune Defic Syndr* 2014; **67**: 45–51.

8 Etoori D, Ciglenecki I, Ndlangamandla M, *et al.* Successes and challenges in optimizing the viral load cascade to improve antiretroviral therapy adherence and rationalize second-line switches in Swaziland. *J Int AIDS Soc* 2018; **21**: e25194.

9 Hermans LE, Tempelman HA, Carmona S, *et al.* High rates of viral resuppression on first-line ART after initial virological failure. In: Conference on Retroviruses and Opportunistic Infections. Boston, 2018. http://www.croiconference.org/sessions/high-rates-viral-resuppression-first-line-art-after-initial-virological-failure.

10 Shubber Z, Mills EJ, Nachega JB, *et al.* Patient-Reported Barriers to Adherence to Antiretroviral Therapy: A Systematic Review and Meta-Analysis. *PLOS Med* 2016; **13**: e1002183.

11 Dorward J, Drain PK, Garrett N. Point-of-care viral load testing and differentiated HIV care. *Lancet HIV* 2018; **5**: e8–9.

12 Bwana P, Ageng'o J, Danda J, Mbugua J, Handa A, Mwau M. Performance and Usability of mPIMATM HIV 1/2 Viral Load test in point of care settings in Kenya. *J Clin Virol* 2019; published online Oct. DOI:10.1016/j.jcv.2019.104202019.

13 Garrett NJ, Drain PK, Werner L, Samsunder N, Abdool Karim SS. Diagnostic Accuracy of the Point-of-Care Xpert HIV-1 Viral Load Assay in a South African HIV Clinic. *JAIDS J Acquir Immune Defic Syndr* 2016; **72**: e45–8.

14 Sacks JA, Fong Y, Gonzalez MP, *et al.* Performance of Cepheid Xpert HIV-1 viral load plasma assay to accurately detect treatment failure. *AIDS* 2019; **33**: 1881–9.

15 Drain PK, Dorward J, Violette L, *et al.* Point-of-care viral load testing improves HIV viral suppression and retention in care. In: Conference on Retroviruses and Opportunistic Infections.

For peer review only

Seattle, Washington, 2019.

16 Dorward J, Lessells R, Drain PK, *et al.* Dolutegravir for first-line antiretroviral therapy in low-income and middle-income countries: uncertainties and opportunities for implementation and research. *Lancet HIV* 2018; **5**: e400–4.

17 The South African National Department of Health. 2019 ART Clinical Guidelines for the management of HIV in Adults, Pregnancy, Adolescents, Children, Infants and Neonates. Pretoria, South Africa, 2019.

18 South African National Department of Health. Adherence Guidelines for HIV, TB and NCDs. Pretoria, 2016.

19 Jani I V., Peter TF. How Point-of-Care Testing Could Drive Innovation in Global Health. *N Engl J Med* 2013; **368**: 2319–24.

20 Drain PK, Rousseau C. Point-of-care diagnostics: Extending the laboratory network to reach the last mile. *Curr Opin HIV AIDS* 2017; **12**: 175–81.

21 Pai NP, Wilkinson S, Deli-Houssein R, *et al.* Barriers to Implementation of Rapid and Point-of-Care Tests for Human Immunodeficiency Virus Infection: Findings From a Systematic Review (1996-2014). *Point Care* 2015; **14**: 81–7.

22 Attride-Stirling J. Thematic networks: an analytic tool for qualitative research. *Qual Res* 2001; **1**: 385–405.

23 National Health Research Ethics Council (NHREC). Payment of trial participants in South Africa: Ethical considerations for Research Ethics Committees. South African Department of Health, 2012 http://www.nhrec.org.za/docs/Documents/Paymentconsiderations.pdf (accessed March 10, 2017).

For peer review only

18. APPENDIX A: STUDY FLOW CHART

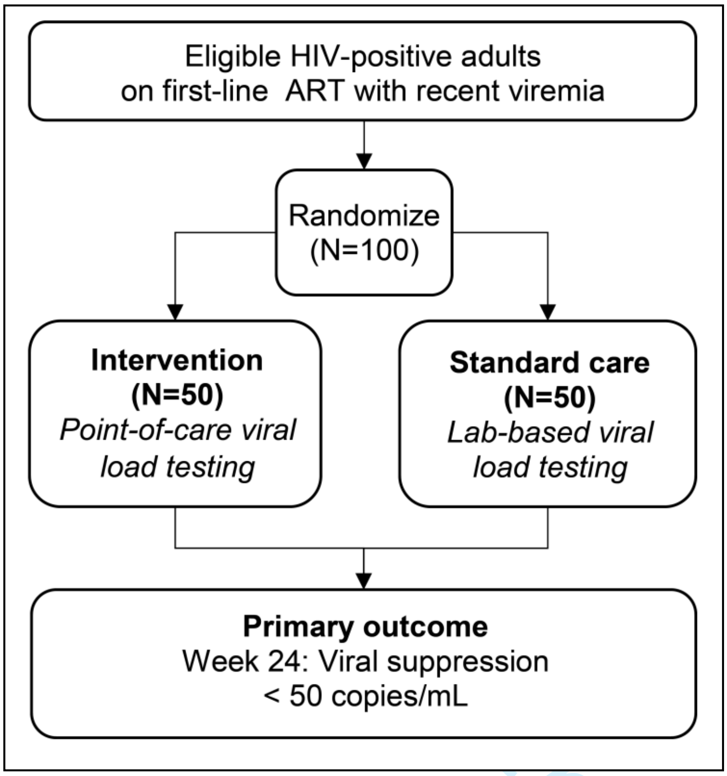

For peer review only

19. APPENDIX B: SCHEDULE OF STUDY PROCEDURES

	Enroll	Routine clinic visit follow-up[a]					Study exit
**Weeks in study**	**0**	**4**	**8**	**12**	**16**	**20**	**24**
Informed consent	X						
Locator information	X						
Demographics	X						
Medical history	X						
Vital signs	X	X	X	X	X	X	X
Physical examination	X						
Urine pregnancy test	X						
Eligibility screen	X						
Enhanced adherence counselling [b]	X	X	X	X	X[†]	X[†]	X[†]
Randomization	X						
Routine Department of Health testing [c]	X						
POC viral load (intervention arm)				X			
Laboratory viral load (standard of care arm)				X			
Reference viral load outcome measure							X
Evaluation of new POC assays[d]	X			X			X
Stored blood & HIV drug resistance testing[e]	X			X			X
Stored urine for ART drug level testing	X						

[a] Scheduled at participants and healthcare worker's discretion but typically every 28 days.
[b] Ongoing enhanced adherence counselling for participants with viral load ≥50 copies/mL.
[c] CD4 count testing (4 mLs EDTA), hepatitis B surface antigen (5 mLs SST), creatinine (5 mLs SST) and haemoglobin (4 mLs EDTA).
[d] Maximum of 8 mLs venous blood and/or a finger-prick capillary blood sample
[e§] Retrospective drug resistance testing for viraemic participants at enrolment, follow up or study exit (10 mLs EDTA)
ART = antiretroviral therapy, POC = point-of-care

For peer review only

20. APPENDIX C: SOUTH AFRICAN GUIDELINES FOR MANAGEMENT OF VIRAEMIA

Management of Viral Load Results in Infants, Children, Adolescents and Adults

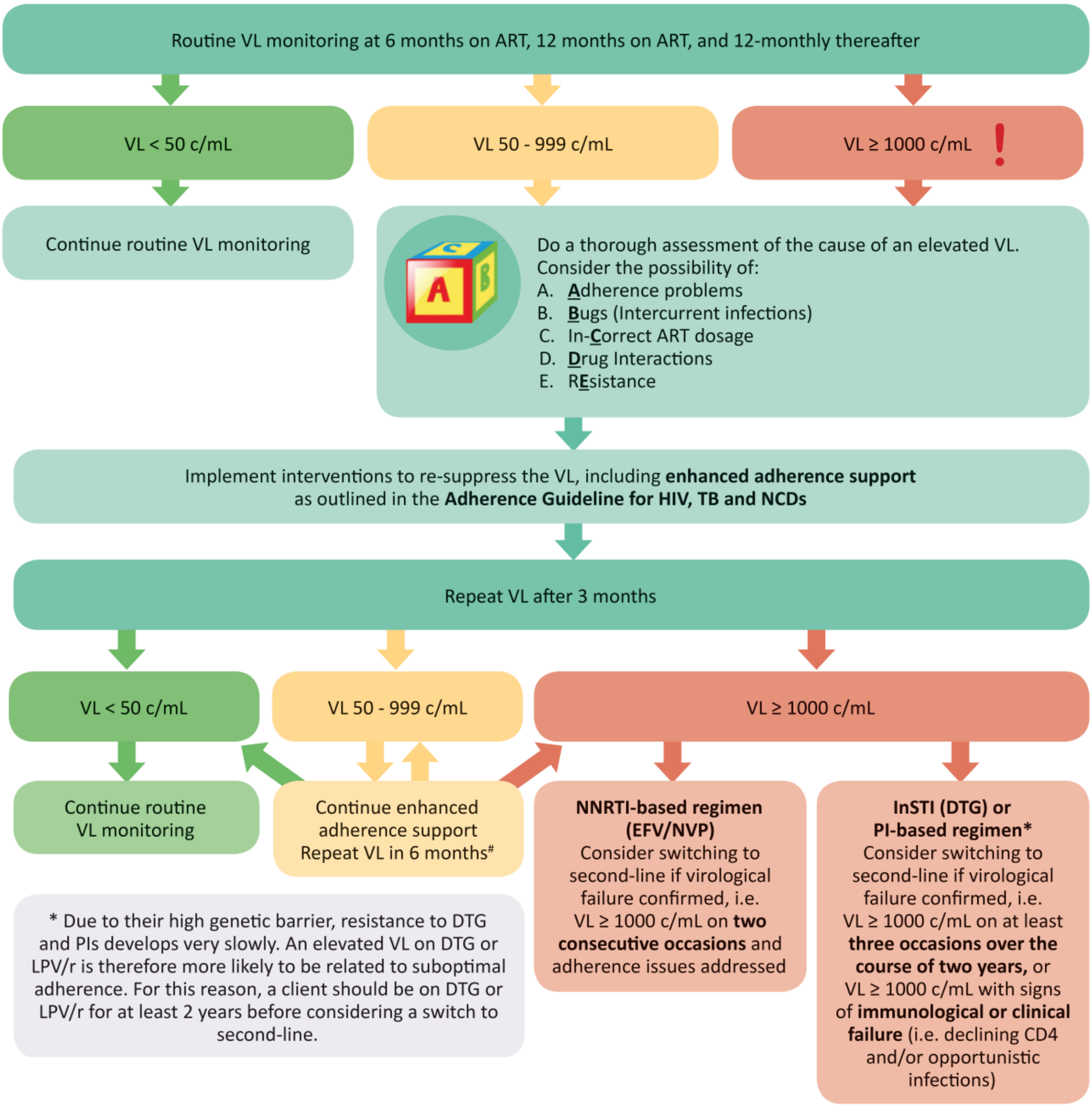

21. APPENDIX D: SOUTH AFRICAN GUIDELINES FOR CHOICE OF SECOND-LINE ART

Second-line ART Regimens for Adults with Confirmed Virological Failure

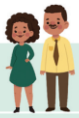

	First-Line Regimens			
	NNRTI-based Regimen		InSTI-based Regimen for > 2 years	
**Regimen**	**TDF + 3TC/FTC + EFV/NVP**		**TDF + 3TC/FTC + DTG**	
**Resistance Testing**	**Resistance test not required**		**Resistance testing may be required under expert consultation [5]**	
**Resistance Test results**	Not applicable		Not applicable	
**HBV Co-infection Status [1]**	**HBV-negative**	**HBV-positive**	**HBV-negative**	**HBV-positive**
**New Regimen**	**AZT + 3TC/FTC + DTG[2]**	**TDF[1] + AZT + 3TC/FTC + DTG[2]**	AZT + 3TC/FTC + LPV/r	TDF + 3TC/FTC + LPV/r [4]
	If DTG not suitable[2], AZT + 3TC/FTC + LPV/r	If DTG not suitable[2], TDF + 3TC + LPV/r[4]		

22. APPENDIX E: AMENDMENT HISTORY

Amendment No.	Protocol Version No.	Date issued	Author(s) of changes	Details of Changes made
1	1.1	10th November 2019	Jienchi Dorward	1. Addition of co-investigators Mrs H Ngobese and Dr Y Sookrajh<br>2. Addition of alternative, World Health Organization approved point-of-care viral load assays that could be used in the study<br>3. Addition of optional extended follow up of participants routinely collected clinic data and vital status in the South African National Population Register.<br>4. Change in the duration of blood storage from two to ten years<br>5. Addition of the potential evaluation of new point-of-care viral load assays<br>6. Minor clarifications regarding i) storage blood being drawn at week 12 visit, ii) tertiary objective c) changing from a proportion to time to event outcome iii) simplified description of procedures for invalid point-of-care results
2	2.0	24 June 2020	Jienchi Dorward	1. Broader range for sample size (80-180) to allow more flexibility due to COVID-19 in this feasibility study

For peer review only

				2. Clarification that enhanced adherence counselling at a previous visit (rather than the current visit) is an exclusion.<br>3. Addition of randomisation stratified by first-line ART regimen at enrolment<br>4. Addition of stored urine samples at enrolment<br>5. Inclusion of validation of other point-of-care tests (including assays that use finger-prick capillary blood samples) that may improve HIV care, in addition to viral load assays.<br>6. Minor clarifications around i) the threshold of viraemia for retrospective HIV drug resistance testing and adherence counselling ii) the assays used for routine NHLS laboratory viral load testing iii) and the definition of the end of the study

For peer review only

STANDARD PROTOCOL ITEMS: RECOMMENDATIONS FOR INTERVENTIONAL TRIALS

SPIRIT 2013 Checklist: Recommended items to address in a clinical trial protocol and related documents*

Section/item	Item No	Description	Addressed on page number
**Administrative information**			
Title	1	Descriptive title identifying the study design, population, interventions, and, if applicable, trial acronym	1
Trial registration	2a	Trial identifier and registry name. If not yet registered, name of intended registry	3 & 12
	2b	All items from the World Health Organization Trial Registration Data Set	3, 12, See PACTR
Protocol version	3	Date and version identifier	12
Funding	4	Sources and types of financial, material, and other support	16
Roles and responsibilities	5a	Names, affiliations, and roles of protocol contributors	1
	5b	Name and contact information for the trial sponsor	16
	5c	Role of study sponsor and funders, if any, in study design; collection, management, analysis, and interpretation of data; writing of the report; and the decision to submit the report for publication, including whether they will have ultimate authority over any of these activities	16
	5d	Composition, roles, and responsibilities of the coordinating centre, steering committee, endpoint adjudication committee, data management team, and other individuals or groups overseeing the trial, if applicable (see Item 21a for data monitoring committee)	NA

For peer review only

Introduction

Background and rationale	6a	Description of research question and justification for undertaking the trial, including summary of relevant studies (published and unpublished) examining benefits and harms for each intervention	5
	6b	Explanation for choice of comparators	5
Objectives	7	Specific objectives or hypotheses	5 & 6
Trial design	8	Description of trial design including type of trial (eg, parallel group, crossover, factorial, single group), allocation ratio, and framework (eg, superiority, equivalence, noninferiority, exploratory)	6

Methods: Participants, interventions, and outcomes

Study setting	9	Description of study settings (eg, community clinic, academic hospital) and list of countries where data will be collected. Reference to where list of study sites can be obtained	6
Eligibility criteria	10	Inclusion and exclusion criteria for participants. If applicable, eligibility criteria for study centres and individuals who will perform the interventions (eg, surgeons, psychotherapists)	6 & 7
Interventions	11a	Interventions for each group with sufficient detail to allow replication, including how and when they will be administered	8
	11b	Criteria for discontinuing or modifying allocated interventions for a given trial participant (eg, drug dose change in response to harms, participant request, or improving/worsening disease)	8
	11c	Strategies to improve adherence to intervention protocols, and any procedures for monitoring adherence (eg, drug tablet return, laboratory tests)	8
	11d	Relevant concomitant care and interventions that are permitted or prohibited during the trial	8
Outcomes	12	Primary, secondary, and other outcomes, including the specific measurement variable (eg, systolic blood pressure), analysis metric (eg, change from baseline, final value, time to event), method of aggregation (eg, median, proportion), and time point for each outcome. Explanation of the clinical relevance of chosen efficacy and harm outcomes is strongly recommended	9,10, Table 2
Participant timeline	13	Time schedule of enrolment, interventions (including any run-ins and washouts), assessments, and visits for participants. A schematic diagram is highly recommended (see Figure)	9, Table 1

For peer review only - http://bmjopen.bmj.com/site/about/guidelines.xhtml

Sample size	14	Estimated number of participants needed to achieve study objectives and how it was determined, including clinical and statistical assumptions supporting any sample size calculations	11
Recruitment	15	Strategies for achieving adequate participant enrolment to reach target sample size	7

Methods: Assignment of interventions (for controlled trials)

Allocation:

Sequence generation	16a	Method of generating the allocation sequence (eg, computer-generated random numbers), and list of any factors for stratification. To reduce predictability of a random sequence, details of any planned restriction (eg, blocking) should be provided in a separate document that is unavailable to those who enrol participants or assign interventions	7
Allocation concealment mechanism	16b	Mechanism of implementing the allocation sequence (eg, central telephone; sequentially numbered, opaque, sealed envelopes), describing any steps to conceal the sequence until interventions are assigned	7
Implementation	16c	Who will generate the allocation sequence, who will enrol participants, and who will assign participants to interventions	7
Blinding (masking)	17a	Who will be blinded after assignment to interventions (eg, trial participants, care providers, outcome assessors, data analysts), and how	7
	17b	If blinded, circumstances under which unblinding is permissible, and procedure for revealing a participant's allocated intervention during the trial	NA

Methods: Data collection, management, and analysis

Data collection methods	18a	Plans for assessment and collection of outcome, baseline, and other trial data, including any related processes to promote data quality (eg, duplicate measurements, training of assessors) and a description of study instruments (eg, questionnaires, laboratory tests) along with their reliability and validity, if known. Reference to where data collection forms can be found, if not in the protocol	10
	18b	Plans to promote participant retention and complete follow-up, including list of any outcome data to be collected for participants who discontinue or deviate from intervention protocols	10

Data management	19	Plans for data entry, coding, security, and storage, including any related processes to promote data quality (eg, double data entry; range checks for data values). Reference to where details of data management procedures can be found, if not in the protocol	10, 11
Statistical methods	20a	Statistical methods for analysing primary and secondary outcomes. Reference to where other details of the statistical analysis plan can be found, if not in the protocol	10, 11
	20b	Methods for any additional analyses (eg, subgroup and adjusted analyses)	10, 11
	20c	Definition of analysis population relating to protocol non-adherence (eg, as randomised analysis), and any statistical methods to handle missing data (eg, multiple imputation)	10, 11
**Methods: Monitoring**			
Data monitoring	21a	Composition of data monitoring committee (DMC); summary of its role and reporting structure; statement of whether it is independent from the sponsor and competing interests; and reference to where further details about its charter can be found, if not in the protocol. Alternatively, an explanation of why a DMC is not needed	NA
	21b	Description of any interim analyses and stopping guidelines, including who will have access to these interim results and make the final decision to terminate the trial	NA
Harms	22	Plans for collecting, assessing, reporting, and managing solicited and spontaneously reported adverse events and other unintended effects of trial interventions or trial conduct	NA
Auditing	23	Frequency and procedures for auditing trial conduct, if any, and whether the process will be independent from investigators and the sponsor	11
**Ethics and dissemination**			
Research ethics approval	24	Plans for seeking research ethics committee/institutional review board (REC/IRB) approval	12
Protocol amendments	25	Plans for communicating important protocol modifications (eg, changes to eligibility criteria, outcomes, analyses) to relevant parties (eg, investigators, REC/IRBs, trial participants, trial registries, journals, regulators)	12

For peer review only - http://bmjopen.bmj.com/site/about/guidelines.xhtml

Consent or assent	26a	Who will obtain informed consent or assent from potential trial participants or authorised surrogates, and how (see Item 32)	7
	26b	Additional consent provisions for collection and use of participant data and biological specimens in ancillary studies, if applicable	9
Confidentiality	27	How personal information about potential and enrolled participants will be collected, shared, and maintained in order to protect confidentiality before, during, and after the trial	10
Declaration of interests	28	Financial and other competing interests for principal investigators for the overall trial and each study site	16
Access to data	29	Statement of who will have access to the final trial dataset, and disclosure of contractual agreements that limit such access for investigators	16
Ancillary and post-trial care	30	Provisions, if any, for ancillary and post-trial care, and for compensation to those who suffer harm from trial participation	9
Dissemination policy	31a	Plans for investigators and sponsor to communicate trial results to participants, healthcare professionals, the public, and other relevant groups (eg, via publication, reporting in results databases, or other data sharing arrangements), including any publication restrictions	12
	31b	Authorship eligibility guidelines and any intended use of professional writers	12
	31c	Plans, if any, for granting public access to the full protocol, participant-level dataset, and statistical code	16
**Appendices**			
Informed consent materials	32	Model consent form and other related documentation given to participants and authorised surrogates	NA
Biological specimens	33	Plans for collection, laboratory evaluation, and storage of biological specimens for genetic or molecular analysis in the current trial and for future use in ancillary studies, if applicable	NA

*It is strongly recommended that this checklist be read in conjunction with the SPIRIT 2013 Explanation & Elaboration for important clarification on the items. Amendments to the protocol should be tracked and dated. The SPIRIT checklist is copyrighted by the SPIRIT Group under the Creative Commons "Attribution-NonCommercial-NoDerivs 3.0 Unported" license.

BMJ Open

Protocol for a randomised feasibility study of Point-Of-care HIV viral load testing to Enhance Re-suppression in South Africa: The POwER study

Journal:	*BMJ Open*
Manuscript ID	bmjopen-2020-045373.R1
Article Type:	Protocol
Date Submitted by the Author:	21-Jan-2021
Complete List of Authors:	Dorward, Jienchi; University of Oxford, Nuffield Department of Primary Care Health Sciences; Centre for the Aids Programme of Research in South Africa,<br>Sookrajh, Yukteshwar; eThekwini Municipality Health Unit<br>Ngobese, Hope; Ethekwini Municipality Health Unit<br>Lessells, Richard; Centre for the Aids Programme of Research in South Africa, ; University of KwaZulu-Natal, KwaZulu-Natal Research and Innovation Sequencing Platform (KRISP)<br>Sayed, Fathima; Centre for the Aids Programme of Research in South Africa,<br>Bulo, Elliot; eThekwini Municipality Health Unit<br>Moodley, P; University of KwaZulu-Natal, Virology<br>Samsunder, Natasha; Centre for the Aids Programme of Research in South Africa<br>Lewis, Lara; CAPRISA<br>Tonkin-Crine, Sarah ; University of Oxford, Nuffield Department of Primary Care Health Science<br>Drain, Paul; University of Washington, Department of Global Health; Havard Medical School, Department of Surgery, Massachusetts General Hospital<br>Hayward, Gail; University of Oxford, Nuffield Department of Primary Care Health Sciences<br>Butler, Christopher C.; University of Oxford,<br>Garrett, Nigel; 1. Centre for the AIDS Programme of Research in South Africa (CAPRISA),
**Primary Subject Heading**:	HIV/AIDS
Secondary Subject Heading:	Global health, Infectious diseases, Diagnostics
Keywords:	HIV & AIDS < INFECTIOUS DISEASES, Molecular diagnostics < INFECTIOUS DISEASES, VIROLOGY, PRIMARY CARE

I, the Submitting Author has the right to grant and does grant on behalf of all authors of the Work (as defined in the below author licence), an exclusive licence and/or a non-exclusive licence for contributions from authors who are: i) UK Crown employees; ii) where BMJ has agreed a CC-BY licence shall apply, and/or iii) in accordance with the terms applicable for US Federal Government officers or employees acting as part of their official duties; on a worldwide, perpetual, irrevocable, royalty-free basis to BMJ Publishing Group Ltd ("BMJ") its licensees and where the relevant Journal is co-owned by BMJ to the co-owners of the Journal, to publish the Work in this journal and any other BMJ products and to exploit all rights, as set out in our licence.

The Submitting Author accepts and understands that any supply made under these terms is made by BMJ to the Submitting Author unless you are acting as an employee on behalf of your employer or a postgraduate student of an affiliated institution which is paying any applicable article publishing charge ("APC") for Open Access articles. Where the Submitting Author wishes to make the Work available on an Open Access basis (and intends to pay the relevant APC), the terms of reuse of such Open Access shall be governed by a Creative Commons licence – details of these licences and which Creative Commons licence will apply to this Work are set out in our licence referred to above.

Other than as permitted in any relevant BMJ Author's Self Archiving Policies, I confirm this Work has not been accepted for publication elsewhere, is not being considered for publication elsewhere and does not duplicate material already published. I confirm all authors consent to publication of this Work and authorise the granting of this licence.

For peer review only

TITLE: Protocol for a randomised feasibility study of Point-Of-care HIV viral load testing to Enhance Re-suppression in South Africa: The POwER study

Authors: Jienchi Dorward[1,2], Yukteshwar Sookrajh[3], Hope Ngobese[3], Richard Lessells[2,4], Fathima Sayed[2], Elliot Bulo[3], Pravikrishnen Moodley[5], Natasha Samsunder[2], Lara Lewis[2], Sarah Tonkin-Crine[1,6], Paul K. Drain[7,8,9], Gail Hayward[1], Christopher C Butler[1], Nigel Garrett[2,10]

Affiliations:

1. Nuffield Department of Primary Care Health Sciences, University of Oxford, Oxford, UK
2. Centre for the AIDS Programme of Research in South Africa (CAPRISA), University of KwaZulu–Natal, Durban, South Africa.
3. eThekwini Municipality Health Unit, Durban, South Africa
4. KwaZulu-Natal Research and Innovation Sequencing Platform (KRISP), University of KwaZulu-Natal, Durban, South Africa
5. Department of Virology, University of KwaZulu-Natal and National Health Laboratory Service, Inkosi Albert Luthuli Central Hospital, KwaZulu-Natal, South Africa
6. NIHR Health Protection Research Unit in Healthcare Associated Infections and Antimicrobial Resistance, University of Oxford, Oxford, UK
7. Department of Global Health, Schools of Medicine and Public Health, University of Washington, Seattle, USA.
8. Department of Medicine, School of Medicine, University of Washington, Seattle, USA.
9. Department of Epidemiology, School of Public Health, University of Washington, Seattle, USA.
10. Discipline of Public Health Medicine, School of Nursing and Public Health, University of KwaZulu-Natal, Durban, South Africa

Corresponding Author:

Jienchi Dorward

Nuffield Department of Primary Care Health Sciences, University of Oxford,

Radcliffe Primary Care Building, Radcliffe Observatory Quarter, Woodstock Road, Oxford, OX2 6GG; Email: jienchi.dorward@phc.ox.ac.uk

Word count: 4376/4000

For peer review only - http://bmjopen.bmj.com/site/about/guidelines.xhtml

35 **Keywords:** HIV, point-of-care, viral load, viral failure, primary care

For peer review only

For peer review only - http://bmjopen.bmj.com/site/about/guidelines.xhtml

ABSTRACT (300/300)

Introduction: Access to HIV viral load testing remains difficult for many people on antiretroviral therapy (ART) in low- and middle-income countries. Weak laboratory and clinic systems often delay the detection and management of viremia, which can lead to morbidity, drug resistance and HIV transmission. Point-of-care testing could overcome these challenges. We aim to assess whether it is feasible to conduct a randomised trial of point-of-care viral load testing to manage viraemia.

Methods and analysis: We will conduct an open-label, single site, individually randomized, feasibility study of Point-Of-care HIV viral load testing to Enhance Re-suppression (POwER), in Durban, South Africa. We will enrol approximately 100 people living with HIV who are aged ≥18 years, receiving first-line ART but with recent viraemia ≥1000 copies/mL, and randomize them 1:1 to receive point-of-care viral load or standard laboratory viral load monitoring, after 12 weeks. All participants will continue to receive care from public sector healthcare workers following South African HIV management guidelines. Participants with persistent viraemia ≥1000 copies/ml will be considered for switching to second-line ART. We will compare the proportion in each study arm who achieve the primary outcome of viral suppression <50 copies/mL at 24 weeks after enrolment. Additional outcomes include proportions retained in the study, proportions with HIV drug resistance, time to viral load results, and time to switching to second-line ART. We will assess implementation of point-of-care viral load testing using process evaluation data, and through interviews and focus groups with healthcare workers.

Ethics and dissemination: University of Oxford Tropical Research Ethics Committee and the Biomedical Research Ethics Committee of the University of KwaZulu-Natal have approved the study. We will present results to stakeholders, and through conferences and open-access, peer-reviewed journals.

Trial registration and status: Pan-African Clinical Trials Registry: PACTR202001785886049, registered 21st January 2020. Enrolment started on 20th August 2020.

For peer review only - http://bmjopen.bmj.com/site/about/guidelines.xhtml

ARTICLE SUMMARY

Strengths and limitations of this study

- POwER will provide new evidence to guide the development of interventions using point-of-care HIV viral load testing to improve the management of viraemia in low- and middle-income countries.
- The study will be conducted in a public health facility and will inform the utility and ease of implementation of point-of-care viral load testing in a routine clinical setting.
- The study is limited by a moderate sample size and limited power to detect an effect of point-of-care viral load testing on viral re-suppression.

For peer review only

INTRODUCTION

Initiating antiretroviral therapy (ART) to achieve viral load suppression amongst all people with HIV is crucial to achieve the Joint United Nations Programme on HIV/AIDS (UNAIDS) target of ending AIDS by 2030. However, poor adherence to ART and/or HIV drug resistance can lead to HIV viraemia, with associated increases in morbidity, mortality, onward HIV transmission and the development and spread of HIV drug resistance.[1] Viral load testing can identify viraemia and guide adherence counselling, and/or switching to second-line ART regimens if HIV drug resistance is suspected. However, in low- and middle-income countries (LMICs), the majority of ART is provided in primary care, where there is limited laboratory viral load capacity, weak clinic systems to act on viral load results and a paucity of evidence based interventions to improve adherence.[1-5] Poor adherence can be due to complex social and psychological issues (e.g. gender-based violence, alcohol use, migrant labour and long distances to clinics).[6] Therefore, multiple visits for blood draws and result review confers an additional burden for these vulnerable populations. Laboratory viral load results are often not reviewed until the next routine clinic visit after several months, by which time adherence problems may have worsened.[1-4]

Point-of-care viral load testing could allow same-day adherence counselling and switching to second-line ART, and more efficient, person-centred care by reducing the burden of clinic visits for review of blood results; thereby leading to faster viral load re-suppression. The World Health Organization (WHO) has approved the Xpert HIV-1 VL (Cepheid, Sunnyvale, USA)[7] and the m-PIMA HIV-1/2 VL (Abbott, Chicago, USA)[8] as accurate, quantitative point-of-care HIV viral load assays for use in LMICs. However, these assays have not yet been rolled out for ART monitoring in low- and middle-income countries, due to concerns around implementation, and with limited randomised trial evidence to support clinical effectiveness. To date there is only one, single-site, randomised trial that has evaluated the clinical effectiveness of these assays. This was the STREAM point-of-care viral load trial, conducted by our team, in which 390 adults stable on ART for six months were randomised to receive either usual care with laboratory viral load testing, or care by an enrolled nurse and point-of-care viral load testing.[9] After 12 months, people in the intervention arm were more likely to have been referred into a community ART delivery programme, and had 14% higher retention in care and viral suppression.[10] However, the combined intervention was provided by research staff, meaning we do not know if point-of-care viral load assays can be implemented effectively in public sector clinics in low- and middle-income countries[11]. Furthermore, STREAM only enrolled clinically stable patients (5% had

For peer review only - http://bmjopen.bmj.com/site/about/guidelines.xhtml

viraemia at enrolment), and so there is inadequate data on the effect of point-of-care viral load testing amongst people with viraemia who are a vulnerable, priority population.

Objectives

The overall aim of this feasibility study[12] is to provide exploratory estimates to guide the development of a future larger trial of point-of-care testing to manage HIV viraemia. Our specific objectives are to:

1. Broadly estimate the effect size of point-of-care viral load testing compared to standard laboratory viral load testing on viral re-suppression <50 copies/ml after 24 weeks.
2. Determine the feasibility of recruiting, randomising and following up patients in a randomised trial of point-of-care viral load testing to manage HIV viraemia.
3. Assess the perceptions of staff and the practical changes in clinic systems that are required to implement point-of-care viral load testing for a subset of patients in a primary care clinic in South Africa.

METHODS AND ANALYSIS

Trial design

This will be a an open-label, single site, individually randomized, feasibility study of point-of-care viral load testing for the management of HIV viraemia (Figure 1). We aim to enrol approximately 100 adults receiving first-line ART and who have viraemia, and randomise them in a 1:1 ratio to receive point-of-care versus standard laboratory-based viral load testing. We will assess viral suppression after 24 weeks in each arm, and estimate recruitment, fidelity of the intervention and retention in care. We will also assess the implementation of point-of-care viral load testing using process evaluation data, in-depth interviews and focus group discussions.

Study setting

The study will take place at the Prince Cyril Zulu Clinic; a large public sector clinic run by the eThekwini Municipality Health Unit in central Durban, South Africa. The clinic manages approximately 11,000 people receiving ART and follows South African National Department of Health guidelines.[13] Since December 2019, South Africa has started replacing current non-nucleoside reverse transcriptase inhibitors (NNRTIs), with the new drug dolutegravir for first-line ART.[14 15] Dolutegravir is an integrase strand transfer inhibitor that, in high-income settings, has been found to have better tolerability, efficacy, and durability than regimens based on NNRTIs such as efavirenz.[14 16] Current first-line ART regimens at Prince Cyril Zulu Clinic are therefore tenofovir disoproxil fumarate, emtricitabine and efavirenz, or tenofovir disoproxil fumarate,

For peer review only - http://bmjopen.bmj.com/site/about/guidelines.xhtml

lamivudine and dolutegravir.[13 14] Viral load testing is performed at six and twelve months after ART initiation, and annually thereafter. Similar to WHO guidelines, patients with viraemia are given enhanced adherence counselling, with a repeat viral load within three months. If viraemia persists on NNRTI-based regimens, then HIV drug resistance is more likely, and so a switch to second-line ART should be considered.[13 17] For dolutegravir-based regimens, HIV drug resistance is less likely, and so a switch to second-line ART is only recommended if there are signs of clinical or immunological failure, or after two years of persistent viraemia.[13]

Eligibility criteria

Eligible patients will be HIV-positive adults aged ≥18 years, receiving first-line NNRTI- or dolutegravir-based ART, with the latest viral load in the past 6 weeks ≥1000 copies/ml, and with no previous enhanced adherence counselling for this episode of viraemia. Pregnant women will not be eligible as they are routinely referred out of the Prince Cyril Zulu Clinic for antenatal care.

Study procedures

Recruitment

Potential participants will be identified by clinic staff using laboratory reports, electronic clinic information systems and clinic notes.

Informed consent, screening and enrolment

A research assistant or nurse will describe the study to potential participants, address any questions and seek written informed consent for participation. A research nurse will then assess eligibility using the patient's demographics, and a brief medical history and examination, including pregnancy testing for all women.

Randomisation and blinding

A statistician will generate the allocation sequence using random numbers generated in SAS 9.4 (SAS Institute Inc., Cary, USA). We will stratify randomisation by first-line ART regimen at enrolment (NNRTI-based or dolutegravir-based). The allocation sequence will contain variable block sizes, with participants randomised in a 1:1 ratio to the intervention or standard of care arm. All study staff apart from the statistician and data manager will be blinded to the allocation sequence. The allocation sequence will be programmed into the REDCap version 8.7.1 (Vanderbilt University, Nashville, USA)[18] enrolment electronic case report form (eCRF). At enrolment, the research assistant or research nurse will complete the enrolment eCRF, which

For peer review only - http://bmjopen.bmj.com/site/about/guidelines.xhtml

will automatically assign the intervention allocation. As this is an open label study, study staff, clinic staff and participants will not be blinded to intervention allocation.

Baseline assessments

At enrolment, a nurse will administer a baseline sociodemographic questionnaire, and take a clinical history and examination, including an ART history. Throughout the study, South African Department of Health guidelines for managing viraemia will be used.[13] This includes enhanced adherence counselling, which will be performed by clinic staff with participants in both arms using the pre-enrolment viral load result. Techniques used during the counselling include ART education, evaluation of social support, mental health screening and the use of treatment supporters where appropriate.[19] A nurse will also draw blood for routine tests for investigation of viraemia, which currently include CD4 count testing, hepatitis B surface antigen, creatinine and haemoglobin (Table 1).[13]

Follow-up

Participants in both arms will have routine clinic visits scheduled at the healthcare worker and participant's discretion, which is typically every 28 days to coincide with ART collection. At these visits, clinic staff will provide ongoing enhanced adherence guidelines, clinical support and ART when necessary. South African guidelines recommend a repeat viral load three months after the first high result ≥1000 copies/mL. As this study is taking place within a routine care setting, we anticipate that not all participants will attend within the three month visit window. In these cases we will allow repeat viral load to be done at another time, at the attending clinician's discretion.

Viral load testing interventions

For participants in the point-of-care arm, the repeat viral load test will be performed by a nurse, phlebotomist or laboratory technician in the clinic, using a WHO-approved, fully automated point-of-care viral load assay (such as the Xpert HIV-1 VL). Assay run times are ≤90 minutes. Clinic staff will be encouraged to provide results in the same clinic visit to inform ongoing management. If the participant cannot wait, or results are not available in the same visit, they will be available at the next clinic visit scheduled at staff and participant's discretion.

In the standard of care arm, samples for viral load testing will be transported on the same day to the National Health Laboratory Service (NHLS) for testing using their routine laboratory assays (e.g. Alinity m HIV-1 assay [Abbott, Chicago, USA]). Results are normally available after 7 days and will be provided to the participant at the next clinic visit, arranged at the participants and

For peer review only - http://bmjopen.bmj.com/site/about/guidelines.xhtml

healthcare worker's discretion (typically after 7-28 days for results, depending on the participant's availability, ART supply and clinic schedules).

Management of viral load results at routine follow-up clinic visits

Viral load results will be managed by clinic staff in accordance with South African guidelines, which include guidance for management of both efavirenz and dolutegravir-based regimens, and do not advise routine HIV drug resistance testing.[20] Participants with a viral load ≥1000 copies/ml may meet criteria for viral failure (two consecutive viral loads ≥1000 copies/mL over 3 months apart) and will be considered for a switch to second-line ART. Switching to second-line ART and the choice of regimen will be at the clinician's discretion and will depend on the participant's first-line ART regimen, previous ART history, participant's preferences, perceived adherence and current guidelines.[20] Participants with a second viral load <1000 copies/mL will remain on first-line ART. In accordance with guidelines for the transition to dolutegravir, those receiving an NNRTI may have this switched to a dolutegravir-based first-line regimen.[13]

Data capture during routine clinic visits

Research staff will monitor participants' clinical records to capture visit dates, whether enhanced adherence counselling was conducted by a counsellor, nurse or doctor, medication prescriptions including ART, and laboratory results.

Study Exit Visit

The study exit visit will occur at 24 weeks (visit window 22-30 weeks), after which participants will continue receiving standard care at the Prince Cyril Zulu Clinic. At 24 weeks, any participant who has not attended the study exit visit will be called by the research team and asked to attend, irrespective of whether they have a routine clinic visit scheduled in the exit visit window.

Participant Reimbursement

We will reimburse study participants ZAR150 (approximately GBP8.00) after the enrolment visit and ZAR100 (GBP5.50) after the 24 week visit, to cover any additional time spent at the clinic and any inconvenience due to study participation.[21]

Extended follow up of routine data

For participants who provide additional consent, research staff may access routine medical records for up to five years after the study exit visit, in order to determine longer term retention in care, ART adherence and viral load results. For participants who are lost to follow up, and

For peer review only - http://bmjopen.bmj.com/site/about/guidelines.xhtml

who provided consent at enrolment, the participants mortality status may be checked on the South African National Population Register.

Additional laboratory specimens

Participants who provide additional consent will also have 10 mL of venous blood taken for storage and retrospective ART drug level and HIV drug resistance testing. Plasma will be stored at enrolment, during follow up alongside the repeat viral load test, and at study exit. In addition, at enrolment, 5 mL of urine will be taken for storage and ART drug level testing. We may use study samples to validate new point-of-care viral load, HIV drug resistance, or ART drug level assays. These results would not be used to guide clinical management.

Study outcomes

We aim to broadly estimate the effect of point-of-care viral load testing using the primary outcome of viral suppression < 50 copies/mL at 24 weeks after enrolment, measured on a laboratory reference assay (e.g. Alinity m HIV-1) in both arms. Feasibility outcomes include proportions of participants enrolled, followed-up and who had same-day viral load testing in the point-of-care arm (Table 2).

Data collection and management

The research team will capture study data using standardised electronic CRFs in REDCap, which is a password protected data management service stored on a secure server.[18] All data entry will undergo three stages of quality control including pre-programmed data validity checks in electronic CRFs, immediate source document review, and weekly quality reports generated using REDCap. To protect confidentiality, all study-specific documents, other than the signed consent, will refer to the participant using their study participant number rather than their name. All documents will be stored in a secure cabinet in the locked research office and will only be accessible by study staff and authorised personnel.

Statistical methods

At the conclusion of the study, we will assess the proportion of participants achieving study outcomes in each arm, with 95% confidence intervals calculated using the Wilson method. We will conduct exploratory analyses using Fisher's exact test to compare the proportions achieving binary study outcome measures in each arm, using an intention to treat analysis. As this is a feasibility study, these exploratory analyses will likely not be powered to test the hypotheses that there is a difference between the two arms for most outcome measures.

For peer review only - http://bmjopen.bmj.com/site/about/guidelines.xhtml

Primary outcome

We will calculate the proportion of participants in each arm achieving the binary primary outcome of viral suppression <50 copies/mL at 24 weeks (Table 2). Participants who are lost to follow up with no viral load result will be included in the analysis as not having achieved the primary outcome.

Secondary outcomes

We will calculate the proportion of potentially eligible patients with viraemia at the Prince Cyril Zulu clinic who are enrolled in the study (Table 3). The denominator will be the number of patients with viraemia during the study enrolment period, which will be calculated by clinic staff using NHLS high viral load reports. These reports are routinely used for monitoring and evaluation of clinic performance and include the number of first high viral loads ≥1000 copies/mL taken from the Prince Cyril Zulu Clinic each week. To assess study follow-up, we will calculate the proportion of participants who attend the study exit visit at 24 weeks after enrolment, overall and in each arm in case point-of-care testing influences retention. To assess implementation of point-of-care viral load testing, we will calculate the proportion of point-of-care viral load tests which are provided to participants on the same day, the proportion of viral loads ≥1000 copies/mL which resulted in same day enhanced adherence counselling, and the proportion of viral loads ≥1000 copies/mL resulting in appropriate same-day switch to second-line ART.

Tertiary Outcomes

In each arm we will assess the median number of days (and interquartile range) from enrolment to detection of viral failure, and from enrolment to appropriate switch to second-line ART (among participants with viral failure), and from enrolment to appropriate switching to dolutegravir (among participants on an NNRTI-based regimen). We will compare time to event outcomes using Cox proportional hazards. We will also calculate the proportion of participants with HIV drug resistance detected at 24 weeks after enrolment.

Sample size considerations

We conservatively estimate (based on our previous study)[9 10] that we could enrol approximately 100 participants in 6 months. If enrolment proceeds better than anticipated, and resources allow us to enrol more, this will provide more precise estimates of the study outcomes. If enrolment proceeds slower than expected, we will consider expanding to another clinic site to achieve a minimum of 80 participants. Therefore, while we anticipate 100 participants, the final number

For peer review only - http://bmjopen.bmj.com/site/about/guidelines.xhtml

294 may be between approximately 80-180 participants, and will be determined by the time and
295 resources available. We will use NHLS, Prince Cyril Zulu Clinic and study data to assess
296 outcomes, with estimated precision, in Table 3. Although not the primary aim of this feasibility
297 study, assuming 50% of SOC participants achieve the main outcome of viral load suppression
298 <50 copies/ml at 24 weeks, a sample size of 100 participants (50 per study arm) would provide
299 91% power to detect a +30% difference in the point-of-care testing arm, using a 2-sided alpha of
300 0.05.

301 **Quality assurance procedures**

302 Study documentation will be subject to internal quality audits by the Centre for the AIDS
303 Programme of Research (CAPRISA) Quality Assurance team, in accordance with CAPRISA
304 standard operating procedures.

305 **Qualitative sub-study**

306 We will undertake a qualitative sub-study within POwER to assess what changes in clinic
307 systems are required and what the views of staff are in implementing point-of-care viral load
308 testing, its potential impact on adherence and adherence counselling, and managing viraemia.
309 We will conduct semi-structured interviews and a focus group discussion with approximately 8-
310 10 staff during, and then again after, implementation of point-of-care viral load testing in the
311 study. Staff will include counsellors, phlebotomists, nurses, pharmacy staff, laboratory staff,
312 doctors and health service managers. Topic guides will be informed by Normalisation Process
313 Theory which aims to identify what is needed to 'normalise' use of a technology in a healthcare
314 system.[22 23] Discussions will be transcribed and thematically analysed using NVIVO software
315 (QSR International, Melbourne, Australia).

316 **Patient and public involvement**

317 Patients were not involved in the design of this study.

318 **ETHICS AND DISSEMINATION**

319 We will conduct the trial in accordance with the principles of the Declaration of Helsinki. The
320 eThekwini Municipality Health Unit Research Committee, the KwaZulu-Natal Provincial Health
321 Research Ethics Committee (KZ_202002_005), the University of KwaZulu-Natal Biomedical
322 Research Ethics Committee (BREC/00000836/2019) and the University of Oxford Tropical
323 Research Ethics Committee (OxTREC 66-19) have approved the study. We will present study
324 results to front-line clinical workers and programme managers in eThekwini Municipality and

For peer review only - http://bmjopen.bmj.com/site/about/guidelines.xhtml

KwaZulu-Natal Provincial Department of Health. We will also submit our findings to academic conferences and open-access, peer-reviewed journals.

Trial status

We registered POwER on the Pan African Clinical Trials Registry (PACTR202001785886049) on the 21st January 2020 and on the South African Clinical Trials (DOH-27-072020-6890) on the 17th July 2020. Enrolment started on 20th August 2020, under protocol version 2.0 dated 24th June 2020 (supplementary file).

DISCUSSION

Evidence-based interventions to improve the management of viraemia are needed to achieve global HIV treatment targets, prevent the development and spread of HIV drug resistance, and improve patient outcomes. In this paper, we outline a feasibility trial of point-of-care viral load testing among people with viraemia who are receiving first-line ART in South Africa. Results from this study will inform the design of future trials to assess the clinical effectiveness of point-of-care testing to manage viraemia.

Problems with management of viraemia

Several studies highlight gaps in the management of viraemia in LMICs, and the resulting negative impacts on treatment outcomes. First, addressing potential poor adherence, which may have caused viraemia, is challenging. A meta-analysis of interventions to improve adherence did not find any strategies that resulted in better viral suppression in LMICs,[24] while a more recent cluster-randomised trial of the South African National Adherence Guidelines found no difference in long-term viral re-suppression among patients with viraemia.[5] Second, follow up viral load testing is often poorly performed. In the South African trial, less than 20% of patients had a repeat viral load within the recommended 3 months to confirm viral failure.[5] Similar delays have been reported in a large analysis of South African laboratory data; among 260,323 patients with viraemia, the median time to a repeat viral load was 6.2 months, (interquartile range [IQR] 4.0-11.1). Third, among people with confirmed viral failure, a systematic review,[25] and more recent individual studies,[4 26-28] have found that only 30-60% are switched to second-line ART. Even among patients who are switched, this can take a further 3 to 12 months,[26-28] with delays of even a few months being associated with increased risk of opportunistic infections and mortality, particularly among people with low CD4 counts.[1 4 26-28] Taken together, these data highlight the need for interventions to reduce delays and improve the management of viraemia.

For peer review only - http://bmjopen.bmj.com/site/about/guidelines.xhtml

357 **Existing research regarding clinical effectiveness of point-of-care viral load testing**

358 We conducted the STREAM trial, which was the first randomised trial of point-of-care HIV viral
359 load testing for monitoring ART.[9 10] Overall, the STREAM intervention improved retention in care
360 and viral suppression by 14.3% compared to the standard-of-care laboratory viral load arm.
361 However, STREAM was designed to enrol stable patients; less than 5% of participants had
362 viraemia at enrolment, and only 16 participants developed viral failure (seven in the point-of-
363 care arm, nine in the standard care arm). Time to detection of viral failure was faster in the
364 intervention arm (55 days [IQR 55–57] vs 123 days [IQR 98–162]) but numbers with viraemia
365 were too small to determine whether point-of-care testing was effective at improving switching to
366 second line ART, or subsequent viral load outcomes. In STREAM, the research team provided
367 point-of-care testing and clinical care, so the observed benefits may also not be applicable to
368 routine public sector settings.

369 Several more trials of point-of-care viral load testing are in progress, but none focus on
370 management of viraemia. Studies in Zimbabwe[29 30], Kenya[31] and Haiti[32], are assessing point-of-
371 care viral load for monitoring ART amongst pregnant women, children and/or adolescents. A
372 study in Nigeria is enrolling adults at ART initiation and will report on the effect of point-of-care
373 viral load testing after 12 months[33], while a trial in South Africa[34] is enrolling adults on ART who
374 are due for their annual viral load test. One trial in Uganda is assessing a viral load intervention
375 package that includes 'near point-of-care testing' (at the clinic or a nearby clinic and therefore
376 results may not be provided on the same day), and is enrolling 'high-risk groups' including
377 patients with previous viraemia or no viral load in the past year[35].

378 **Implementation of point-of-care viral load testing**

379 Point-of-care diagnostics have been used widely in healthcare systems in many LMICs.[36] In HIV
380 programmes, some tests such as rapid, lateral flow assays for diagnosis of HIV, have been
381 evaluated, endorsed and incorporated into WHO guidelines and successfully adopted in many
382 settings.[37] However, other assays, such as more complex molecular polymerase chain reaction
383 technologies for tuberculosis, have remained as laboratory tests despite being marketed as
384 point-of-care assays.[36] Given that the Xpert HIV-1 VL and m-PIMA assays use similar platforms
385 and take over 1 hour for results to be available, they have also been criticized as not being
386 implementable as true point-of-care tests. A large systematic review of barriers to point-of-care
387 HIV diagnostic implementation in LMICs found that in 132 studies, integration of the point-of-
388 care test into clinical work flows was the most commonly identified challenge to test utilization.[38]
389 More recently, studies have reported implementation of qualitative point-of-care viral load

For peer review only - http://bmjopen.bmj.com/site/about/guidelines.xhtml

assays for early infant diagnosis of HIV[39], and monitoring of ART in maternity wards in South Africa[40], and de-centralised ART clinics in Malawi[41]. While these studies have reported quantitative outcomes related to point-of-care viral load use, there is little qualitative work using theory-based approaches to evaluate what is required for successful implementation.

Strengths and limitations

A major strength of this study is the focus on patients with viraemia, who are a priority population at high risk of increased morbidity and mortality, onwards HIV transmission, and development of HIV drug resistance.[1] Interventions to improve viral suppression amongst this group have been prioritized by the WHO and align with several objectives in the Global Action Plan on HIV Drug Resistance[42]. While our study may be under-powered to detect a significant effect of point-of-care viral load testing on viral re-suppression, we will determine the feasibility of conducting larger studies to answer this question in this setting. Our use of Normalisation Process Theory will allow a rigorous assessment of what is needed to 'normalise' point-of-care testing in the South African healthcare system.

A limitation of this study is the focus on 'supply side' implementation of point-of-care viral load testing. Receiving results on the same day could also increase patient understanding and motivate adherence, but evaluating this from the patient perspective is beyond the scope of the study. Furthermore, findings from this large urban, clinic may not be applicable to other settings, including smaller clinics in rural settings where advantages of point-of-care testing over laboratory-based testing may differ. Our future trial may be best designed as a cluster-randomised trial, with individual clinics randomised to receive the point-of-care testing intervention. While POwER is an individually randomized study, estimates of the effect of point-of-care testing will remain valuable for design of potential cluster randomized or individually randomized future studies.

Coinciding with the rollout of dolutegravir is both a strength and a weakness of our study. We will enrol patients on either NNRTI- or dolutegravir-based ART, therefore allowing estimates of the effect of point-of-care testing in either group. For patients enrolled on NNRTIs, this also presents an opportunity to assess whether point-of-care viral load testing can assist the transition to dolutegravir-based regimens. For patients enrolled on dolutegravir, a potential limitation is that point-of-care viral load testing will not be used to expedite a switch to second line, although it may still improve enhanced adherence counselling and allow more efficient care that can help ensure retention and virological suppression. Lastly, we will be able to assess

For peer review only - http://bmjopen.bmj.com/site/about/guidelines.xhtml

early viral suppression outcomes and the frequency of HIV drug resistance among people with viraemia on dolutegravir. This is a pressing research question for global policy makers, because it determines the need for HIV drug resistance testing and second-line ART after introduction of a dolutegravir-based first-line in LMICs.

In summary, we aim to provide evidence as to whether a trial of point-of-care viral load testing to manage viraemia is feasible, and how testing may be implemented in South African clinics. This work will contribute to the development of interventions to improve the management of viraemia amongst people receiving ART, thereby improving health and leading to longer and better lives, as well as reducing the spread of HIV and preventing drug resistance.

For peer review only

For peer review only - http://bmjopen.bmj.com/site/about/guidelines.xhtml

LIST OF ABBREVIATIONS

ART – antiretroviral therapy

UNAIDS - Joint United Nations Programme on HIV/AIDS

LMIC – low- and middle-income countries

WHO – World Health Organization

DECLARATIONS

Competing interests

Cepheid and Abbott have provided point-of-care viral load assays at no cost for use at the study site. The authors have no other competing interests to declare.

Funding

This work is supported by grants from the Wellcome Trust PhD Programme for Primary Care Clinicians (216421/Z/19/Z), the University of Oxford's Research England QR Global Challenges Research Fund (0007365) and the Africa Oxford Initiative (AfiOx-119). The University of Oxford is the study sponsor. The funders and sponsor have no role in study design, manuscript submission, or collection, management, analysis or interpretation of study data.

Author contributions

JD and NG conceived the study. YS, HN, RL, FS, EB, PM, NS, LL, STC, PKD, GH and CB contributed to study design and implementation. STC contributed to design of the qualitative sub-study. JD wrote the first draft of the manuscript. All authors critically reviewed and edited the manuscript and consented to final publication.

Acknowledgements

The authors would like to thank all participants in the study and acknowledge the work and support of staff at the Prince Cyril Zulu Clinic, eThekwini Municipality, CAPRISA and the National Health Laboratory Services at Addington and Inkosi Albert Luthuli Hospitals.

Data sharing statement

Bona fide researchers will be able to request access to anonymised trial data by contacting the corresponding author.

For peer review only

REFERENCES

1. Murphy RA, Court R, Maartens G, et al. Second-line antiretroviral therapy in sub-Saharan Africa: It's time to mind the gaps. *AIDS Research and Human Retroviruses* 2017;33(12):1181-84. doi: 10.1089/aid.2017.0134
2. Labhardt ND, Ringera I, Lejone TI, et al. When patients fail UNAIDS' last 90 - the "failure cascade" beyond 90-90-90 in rural Lesotho, Southern Africa: a prospective cohort study. *Journal of the International AIDS Society* 2017;20(1):1-10. doi: 10.7448/IAS.20.1.21803
3. Etoori D, Ciglenecki I, Ndlangamandla M, et al. Successes and challenges in optimizing the viral load cascade to improve antiretroviral therapy adherence and rationalize second-line switches in Swaziland. *Journal of the International AIDS Society* 2018;21(10):e25194-e94. doi: 10.1002/jia2.25194
4. Hermans LE, Carmona S, Nijhuis M, et al. Virological suppression and clinical management in response to viremia in South African HIV treatment program: A multicenter cohort study. *PLoS Med* 2020;17(2):e1003037. doi: 10.1371/journal.pmed.1003037 [published Online First: 2020/02/26]
5. Fox MP, Pascoe SJS, Huber AN, et al. Effectiveness of interventions for unstable patients on antiretroviral therapy in South Africa: results of a cluster-randomised evaluation. *Tropical Medicine & International Health* 2018;23(12):1314-25. doi: 10.1111/tmi.13152
6. Shubber Z, Mills EJ, Nachega JB, et al. Patient-Reported Barriers to Adherence to Antiretroviral Therapy: A Systematic Review and Meta-Analysis. *PLOS Medicine* 2016;13(11):e1002183-e83. doi: 10.1371/journal.pmed.1002183
7. World Health O. WHO prequalification of in vitro diagnostics. Product : Xpert® HIV-1 Viral Load with GeneXpert® Dx, GeneXpert® Infinity- 48, GeneXpert® Infinity-48s and GeneXpert® Infinity-80, 2017.
8. World Health Organization. WHO Prequalification of In Vitro Diagnostics Public Report. Product: m-PIMA HIV-1/2 VL, 2019.
9. Dorward J, Garrett N, Quame-Amaglo J, et al. Protocol for a randomised controlled implementation trial of point-of-care viral load testing and task shifting: the Simplifying HIV TREAtment and Monitoring (STREAM) study. *BMJ Open* 2017;7(9):e017507-e07. doi: 10.1136/bmjopen-2017-017507
10. Drain PK, Dorward J, Violette LR, et al. Point-of-care HIV viral load testing combined with task shifting to improve treatment outcomes (STREAM): findings from an open-label, non-inferiority, randomised controlled trial. *Lancet HIV* 2020;7(4):e229-e37. doi: 10.1016/S2352-3018(19)30402-3 [published Online First: 2020/02/28]
11. Mody A, Geng EH. Conceptualising implementation strategies in HIV research. *The Lancet HIV* 2020;7(6):e382. doi: 10.1016/S2352-3018(20)30142-9
12. Eldridge SM, Lancaster GA, Campbell MJ, et al. Defining Feasibility and Pilot Studies in Preparation for Randomised Controlled Trials: Development of a Conceptual Framework. *PloS one* 2016;11(3):e0150205-e05. doi: 10.1371/journal.pone.0150205
13. South Africa National Department of Health. National Consolidated Guidelines for the Management of HIV in Adults, Adolescents, Children and Infants and Prevention of Mother-to-Child Transmission. Pretoria, South Africa: South African National Department of Health, 2020.
14. Dorward J, Lessells R, Drain PK, et al. Dolutegravir for first-line antiretroviral therapy in low-income and middle-income countries: uncertainties and opportunities for implementation and research. *Lancet HIV* 2018;5(7):e400-e04. doi: 10.1016/S2352-3018(18)30093-6 [published Online First: 2018/06/10]

For peer review only - http://bmjopen.bmj.com/site/about/guidelines.xhtml

15. Vitoria M, Hill A, Ford N, et al. The transition to dolutegravir and other new antiretrovirals in low-income and middle-income countries: what are the issues? *AIDS* 2018;32(12):1551-61. doi: 10.1097/QAD.0000000000001845 [published Online First: 2018/05/11]
16. Hamers RL, Rinke de Wit TF, Holmes CB. HIV drug resistance in low-income and middle-income countries. *The Lancet HIV* 2018;5(10):e588-e96. doi: 10.1016/S2352-3018(18)30173-5
17. World Health O. Consolidated guidelines on the use of antiretroviral drugs for treating and preventing HIV infection: recommendations for a public health approach – 2nd ed. Geneva, Switzerland, 2016:427-27.
18. Harris PA, Taylor R, Thielke R, et al. Research electronic data capture (REDCap)--a metadata-driven methodology and workflow process for providing translational research informatics support. *J Biomed Inform* 2009;42(2):377-81. doi: 10.1016/j.jbi.2008.08.010 [published Online First: 2008/10/22]
19. South Africa National Department of Health. Minimum package of interventions to support linkage to care, adherence and retention in care: South African National Department of Health, 2020.
20. The South African National Department of H. 2019 ART Clinical Guidelines for the management of HIV in Adults, Pregnancy, Adolescents, Children, Infants and Neonates. Pretoria, South Africa, 2019.
21. National Health Research Ethics C. Payment of trial participants in South Africa: Ethical considerations for Research Ethics Committees: South African Department of Health, 2012.
22. May C, Finch T. Implementing, embedding, and integrating practices: An outline of normalization process theory. *Sociology* 2009;43(3):535-54. doi: 10.1177/0038038509103208
23. May CR, Mair F, Finch T, et al. Development of a theory of implementation and integration: Normalization Process Theory. *Implementation Science* 2009;4(1):29-29. doi: 10.1186/1748-5908-4-29
24. Kanters S, Park JJH, Chan K, et al. Interventions to improve adherence to antiretroviral therapy: a systematic review and network meta-analysis. *The Lancet HIV* 2017;4(1):e31-e40. doi: 10.1016/S2352-3018(16)30206-5
25. Ford N, Orrell C, Shubber Z, et al. HIV viral resuppression following an elevated viral load: a systematic review and meta-analysis. *Journal of the International AIDS Society* 2019;22(11):1-6. doi: 10.1002/jia2.25415
26. Rohr JK, Ive P, Robert Horsburgh C, et al. Marginal structural models to assess delays in second-line HIV treatment initiation in South Africa. *PLoS ONE* 2016;11(8):1-11. doi: 10.1371/journal.pone.0161469
27. Bell-Gorrod H, Fox MP, Boulle A, et al. The Impact of Delayed Switch to Second-Line Antiretroviral Therapy on Mortality, Depending on Definition of Failure Time and CD4 Count at Failure. *Am J Epidemiol* 2020;189(8):811-19. doi: 10.1093/aje/kwaa049 [published Online First: 2020/03/29]
28. Bell-Gorrod H, Court R, Schomaker M, et al. Increased Mortality With Delayed and Missed Switch to Second-Line Antiretroviral Therapy in South Africa. *J Acquir Immune Defic Syndr* 2020;84(1):107-13. doi: 10.1097/QAI.0000000000002313 [published Online First: 2020/02/08]
29. Munyati S. NCT03986099 Community Based Antiretroviral Therapy (CBART) Among Children on Chronic ART: ClinicalTrials.gov; 2019 [28 August 2020]. Available from: https://clinicaltrials.gov/ct2/show/NCT03986099 accessed 28 August 2020.
30. Clinton Health Access Initiative. NCT04048629 Impact of Point-of-care (POC) Viral Load (VL) Testing During Pregnancy in Zimbabwe ClinicalTrials.gov2019 [28 August 2020].

For peer review only - http://bmjopen.bmj.com/site/about/guidelines.xhtml

Available from: https://clinicaltrials.gov/ct2/show/NCT04048629 accessed 28 August 2020.

31. Patel R. NCT03820323 Optimizing Viral Load Suppression in Kenyan Children on Antiretroviral Therapy (Opt4Kids) ClinicalTrials.gov2019 [28 August 2020]. Available from: https://clinicaltrials.gov/ct2/show/NCT03820323 accessed 28 August 2020.
32. Reif LK, Belizaire ME, Seo G, et al. Point-of-care viral load testing among adolescents and youth living with HIV in Haiti: a protocol for a randomised trial to evaluate implementation and effect. *BMJ Open* 2020;10(8):e036147. doi: 10.1136/bmjopen-2019-036147
33. Meloni ST, Agbaji O, Chang CA, et al. The role of point-of-care viral load monitoring in achieving the target of 90 % suppression in HIV-infected patients in Nigeria : study protocol for a randomized controlled trial. 2019:1-11.
34. Theron G. NCT03187964: Xpert Ultra and Xpert HIV-VL in People Living With HIV (UltraHIV), 2017.
35. Jain V. NCT03553693: Rapid HIV Viral Load Monitoring in High Risk Patients In Uganda (RAPID-VL), 2018.
36. Jani IV, Peter TF. How Point-of-Care Testing Could Drive Innovation in Global Health. *The New England journal of medicine* 2013;368(24):2319-24. doi: 10.1056/NEJMsb1214197
37. Drain PK, Rousseau C. Point-of-care diagnostics: Extending the laboratory network to reach the last mile. *Current Opinion in HIV and AIDS* 2017;12(2):175-81. doi: 10.1097/COH.0000000000000351
38. Pai NP, Wilkinson S, Deli-Houssein R, et al. Barriers to Implementation of Rapid and Point-of-Care Tests for Human Immunodeficiency Virus Infection. *Point of Care* 2015;14(3):81-87. doi: 10.1097/POC.0000000000000056
39. Bianchi F, Cohn J, Sacks E, et al. Evaluation of a routine point-of-care intervention for early infant diagnosis of HIV: an observational study in eight African countries. *The Lancet HIV* 2019:1-9. doi: 10.1016/S2352-3018(19)30033-5
40. Kufa T, Mazanderani AH, Sherman GG, et al. Point-of-care HIV maternal viral load and early infant diagnosis testing around time of delivery at tertiary obstetric units in South Africa: a prospective study of coverage, results return and turn-around times. *J Int AIDS Soc* 2020;23(4):e25487. doi: 10.1002/jia2.25487 [published Online First: 2020/04/25]
41. Nicholas S, Poulet E, Wolters L, et al. Point-of-care viral load monitoring: outcomes from a decentralized programme in Malawi. *Journal of the International AIDS Society* 2019;22(8):1-9. doi: 10.1002/jia2.25387
42. World Health O. Global Action Plan for HIV Drug Resistance 2016-2021. Geneva, Switzerland, 2017.

For peer review only

For peer review only - http://bmjopen.bmj.com/site/about/guidelines.xhtml

602 **TABLES**

603 **Table 1: Schedule of evaluation in the POwER study**

	**Enrol**	**Routine clinic visit follow-up[a]**					**Study exit**
**Weeks in study**	**0**	**4**	**8**	**12**	**16**	**20**	**24[b]**
Informed consent	X						
Locator information	X						
Demographics	X						
Medical history	X						
Vital signs	X	X	X	X	X	X	X
Physical examination	X						
Urine pregnancy test	X						
Eligibility screen	X						
Enhanced adherence counselling	X	X	X	X	X[c]	X[c]	X[c]
Randomization	X						
Routine Department of Health testing [d]	X						
POC viral load (intervention arm)				X[e]			
Laboratory viral load (standard of care arm)				X[e]			
Reference viral load outcome measure							X
Evaluation of new POC assays[f]	X			X			X
Stored blood & HIV drug resistance testing[g]	X			X			X
Stored urine for ART drug level testing	X						

604 [a] Scheduled at participants and healthcare worker's discretion but typically every 28 days
605 [b] Study exit visit window 22-30 weeks
606 [c] Ongoing enhanced adherence counselling for participants with viral load ≥50 copies/mL

For peer review only - http://bmjopen.bmj.com/site/about/guidelines.xhtml

[d] CD4 count testing (4 mL EDTA), hepatitis B surface antigen (5 mL SST), creatinine (5 mL SST) and haemoglobin (4 mL EDTA)

[e] Repeat viral load testing recommended at 12 weeks but may be done any time during follow up at the attending clinician's discretion

[f] Maximum of 8 mL venous blood and/or a finger-prick capillary blood sample

[g] Retrospective drug resistance testing for viraemic participants at enrolment, follow up or study exit (10 mL EDTA)

ART = antiretroviral therapy, POC = point-of-care

Table 2: Study objectives and outcomes in the POwER study

**Objectives**	**Outcome Measures**	**Timepoint(s) of evaluation of this outcome measure (if applicable)**
**Primary Objective**<br>To estimate the effect of implementing point-of-care viral load testing versus standard laboratory viral load testing on the proportion of HIV positive participants with viraemia who subsequently achieve viral resuppression	The proportion of participants in each arm with viral load <50 copies/mL measured on laboratory reference assay	24 weeks after enrolment
**Secondary Objectives**<br>To assess whether it is feasible to perform a randomised implementation trial of point-of-care viral load testing to manage viraemia in a routine South African clinic, by determining:		
a) What proportion of people with HIV viraemia is feasible to enrol?	a) Proportion of patients with viraemia at the study clinic who are successfully enrolled in the study	a) During study enrolment period
b) What proportion of those enrolled is feasible to follow up?	b) Proportion of enrolled participants who attend the study exit visit overall	b) 24 weeks after enrolment

For peer review only

	and in each arm	
c) What proportion of point-of-care viral load results are received and acted on in the same day?	c1) The proportion of point-of-care viral load tests that are communicated to participants on the same day<br>AND<br>c2) The proportion of point-of-care viral load tests ≥1000 copies/mL that result in same day enhanced adherence counselling<br>AND<br>c3) The proportion of point-of-care viral load tests ≥1000 copies/mL that result in same day switch to second-line ART	c) 12 weeks after enrolment when point-of-care testing is performed
**Tertiary Objectives**<br>To estimate the effect of point-of-care viral load testing versus standard laboratory viral load testing on:		
a) Time to detection of viral failure (consecutive viral loads ≥1000 copies/mL) in each arm	a) Days from enrolment to availability of viral load result ≥1000 copies/mL	a) By 24 weeks after enrolment
b) Time to switch to second-line ART in each arm	b) Days from enrolment to appropriate switching to second-line ART, amongst participants with viral failure	b) By 24 weeks after enrolment
c) Appropriate switching to dolutegravir in each arm	c) Time to appropriate switching to dolutegravir in each arm	c) By 24 weeks after enrolment
d) HIV drug resistance in each arm	d) The proportion of participants in each arm with HIV drug resistance	d) 24 weeks after enrolment
**Qualitative Study Objectives:**<br>What changes in clinic systems are required and what are the views and experiences of staff in	Staff perspectives regarding implementation of point-of-care viral load testing in a routine clinic	During enrolment when point-of-care testing is

For peer review only - http://bmjopen.bmj.com/site/about/guidelines.xhtml

implementing point-of-care viral load testing to manage viraemia?		being implemented, and after study conclusion

Table 3: Study & process evaluation outcomes with precision estimates

Outcome	Estimated n/N, %	95% CI (Wilson)
Percentage with viral load <50 copies/ml at 24 weeks in POC arm	35/50, 70.0	56.2-80.9
Percentage with viral load <50 copies/ml at 24 weeks in SOC arm	25/50, 50.0	36.6-63.4
Percentage of viraemic patients successfully enrolled in study	100/240*, 41.7	35.6-48.0
Percentage of those enrolled who are retained at 24 weeks	90/100, 90.0	82.6-94.5
Percentage in POC arm with same-day viral load testing	40/50, 79.5	70.0-88.8

*Assuming 240 viraemic patients during enrolment period, many of whom will not be eligible due to being on second-line ART, having had previous high viral loads, already having received enhanced adherence counselling or pregnancy. POC = point-of-care, SOC = standard-of-care

For peer review only

623 **FIGURE LEGENDS**

624 Figure 1: Consort diagram of the POwER study

625 ART = antiretroviral therapy, VL = viral load

For peer review only

For peer review only - http://bmjopen.bmj.com/site/about/guidelines.xhtml

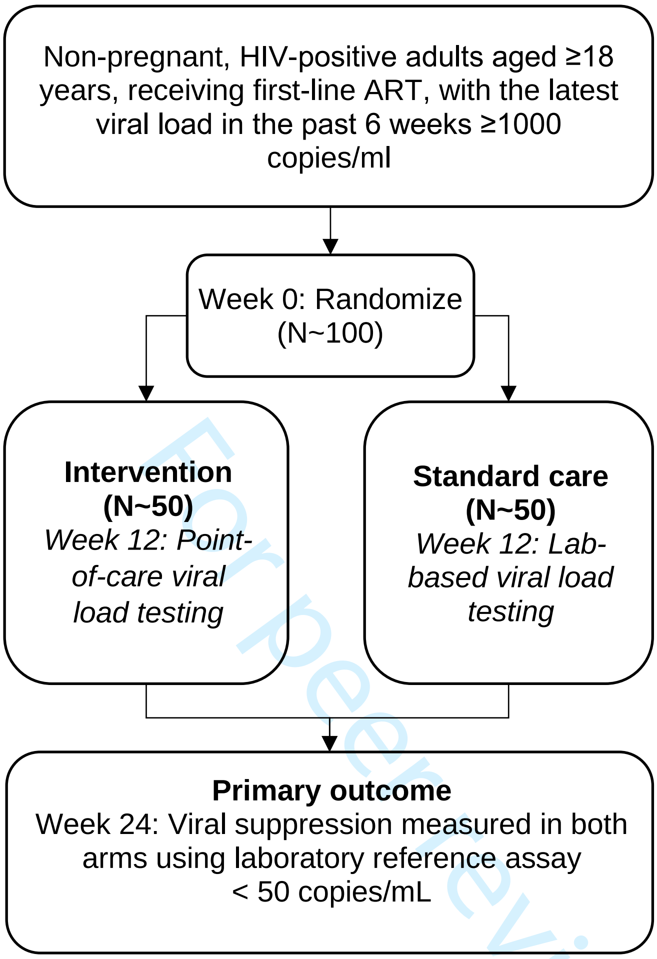

For peer review only

Study Title: POwER: Point Of care viral load testing to Enhance Re-suppression

Internal Reference Number / Short title: POwER

OxTREC Ref: 66-19

Date and Version No: Version 2.0 24th June 2020

Chief Investigator: Dr Jienchi Dorward, Nuffield Department of Primary Care Health Sciences, University of Oxford

Dr Nigel Garrett, Centre for the AIDS Programme of Research in South Africa, University of KwaZulu-Natal

Investigators: Prof Chris Butler, Nuffield Department of Primary Care Health Sciences, University of Oxford

Mrs Hope Ngobese, eThekwini Municipality Health Unit, eThekwini Municipality

Dr Yukteshwar Sookrajh, eThekwini Municipality Health Unit, eThekwini Municipality

Prof Gail Hayward, Nuffield Department of Primary Care Health Sciences, University of Oxford

Dr Paul Drain, Department of Global Health, University of Washington

Dr Richard Lessells, KwaZulu-Natal Research Innovation & Sequencing Platform, University of KwaZulu-Natal

Sponsor: University of Oxford

Funder: Internal Research England Global Challenges Research Fund, Wellcome Trust PhD Programme for Primary Care Clinicians, Africa Oxford Initiative.

Chief Investigator Signature:

Conflict of interest statement

We have no conflicts of interest to declare.

Confidentiality Statement

This document contains confidential information that must not be disclosed to anyone other than the authorised individuals from the University of Oxford, the Investigator Team and members of the Oxford Tropical Research Ethics Committee (OxTREC), unless authorised to do so.

For peer review only

TABLE OF CONTENTS

1. SYNOPSIS 4
2. ABBREVIATIONS 5
3. BACKGROUND AND RATIONALE 6
4. OBJECTIVES AND OUTCOME MEASURES 7
5. STUDY DESIGN 8
6. STUDY SETTING 9
7. PARTICIPANT IDENTIFICATION AND RECRUITMENT 9
7.1. Study Participants 9
7.2. Inclusion Criteria 9
7.3. Exclusion Criteria 10
8. STUDY PROCEDURES 10
8.1. Recruitment 10
8.2. Informed Consent 10
8.3. Screening and Eligibility Assessment 10
8.4. Randomisation, blinding and code-breaking 11
8.5. Baseline Assessments 11
8.5.1. Baseline demographics and medical history 11
8.5.2. Laboratory testing 11
8.6. Follow-up Routine Clinic Visits 11
8.6.1. Viral load testing at follow-up routine clinic visits 11
8.6.2. Management of viral load results at follow-up routine clinic visits 12
8.6.3. Data captures at routine clinic visits 12
8.7. Study Exit Visit 12
8.8. Extended follow up of routine data 13
8.9. Sample Handling 13
8.9.1. Point-of-care testing 13
8.9.2. Standard laboratory viral load testing 13
8.9.3. Other standard laboratory tests 13
8.9.4. Storage samples and HIV drug resistance testing 13
8.9.5. Potential evaluation of newer point-of-care assays 14
8.10. Discontinuation/Withdrawal of Participants from Study 14
8.11. Definition of End of Study 14

For peer review only

9. SAFETY REPORTING 14
10. STATISTICS AND ANALYSIS 14
10.1. Description of Statistical Methods 14
10.2. Analysis of Outcome Measures 15
10.2.1. Primary outcome 15
10.2.2. Secondary outcomes 15
10.2.3. Tertiary Outcomes 15
10.3. The Number of Participants 15
11. DATA MANAGEMENT 16
11.1. Access to Data 16
11.2. Data Handling and Record Keeping 16
12. QUALITATIVE STUDY 16
12.1. Background 16
12.2. Aim 16
12.3. Study design 17
12.4. Participants 17
12.5. Procedures 17
12.5.1. Informed consent 17
12.5.2. Interviews and focus groups 17
12.5.3. Analysis 17
13. QUALITY CONTROL AND QUALITY ASSURANCE PROCEDURES 17
14. ETHICAL AND REGULATORY CONSIDERATIONS 18
14.1. Declaration of Helsinki 18
14.2. Guidelines for Good Clinical Practice 18
14.3. Approvals 18
14.4. Participant Confidentiality 18
14.5. Expenses and Benefits 19
14.6. Reporting 19
14.7. Other Ethical Considerations 19
15. FINANCE AND INSURANCE 19
15.1. Funding 19
15.2. Insurance 20
16. PUBLICATION POLICY 20
17. REFERENCES 21

For peer review only

18. APPENDIX A: STUDY FLOW CHART 23
19. APPENDIX B: SCHEDULE OF STUDY PROCEDURES 24
20. APPENDIX C: SOUTH AFRICAN GUIDELINES FOR MANAGEMENT OF VIRAEMIA 25
21. APPENDIX D: SOUTH AFRICAN GUIDELINES FOR CHOICE OF SECOND-LINE ART 26
22. APPENDIX E: AMENDMENT HISTORY 26

1. SYNOPSIS

**Study Title**	POwER: Point Of care viral load testing to Enhance Re-suppression	
**Internal ref. no.**	POwER	
**Study Design**	This will be a single-site, open-label, individually randomized feasibility study	
**Study Participants**	HIV-positive adults aged ≥18 years, receiving first-line antiretroviral therapy (ART), with latest viral load in the past 6 weeks ≥1000 copies/ml, at the Prince Cyril Zulu Clinic in Durban, South Africa.	
**Planned Sample Size**	Approximately 100	
**Planned Study Period**	Two years	
	**Objectives**	**Outcome Measures**
**Primary**	To estimate the effect of implementing point-of-care viral load testing versus standard laboratory viral load testing on the proportion of HIV positive participants with viraemia who subsequently achieve viral resuppression	The proportion of participants in each arm with viral load <50 copies/mL after 24 weeks
**Secondary**	To assess whether it is feasible to perform a randomised implementation trial of point-of-care viral load testing to manage viraemia in a routine South African clinic, by determining:	
	a) What proportion of people with HIV viraemia is feasible to enrol?	a) Proportion of patients with viraemia at the study clinic who are enrolled in the study
	b) What proportion of those enrolled is feasible to follow up?	b) Proportion of enrolled participants who attend the 24 week study exit visit
	c) What proportion of point-of-care viral load results are received and acted on in the same day?	c) The proportion of point-of-care viral load tests that are communicated to participants on the same day, and the proportion resulting in same day enhanced adherence counselling and same day switch to second-line ART

For peer review only

**Tertiary Objectives**	To estimate the effect of point-of-care viral load testing versus standard laboratory viral load testing on:	
	a) Time to detection of viral failure (consecutive viral loads ≥1000 copies/mL) in each arm	a) Days from enrolment to availability of viral load result ≥1000 copies/mL
	b) Time to switch to second-line ART in each arm	b) Days from enrolment to appropriate switching to second-line ART, amongst participants with viral failure
	c) Dependent on dolutegravir rollout during the study period: Time to appropriate switching to dolutegravir in each arm	c) Days from enrolment to appropriate switching to dolutegravir-based ART according to South African guidelines
	d) HIV drug resistance in each arm	d) The proportion of participants in each arm with HIV drug resistance at 24 weeks
**Qualitative evaluation**	What changes in clinic systems are required and what are the views and experiences of staff in implementing point-of-care viral load testing to manage viraemia?	Staff perspectives regarding implementation of point-of-care viral load testing to manage viraemia

2. ABBREVIATIONS

BREC	Biomedical Research Ethics Committee
CAPRISA	Centre for the AIDS Programme of Research in South Africa
CI	Chief Investigator
CRF	Case Report Form
eGFR	Estimated glomerular filtration rate
GCP	Good Clinical Practice
HIV	Human Immunodeficiency Virus
ICF	Informed Consent Form
LMIC	Low- and middle-income country
MDRD	Modification of Diet in Renal Disease
NHLS	National Health Laboratory Service
OxTREC	Oxford Tropical Research Ethics Committee
PI	Principal Investigator
PIS	Participant Information Sheet
SOP	Standard Operating Procedure

For peer review only

WHO	World Health Organization

3. BACKGROUND AND RATIONALE

Initiating antiretroviral therapy (ART) to achieve viral load suppression amongst all people with HIV is crucial to achieve the UNAIDS target of ending AIDS by 2030. However, globally, 20% of people with HIV on ART have viraemia ≥1000 copies/mL, due to poor adherence and/or HIV drug resistance.[1] For these people, current World Health Organisation (WHO) guidelines recommend enhanced adherence counselling, with a repeat viral load within three months. If viraemia persists, HIV drug resistance is assumed, and second-line ART is recommended.[2,3] However, in low- and middle-income countries (LMICs), the majority of ART is provided in primary care, where there is limited laboratory viral load capacity, weak clinic systems to act on viral load results and a paucity of evidence based interventions to improve adherence.[4–9] Poor adherence can be due to complex social issues (e.g. sexual violence, alcohol use, migrant labour and long distances to clinics).[10] Therefore, multiple visits for blood draw, result review and repeated enhanced adherence counselling confers an additional burden for this vulnerable population. Laboratory viral load results are often not reviewed until the next routine clinic visit after several months, by which time adherence problems may have worsened.[4–9] Overall, these health system and individual factors result in ongoing viraemia, with associated increases in morbidity, mortality, onward HIV transmission and development and spread of HIV drug resistance.[4]

Point-of-care viral load testing could overcome some of these challenges by allowing a) immediate enhanced adherence counselling to address contemporary adherence b) same-day switching to second-line ART, and c) more efficient, person-centred care by reducing the burden of clinic visits for review of blood results; thereby leading to faster viral load re-suppression. WHO has approved the Xpert® HIV-1 VL (Cepheid, Sunnyvale, USA)[11] and the m-PIMA HIV-1/2 VL (Abbott, Chicago, USA)[12] as quantitative point-of-care HIV viral load assays for use in LMICs. The Xpert HIV-1 VL has been validated in multiple settings, including the proposed clinical site,[13] with good correlation and sensitivity of 96.5% and specificity of 96.6% to detect viraemia at a threshold of 1000 copies/mL.[14] This assay uses the GeneXpert platform which is widely available in LMICs for tuberculosis diagnostics. We performed the first pilot randomized controlled trial[15] of the Xpert HIV-1 VL, and demonstrated that in a research setting, point-of-care viral load testing helped to rapidly triage people with viral load suppression into more efficient care pathways. We found a 14% increase in retention in care and viral load suppression in the point-of-care testing arm compared to standard laboratory viral load testing.[15] However, this study **excluded clinically unstable patients** (only 5% had viraemia at enrolment). Therefore, we have inadequate data on the effect of point-of-care viral load testing amongst people with viraemia who are a vulnerable, priority population and could be difficult to enrol and follow up in trials. Furthermore, implementing point-of-care viral load testing requires multiple steps including assay maintenance, quality assurance and reorganisation of clinic flow, to ensure the result is used to provide immediate enhanced adherence counselling or switching of ART regimen in the same clinic visit, by the appropriate healthcare worker. In STREAM, research staff provided point-of-care viral load testing and clinical care, meaning that we do not know if implementation is feasible in routine primary care services. Therefore, we aim to test whether implementing point-of-care viral load testing to improve the management of viraemia is feasible, and whether the effect of point-of-care viral load testing on management of viraemia can be tested in a trial.

Lastly, many LMICs are now replacing current non-nucleoside reverse transcriptase inhibitors (NNRTIs)

For peer review only - http://bmjopen.bmj.com/site/about/guidelines.xhtml

with the new drug dolutegravir for first-line ART.[16] Dolutegravir is an integrase strand transfer inhibitor that, in high income settings, has been found to have better tolerability, efficacy, and durability than current NNRTI regimens. In South Africa, the rollout of dolutegravir is expected in late 2019/early 2020. Patients currently on NNRTI based first-line ART will require viral load testing to guide switching to dolutegravir-based first-line or second-line ART.[17] Our implementation study will likely coincide with the dolutegravir rollout, and presents an opportunity to assess whether point-of-care viral load testing can assist with the viral load informed transition to this newer regimen.

4. OBJECTIVES AND OUTCOME MEASURES

For peer review only

Objectives	Outcome Measures	Timepoint(s) of evaluation of this outcome measure (if applicable)
**Primary Objective**<br>To estimate the effect of implementing point-of-care viral load testing versus standard laboratory viral load testing on the proportion of HIV positive participants with viraemia who subsequently achieve viral resuppression	The proportion of participants in each arm with viral load <50 copies/mL measured on laboratory reference assay	24 weeks after enrolment
**Secondary Objectives**<br>To assess whether it is feasible to perform a randomised implementation trial of point-of-care viral load testing to manage viraemia in a routine South African clinic, by determining:		
a) What proportion of people with HIV viraemia is feasible to enrol?	a) Proportion of patients with viraemia at the study clinic who are successfully enrolled in the study	a) During study enrolment period
b) What proportion of those enrolled is feasible to follow up?	b) Proportion of enrolled participants who attend the study exit visit	b) 24 weeks after enrolment
c) What proportion of point-of-care viral load results are received and acted on in the same day?	c1) The proportion of point-of-care viral load tests that are communicated to participants on the same day<br>AND<br>c2) The proportion of point-of-care viral load tests ≥1000 copies/mL that result in same day enhanced adherence counselling<br>AND	c) 12 weeks after enrolment when point-of-care testing is performed

	c3) The proportion of point-of-care viral load tests ≥1000 copies/mL that result in same day switch to second-line ART	
**Tertiary Objectives**<br>To estimate the effect of point-of-care viral load testing versus standard laboratory viral load testing on:		
a) Time to detection of viral failure (consecutive viral loads ≥1000 copies/mL) in each arm	a) Days from enrolment to availability of viral load result ≥1000 copies/mL	a) By 24 weeks after enrolment
b) Time to switch to second-line ART in each arm	b) Days from enrolment to appropriate switching to second-line ART, amongst participants with viral failure	b) By 24 weeks after enrolment
c) Dependent on dolutegravir rollout during the study period: Appropriate switching to dolutegravir in each arm	c) The proportion of participants in each arm appropriately switched to dolutegravir-based ART according to South African guidelines	c) 24 weeks after enrolment
d) HIV drug resistance in each arm	d) The proportion of participants in each arm with HIV drug resistance	d) 24 weeks after enrolment
**Qualitative Study Objectives:**<br>What changes in clinic systems are required and what are the views and experiences of staff in implementing point-of-care viral load testing to manage viraemia?	Staff perspectives regarding implementation of point-of-care viral load testing in a routine clinic	During enrolment when point-of-care testing is being implemented, and after study conclusion

5. STUDY DESIGN

This will be a single-site, open-label, individually randomized feasibility study amongst people with recent HIV viraemia, comparing point-of-care viral load testing with standard laboratory viral load testing. We will also assess implementation of point-of-care viral load testing using process evaluation data, interviews and focus groups.

Eligible people will be HIV-positive, aged ≥18 years, receiving first-line ART, with latest viral load in the past 6 weeks ≥1000 copies/ml, and who have not received enhanced adherence counselling. Participants who provide written consent will be screened, enrolled and randomized in a 1:1 ratio to point-of-care viral load testing versus standard laboratory viral load testing (Appendix A). After randomization and during follow up, participants in both arms will continue to have routine clinic visits with clinical care, enhanced adherence counselling and blood draws from clinic staff as per South African and local guidelines, which recommend monthly visits until viral suppression is achieved. Repeat viral load testing will be performed at the clinician's discretion in accordance with South African guidelines, which recommend a repeat viral load after three months of enhanced adherence counselling. The Xpert® HIV-1 VL (or similar WHO approved assay e.g. m-PIMA HIV-1/2 VL) will be used in the point-of-care arm, and standard laboratory viral load testing in the standard of care arm. Clinic staff will be encouraged to use point-of-care viral load

For peer review only

results to provide immediate enhanced adherence counselling in the point-of-care arm, while in the SOC arm laboratory viral load results will be available to guide enhanced adherence counselling at the next clinic visit, which can be scheduled at the healthcare worker and client's discretion (typically after 28 days to coincide with next ART collection). Those with viral load ≥1000 copies/ml will meet criteria for viral failure (two consecutive viral loads ≥1000 copies/mL over 3 months apart), and will be managed according to South African viral failure guidelines, which recommend consideration of switching to a second-line ART regimen.[17] Those with viral loads <1000 copies/mL will continue their current first-line ART .[17] At the 24 week study exit visit a study outcome viral load will be measured using a laboratory reference assay (e.g. Alinity m HIV-1 or similar validated assay) for comparison between the two arms.

At enrolment, participants demographics, socioeconomic status and medical history will be recorded by research staff using standard questionnaires and review of clinical notes. During follow up, research staff will review participants clinic and laboratory notes to record time timing of receipt of viral load results, enhanced adherence counselling and switches to second-line ART. Data will be captured using electronic case report forms (CRFs) in REDCap.

During implementation of point-of-care viral load testing and after the study ends, a research assistant will perform in depth interviews and focus group discussions with clinic staff to explore implementation of point-of-care testing for management of viraemia (see section 12). Interviews and focus groups will be audio recorded, transcribed and analysed using Framework analysis.

6. STUDY SETTING

The study will take place at the Prince Cyril Zulu Clinic with support from the adjacent CAPRISA eThekwini Clinical Research Site. The Prince Cyril Zulu Clinic is a large public clinic situated next to the main transport hub in central Durban and provides HIV, tuberculosis sexual health and primary care services to a large, diverse, urban population. Prince Cyril Zulu Clinic sees approximately 40 people per month with viraemia ≥1000 copies/mL on first-line ART.

7. PARTICIPANT IDENTIFICATION AND RECRUITMENT

7.1. Study Participants

HIV-positive adults aged ≥18 years, receiving first-line ART, with latest viral load in the past 6 weeks ≥1000 copies/ml, and not having received enhanced adherence counselling.

7.2. Inclusion Criteria

The participant may enter the study if ALL of the following apply:

- Participant is willing and able to give informed consent for participation in the study.
- HIV positive adult, Male or Female, aged ≥18 years.
- Receiving first-line ART
- Latest viral load in the past 6 weeks ≥1000 copies/mL

For peer review only - http://bmjopen.bmj.com/site/about/guidelines.xhtml

7.3. Exclusion Criteria

The participant may not enter the study if ANY of the following apply:

- Has already received enhanced adherence counselling at a previous visit
- Requires hospital admission
- Pregnant

Pregnant women will not be enrolled in the study as they are routinely referred out of PCZ CDC for antenatal care. Participants who become pregnant during the study will be referred for antenatal care but will continue to be offered follow up in the study.

8. STUDY PROCEDURES

8.1. Recruitment

HIV-positive patients on first-line ART and with recent viraemia will be identified by clinic staff using National Health Laboratory Service (NHLS) 'High Viral Load Reports', electronic clinic information systems and clinic notes, and offered referral to the study team.

8.2. Informed Consent

A research assistant or research nurse/designee will approach patients at the clinic who have been identified as having a high viral load. The research assistant or research nurse/designee will describe the study, address any questions and ask for voluntary participation. If the participant is willing, they will be invited to a private room in the clinic where the research assistant or designee will present written and verbal versions of the Participant Information and Informed Consent detailing no less than: the exact nature of the study; what it will involve for the participant; the implications and constraints of the protocol; the known side effects and any risks involved in taking part. It will be clearly stated that the participant is free to withdraw from the study at any time for any reason without prejudice to future care, and with no obligation to give the reason for withdrawal.

The participant will be allowed as much time as they wish to consider the information, and the opportunity to question the Investigator or other independent parties to decide whether they will participate in the study. Written Informed Consent will then be obtained by means of participant dated signature and dated signature of the person who presented and obtained the Informed Consent. The person who obtained the consent must be suitably qualified and experienced, and have been authorised to do so by the Chief Investigator. A copy of the signed Informed Consent will be offered to the participant. The original signed form will be retained at the study site.

The participant must personally sign and date the latest approved version of the Informed Consent form before any study specific procedures are performed.

8.3. Screening and Eligibility Assessment

After informed consent has been obtained, a research nurse will review the study inclusion/exclusion criteria with the patient. Contact details, demographics, a medical history and vital signs will be recorded. The patients clinical and laboratory record will be reviewed. Pregnancy testing will be performed for all

For peer review only

women aged 18-55 who have not yet had a pregnancy test on that clinic visit. Patients deemed eligible for the study will be enrolled within 7 days of screening.

8.4. Randomisation, blinding and code-breaking

A CAPRISA statistician will generate the allocation sequence using random numbers generated in a statistical software program such as STATA 14.0 (Statacorp, Texas, USA) or SAS 9.4 (SAS Institute Inc., Cary, USA). Randomisation will be stratified by first-line ART regimen at enrolment (efavirenz-based or dolutegravir-based). The allocation sequence will contain variable block sizes, with participants randomised in a 1:1 ratio to the intervention or standard of care arm. The allocation sequence will be programmed into the REDCap (Vanderbilt University, Nashville, USA) enrolment electronic case report form (eCRF). All study staff apart from the statistician and data manager will be blinded to the allocation sequence. At enrolment, the research assistant or research nurse will complete the enrolment eCRF, which will automatically assign the intervention allocation and generate a unique participant identification number (PID). As this is an open label study, study staff, clinic staff and participants will not be blinded to intervention allocation.

8.5. Baseline Assessments

8.5.1. Baseline demographics and medical history

At enrolment, all participants will have a baseline sociodemographic questionnaire, clinical history including ART history, and examination. South African guidelines for clinical assessment and management of patients with a high viral load will be followed,[17] including enhanced adherence counselling performed by clinic staff using the pre-enrolment viral load result. In both arms, enhanced adherence counselling will follow South African guidelines, which include ART education, evaluation of social support, mental health screening and use of treatment supporters where appropriate.[18]

8.5.2. Laboratory testing

For all participants, a phlebotomist or nurse will draw venous blood for tests recommended by South African guidelines for investigation of viraemia, which currently include CD4 count testing, hepatitis B surface antigen, creatinine and haemoglobin. Venous blood for storage and retrospective HIV drug resistance testing will also be drawn.

8.6. Follow-up Routine Clinic Visits

Participants in both arms will have routine clinic visits scheduled at the healthcare worker and participants discretion, but normally every 28 days to coincide with ART collection. At these visits, clinic staff will provide ongoing enhanced adherence guidelines, clinical support and ART when necessary, as per the standard of care in South African National Guidelines. Clinicians will be able to request viral load testing as per South African guidelines, which recommend a repeat viral load 3 months after the first high result ≥1000 copies/mL.

8.6.1. Viral load testing at follow-up routine clinic visits

For participants randomised to the point-of-care arm, a phlebotomist or nurse will draw venous blood for point-of-care viral load testing in the clinic. Results should be available in approximately two hours. Staff will be encouraged to provide results in the same clinic visit to inform ongoing management. If results are

For peer review only

not available in the same visit, or if the client cannot wait, they will be available at the next clinic visit scheduled at staff and patient's discretion.

In the standard of care arm, a phlebotomist or nurse will draw venous blood for laboratory viral load testing which will be sent to the standard of care National Health Laboratory Service (NHLS) as per routine clinic procedures. Results are normally available after 7 days and will be provided to the participant at the next clinic visit, arranged at the participants and healthcare worker's discretion (typically after 7 -28 days for results, depending on the participants availability, ART supply and clinic schedules).

For participants in both arms, a venous blood sample will also be drawn for storage alongside the repeat viral load result.

8.6.2. Management of viral load results at follow-up routine clinic visits

Viral load results will be managed in accordance with South African Department of Health guidelines, which include guidance for management of both NNRTI and dolutegravir-based regimens (Appendix C).[17] Participants with a viral load ≥1000 copies/ml may meet criteria for viral failure (two consecutive viral loads ≥1000 copies/mL over 3 months apart) and will be considered for switch to second-line ART. Switch to second-line ART and the choice of regimen will be at the clinician's discretion and will depend on the participant's first-line ART, previous ART history, participant's preferences, perceived adherence and current South African national and local clinical guidelines (Appendices C and D).[17] The risks and benefits of potential new regimens will be discussed with the participant as per usual practice. Participants with a second viral load <1000 copies/mL will continue first-line ART, with enhanced adherence counselling for those with low level viraemia between 50-999 copies/mL (Table 1). If South Africa introduces dolutegravir during the study period, then study participants on NNRTI-based first-line ART with a suppressed viral load <50 copies/mL will be eligible for dolutegravir-based first-line ART.

Table 1: Management of viral load results during follow-up

HIV viral load result	Definition	Action
≥1000 copies/mL	Potential viral failure	Consider switch to second-line ART
50-999 copies/mL	Low level viraemia	Continue current first-line ART and enhanced adherence counselling
<50 copies/mL	Viral suppression	Continue first-line ART, if on NNRTI then may be eligible for dolutegravir based regimen.

8.6.3. Data captures at routine clinic visits

Research staff will monitor participants clinical records to capture visit dates, whether enhanced adherence counselling was performed, medication prescriptions including ART, and laboratory results.

8.7. Study Exit Visit

Clinical follow up will end at 24 weeks when a study outcome viral load will be measured using a laboratory reference assay (e.g. Alinity m HIV-1 or similar validated assay) for comparison between the two arms. A venous blood sample for storage and retrospective HIV drug resistance testing will also be taken. At 24 weeks, any participant who has not attended the study exit visit will be called by the research team and asked to attend. If by 30 weeks it has not been possible to contact the participant, they will be exited from the study.

For peer review only - http://bmjopen.bmj.com/site/about/guidelines.xhtml

8.8. Extended follow up of routine data

For participants who agree, research staff may access routine medical records, prescription data and laboratory results for up to five years after the study exit visit, in order to determine longer term retention in care, ART adherence and viral load results. For participants who are lost to follow up, and who provided consent at enrolment, the participants vital status may be checked on the South African National Population Register.

8.9. Sample Handling

8.9.1. Point-of-care testing

Point-of-care VL testing will be performed by a nurse, phlebotomist or technician in the clinic, who will have received comprehensive training on all aspects of performing point-of-care viral load testing and been assessed as competent by the CAPRISA laboratory site manager. All point-of-care viral load testing procedures will be performed in a separate, specially prepared room in the clinic, using a small centrifuge and the Xpert HIV-1 VL assay on the GeneXpert platform, or a similar WHO approved point-of-care assay (e.g. the m-PIMA HIV-1/2 VL). Testing will be performed following manufacturer instructions, using approximately 4 mLs of venous blood, drawn using aseptic technique. The sample will be centrifuged to separate approximately 1 mL of plasma which will be pipetted into the assay cartridge. The cartridge will be placed in the fully automated assay platform, with results available after approximately 90 minutes. In the case of an invalid result, a repeat blood sample may need to be drawn for point-of-care testing. If repeat point-of-care testing is not possible (e.g. machine failure) the repeat sample will be sent for standard laboratory testing. Error codes will be checked regularly and reported to the CAPRISA laboratory staff and manufacturer to identify and correct sample processing or machine errors.

8.9.2. Standard laboratory viral load testing

Laboratory viral load testing will be performed by a nurse or phlebotomist in the clinic according to standard clinic procedures. In brief, approximately 4 mLs of venous will be drawn using aseptic technique into an EDTA tube and transported on the same day to the National Health Laboratory Service for testing using their routine laboratory assays. Currently, the National Health Service performs viral load tests on the Alinity m HIV-1 assay (Abbott, Chicago, USA) at the Addington Hospital Laboratory, or the Inkosi Albert Luthuli Hospital Laboratory, depending on machine availability and capacity.

8.9.3. Other standard laboratory tests

At enrolment, approximately 20 mLs of venous blood will be drawn for CD4 count testing (4 mLs EDTA), hepatitis B surface antigen (5 mLs SST), creatinine (5 mLs SST) and haemoglobin (4 mLs EDTA). Samples will be transported on the same day to the National Health Laboratory Service for testing using their routine laboratory assays.

8.9.4. Storage samples and HIV drug resistance testing

Participants who provide additional consent will also have 10 mLs of venous blood for storage and retrospective ART drug level and HIV drug resistance testing, taken at enrolment, during follow up alongside the repeat viral load test, and at study exit. At enrolment, 5 mLs of urine will also be taken for storage and ART drug level testing. Samples will be stored in the CAPRISA biorepository according to Good Clinical Laboratory Practice. At the end of the study, HIV drug resistance testing will be performed on viraemic samples. The threshold of viraemia for HIV drug resistance testing will be determined by the likelihood of amplification on the assay being used. We will use standard Sanger sequencing or next-

For peer review only

generation sequencing with the Illumina MiSeq platform. Remaining blood will be stored for ten years after the end of the study and will be made available to the study team or other investigators who have obtained ethical approval for additional projects. After ten years, remaining stored samples will be disposed of.

8.9.5. Potential evaluation of newer point-of-care assays

If new point-of-care assays that may improve HIV care become available (such as viral load, HIV drug resistance, and ART drug level assays), we may use samples from this study to compare results from the new assay to the laboratory reference assay. At blood draw visits we may ask participants to provide up to 8 mLs venous blood and/or a finger-prick blood sample for evaluation of these new point-of-care assays. The new assay results would be for validation purposes only and would not be used to guide clinical management.

8.10. Discontinuation/Withdrawal of Participants from Study

Each participant has the right to withdraw from the study at any time. In addition, the Investigator may discontinue a participant from the study at any time if the Investigator considers it necessary for any reason including:

- Ineligibility (either arising during the study or retrospectively having been overlooked at screening)
- Significant protocol deviation
- Significant non-compliance with treatment regimen or study requirements
- Withdrawal of Consent

Participants who withdraw from the study will not be replaced. Data for participants who withdraw will be included in intention to treat analysis. The reason for withdrawal will be recorded in the Case Report Form.

8.11. Definition of End of Study

The end of study is the date 30 weeks after enrolment of the last participant.

9. SAFETY REPORTING

This study does not involve an investigational medicinal product or intervention that affects physiology, and therefore will not require serious adverse event reporting. However, for completeness we will report all cases of mortality in the study to the University of KwaZulu-Natal's Biomedical Research Ethics Committee (BREC).

10. STATISTICS AND ANALYSIS

10.1. Description of Statistical Methods

At the conclusion of the study, we will assess the proportion of participants achieving study outcomes in each arm, with 95% confidence intervals calculated using the Wilson method. We will use Fisher's exact test to compare the proportions achieving study outcome measures in each arm. However, as this is a

For peer review only

pilot study, we will not be powered to test the hypotheses that there is a difference in proportions between the two arms for most of our outcome measures.

10.2. Analysis of Outcome Measures

10.2.1. Primary outcome

We will calculate the proportion of participants in each arm achieving the binary primary outcome of viral suppression <50 copies/mL at 24 weeks (Table 2). Participants who are lost to follow up with no viral load result will be included the analysis as not having achieved the primary outcome, meaning all participants enrolled in each arm will be included in the analysis.

10.2.2. Secondary outcomes

We will calculate the proportion of patients with viraemia at the Prince Cyril Zulu clinic who are enrolled in the study. The denominator will be the number of patients with viraemia during the study enrolment period, which will be calculated by clinic staff using routinely generated NHLS high viral load reports. These reports are routinely used for monitoring and evaluation of clinic performance and include the number of first high viral loads > 1000 copies/mL taken from Prince Cyril Zulu each week. To assess study follow up, we will calculate the proportion of participants who attend the study exit visit at 24 weeks after enrolment. To assess implementation of point-of-care viral load testing, we will calculate the proportion of point-of-care viral load tests which are provided to participants on the same day, the proportion of viral loads ≥1000 copies/mL which resulted in same day enhanced adherence counselling, and the proportion of viral loads ≥1000 copies/mL resulting in appropriate same-day switch to second-line ART.

10.2.3. Tertiary Outcomes

We will assess the median number of days (and interquartile range) from enrolment to detection of viral failure, and from enrolment to appropriate switch to second-line ART, amongst participants with viral failure. We will also calculate the proportion of participants in each arm who are appropriately switched to a dolutegravir-based first or second line ART regimen by 24 weeks, and also the proportion of those with HIV drug resistance detected at 24 weeks after enrolment.

10.3. The Number of Participants

PCZC sees approximately 40 patients per month with viraemia on first-line ART. We conservatively estimate (based on our previous study) that we could recruit approximately 40% of these in 6 months, a total of 100 participants. However, as this is a feasibility study, the exact number that can be enrolled cannot be determined. The COVID-19 pandemic adds further uncertainty, as interruptions to care may decrease potential enrolment, or lead to higher numbers of people with viraemia during the study period, which could also increase enrolment. Therefore, while we anticipate 100 participants, the final number

Table 2: Study & process evaluation outcomes with precision estimates

Outcome	Estimated n/N, %	95% CI (Wilson)
Percentage with viral load <50 copies/ml at 24 weeks in POC arm	35/50, 70.0	56.2-80.9
Percentage with viral load <50 copies/ml at 24 weeks in SOC arm	25/50, 50.0	36.6-63.4
Percentage of eligible patients successfully enrolled in study	100/240*, 41.7	35.6-48.0
Percentage of those enrolled who are retained at 24 weeks	90/100, 90.0	82.6-94.5
Percentage in POC arm with same-day viral load testing	40/50, 79.5	70.0-88.8

*Assuming 240 eligible patients during enrolment period. POC = point-of-care, SOC = standard-of-care

For peer review only

may be between approximately 80-180 participants, and will be determined by the time and resources available. We will use NHLS, PCZC and study data to assess outcomes, with estimated precision, in Table 2. Although not the aim of this feasibility study, assuming 50% of SOC participants achieve the main outcome of viral load suppression <50 copies/ml at 24 weeks, a sample size of 50 participants per arm would provide 54% power to detect a +20% difference in the point-of-care testing arm, using a 2-sided alpha of 0.05. A difference of +10% would have a Newcombe-Wilson 95% confidence interval of -9 to +28%. A sample size of 90 participants per arm would provide 80% power to detect a +20% difference in the point-of-care testing arm, using a 2-sided alpha of 0.05. As all enrolled participants will be included in the final analysis, we have not adjusted for loss to follow up or potential withdrawals.

11. DATA MANAGEMENT

11.1. Access to Data

Direct access will be granted to authorised representatives from the University of Oxford and CAPRISA for monitoring and/or audit of the study to ensure compliance with regulations.

11.2. Data Handling and Record Keeping

The research team will capture study data using standardised electronic CRFs in REDCap. All data entry will undergo three stages of quality control including pre-programmed data validity checks in electronic CRFs, immediate source document review, and weekly quality reports generated by REDCap. The participants will be identified by a unique study specific number and/or code in any database. The name and any other identifying detail will NOT be included in any study data electronic file.

12. QUALITATIVE STUDY

12.1. Background

Point-of-care diagnostics have been used widely in healthcare systems in many low and middle income countries.[19] In HIV programmes, some tests such as rapid, lateral flow assays for diagnosis of HIV, have been evaluated, endorsed and incorporated into guidelines by the World Health Organization and successfully adopted in many settings.[20] However, other assays, such as more complex molecular polymerase chain reaction technologies for tuberculosis, have remained as laboratory tests despite being marketed as point-of-care assays.[19] A large systematic review of barriers to point-of-care HIV diagnostic implementation in low and middle income countries found that in 132 studies, integration of the point-of-care test into clinical work flows was the most commonly identified challenge to test utilisation.[21] However, there are few published implementation projects that have used a theory-based approach to assess implementation of point-of-care tests in healthcare systems, particularly in LMICs. One theory that has been widely used to assess implementation of new technologies in healthcare is Normalisation Process Theory. Normalisation Process Theory aims to identify what is needed to 'normalise' use of a technology in a healthcare system.

12.2. Aim

For peer review only

We aim to assess staff perspectives on implementation of point-of-care viral load testing for management of viraemia in a routine South African clinic.

12.3. Study design

During trial enrolment when implementation of point-of-care viral load testing is taking place, a trained research assistant with experience of qualitative research will perform the first round of semi-structured interviews with approximately 8-10 staff, and a focus group with approximately 8-10 staff. After completion of enrolment, we will perform a second round of interviews and focus group, to assess perspectives on implementation of point-of-care viral load testing and management of viraemia.

12.4. Participants

Approximately 10-15 staff involved in viral load testing and management of viraemia will be approached to participate in this qualitative study. Staff will include counsellors, phlebotomists, nurses, pharmacy staff, laboratory staff, doctors and health service managers. Focus groups and in-depth interviews may consist of the same or different staff members. However, we will endeavour to include the same staff members in the first and second round of interviews and focus groups.

12.5. Procedures

12.5.1. Informed consent

A trained research assistant will approach staff in the clinic, explain the nature of the project and offer participation. Should the staff member be interested, they will be taken to a private space and provided with written and verbal versions of the Qualitative Study Staff Participant Information Sheet and Informed Consent Form. The research assistant or other study team member will answer any questions that the staff member may have, and it will be clearly stated that staff are able to refuse to participate without adverse consequences for their working life. Once written informed consent has been provided, a copy will be offered to staff participant, and the original will remain with the research team.

12.5.2. Interviews and focus groups

Once the Informed Consent Form has been signed, the research assistant will use predetermined topic guides to conduct interviews and/or focus groups in a private room at the study clinic, or if necessary, at another suitable private venue of the participant’s choice. Interviews will last approximately 30-60 minutes, and focus groups 45-75 minutes. We will make an audio recording of interviews and focus group discussions. If a participant does not consent to audio recording, we will take a written recording of the interview or focus group.

12.5.3. Analysis

Audio and written recordings will be anonymised and translated from isiZulu (when necessary) and transcribed. Data will be analysed using thematic analysis as described by Attride-Stirling and reported accordingly.[22]

13. QUALITY CONTROL AND QUALITY ASSURANCE PROCEDURES

For peer review only

Study documentation including participant information sheets, informed consent forms, regulatory files, standard operating procedures, source documentation and electronic CRFs will be subject to internal quality audits by the CAPRISA Quality Assurance team, in accordance with CAPRISA standard operating procedures. CAPRISA routinely measure quality control and retention rates on all studies. The study will be conducted in accordance with relevant regulations and standard operating procedures.

14. ETHICAL AND REGULATORY CONSIDERATIONS

14.1. Declaration of Helsinki

The Investigator will ensure that this study is conducted in accordance with the principles of the Declaration of Helsinki.

14.2. Guidelines for Good Clinical Practice

The Investigator will ensure that this study is conducted in accordance with relevant regulations and with Good Clinical Practice.

14.3. Approvals

The protocol, participant information sheet and informed consent form, topic guides and any proposed advertising material will be submitted to OxTREC and BREC for written approval.

The Investigator will submit and, where necessary, obtain approval from the above parties for all amendments to the original approved documents.

14.4. Participant Confidentiality

The study staff will ensure that the participants' anonymity is maintained. The participants will be identified only by a participant ID number on all study documents and any electronic database. All documents will be stored in a secure cabinet in the locked research office, and will only accessible by study staff and authorised personnel. The electronic data will be stored in REDCap, which is a password protected data management service. All computers with access to the data will be password protected. The study will comply with the General Data Protection Regulation (GDPR), which requires that personal data must not be kept as identifiable data for longer than necessary for the purposes concerned.

For the qualitative evaluation of implementation processes, interviews and focus group discussions will take place in a private room. We will make a written record during the focus group or interview, or from the recorded audio, but we will not write down any information that might identify participants or others. For example, if the participant mentions names of colleagues, we will not write those names down. The recording and notes from interviews and focus group discussions will be stored in a locked cabinet and only the staff working on this study will be able to use them. We will destroy the recordings when the research is completed. Data will never be reported in a way that could potentially reveal a single participant's identity. If during interviews or focus groups a staff member reveals practice which is not in

For peer review only

line with professional standards and may cause harm to patients, we will discuss this with the staff member and the clinic manager.

14.5. Expenses and Benefits

Patient participants will be paid ZAR150 (approximately £8.00) after the enrolment visit and ZAR100 (approximately £5.50) after the 24 week visit for their participation in the research, in accordance with South African research guidelines.[23] Reasonable travel expenses for any visits additional to normal care will be reimbursed.

Staff participants will not be reimbursed as interviews or focus groups will take place during working hours, at a mutually convenient time that has been agreed with the clinic manager.

14.6. Reporting

The CI shall submit an Annual Progress Report to OxTREC and BREC on the anniversary of the date of approval of the study. In addition, the CI shall submit an End of Study Report to OxTREC and BREC within 12 months of completion of the study. If we extend follow up of routinely collected clinic and laboratory data, for up to five years after the last study exit visit, the study will remain under annual review by OxTREC and BREC while those data are being collected.

14.7. Other Ethical Considerations

All study participants will be HIV-positive and may face HIV-related stigma. Therefore, maintaining confidentiality is particularly important in this study (see section 14.4).

For staff interviews and focus groups, there is the possibility that they will feel unable to refuse participation, or that they will feel challenged at being interviewed about aspects of their work. While every effort will be made to maintain staff confidentiality, due to the small numbers of staff participating in the study it may be possible for superiors and colleagues to infer their identity indirectly. Therefore, the option to refuse participation without implications in their work will be made clear in the informed consent process. We will also warn staff about the risk of colleagues and superiors being able to infer their identity indirectly. Staff will not be asked questions which could threaten their work position or personal questions about their lives. Questions will focus work-related issues that are already likely to have been discussed in other settings, as the clinic team have implemented point-of-care viral load testing for management of viraemia.

15. FINANCE AND INSURANCE

15.1. Funding

This study is funded by a fellowship from Wellcome Trust PhD Programme for Primary Care Clinicians, the Internal Research England Global Challenges Research Fund (0007365) and the Africa Oxford Initiative.

For peer review only

15.2. Insurance

The University of Oxford has a specialist insurance policy in place which would operate in the event of any participant suffering harm as a result of their involvement in the research (Newline Underwriting Management Ltd, at Lloyd's of London).

16. PUBLICATION POLICY

Findings from this study will be presented at academic conferences and submitted to peer reviewed journals for Open Access publication in accordance with Wellcome Trust policies for Wellcome Trust funded research. Authorship and acknowledgements will follow the International Committee of Medical Journal Editors guidelines. Author accepted manuscripts will be posted on the investigators' institutional websites.

For peer review only

17. REFERENCES

1 Joint United Programme on HIV/AIDS (UNAIDS). Miles to Go: Global AIDS Update 2018. Geneva, 2018.

2 South African National Department of Health. National consolidated guidelines for the prevention of mother to child transmission of HIV (PMTCT) and the management of HIV in children, adolescents and adults. Pretoria, South Africa, 2015 http://www.sahivsoc.org/Files/ART Guidelines 15052015.pdf (accessed Feb 13, 2017).

3 World Health Organisation. Updated recommendations on first-line and second-line antiretroviral regimens and post-exposure prophylaxis and recommendations on early infant diagnosis of HIV: interim guidance. Geneva, 2018.

4 Murphy RA, Court R, Maartens G, Sunpath H. Second-line antiretroviral therapy in sub-Saharan Africa: It's time to mind the gaps. *AIDS Res Hum Retroviruses* 2017; **33**: 1181–4.

5 Labhardt ND, Ringera I, Lejone TI, *et al.* When patients fail UNAIDS' last 90 - the "failure cascade" beyond 90-90-90 in rural Lesotho, Southern Africa: a prospective cohort study. *J Int AIDS Soc* 2017; **20**: 1–10.

6 Lessells RJ. Virological monitoring – how it relates to adherence. In: 4th Southern African HIV Clinicians Society Conference. Johannesburg, 2018. http://www.sahivsoc2018.co.za/wp-content/uploads/2018/11/23B_Richard-Lessells-NV_VL-monitoring.pdf.

7 Jobanputra K, Parker LA, Azih C, *et al.* Impact and Programmatic Implications of Routine Viral Load Monitoring in Swaziland. *JAIDS J Acquir Immune Defic Syndr* 2014; **67**: 45–51.

8 Etoori D, Ciglenecki I, Ndlangamandla M, *et al.* Successes and challenges in optimizing the viral load cascade to improve antiretroviral therapy adherence and rationalize second-line switches in Swaziland. *J Int AIDS Soc* 2018; **21**: e25194.

9 Hermans LE, Tempelman HA, Carmona S, *et al.* High rates of viral resuppression on first-line ART after initial virological failure. In: Conference on Retroviruses and Opportunistic Infections. Boston, 2018. http://www.croiconference.org/sessions/high-rates-viral-resuppression-first-line-art-after-initial-virological-failure.

10 Shubber Z, Mills EJ, Nachega JB, *et al.* Patient-Reported Barriers to Adherence to Antiretroviral Therapy: A Systematic Review and Meta-Analysis. *PLOS Med* 2016; **13**: e1002183.

11 Dorward J, Drain PK, Garrett N. Point-of-care viral load testing and differentiated HIV care. *Lancet HIV* 2018; **5**: e8–9.

12 Bwana P, Ageng'o J, Danda J, Mbugua J, Handa A, Mwau M. Performance and Usability of mPIMATM HIV 1/2 Viral Load test in point of care settings in Kenya. *J Clin Virol* 2019; published online Oct. DOI:10.1016/j.jcv.2019.104202019.

13 Garrett NJ, Drain PK, Werner L, Samsunder N, Abdool Karim SS. Diagnostic Accuracy of the Point-of-Care Xpert HIV-1 Viral Load Assay in a South African HIV Clinic. *JAIDS J Acquir Immune Defic Syndr* 2016; **72**: e45–8.

14 Sacks JA, Fong Y, Gonzalez MP, *et al.* Performance of Cepheid Xpert HIV-1 viral load plasma assay to accurately detect treatment failure. *AIDS* 2019; **33**: 1881–9.

15 Drain PK, Dorward J, Violette L, *et al.* Point-of-care viral load testing improves HIV viral suppression and retention in care. In: Conference on Retroviruses and Opportunistic Infections.

For peer review only

Seattle, Washington, 2019.

16 Dorward J, Lessells R, Drain PK, *et al.* Dolutegravir for first-line antiretroviral therapy in low-income and middle-income countries: uncertainties and opportunities for implementation and research. *Lancet HIV* 2018; **5**: e400–4.

17 The South African National Department of Health. 2019 ART Clinical Guidelines for the management of HIV in Adults, Pregnancy, Adolescents, Children, Infants and Neonates. Pretoria, South Africa, 2019.

18 South African National Department of Health. Adherence Guidelines for HIV, TB and NCDs. Pretoria, 2016.

19 Jani I V., Peter TF. How Point-of-Care Testing Could Drive Innovation in Global Health. *N Engl J Med* 2013; **368**: 2319–24.

20 Drain PK, Rousseau C. Point-of-care diagnostics: Extending the laboratory network to reach the last mile. *Curr Opin HIV AIDS* 2017; **12**: 175–81.

21 Pai NP, Wilkinson S, Deli-Houssein R, *et al.* Barriers to Implementation of Rapid and Point-of-Care Tests for Human Immunodeficiency Virus Infection: Findings From a Systematic Review (1996-2014). *Point Care* 2015; **14**: 81–7.

22 Attride-Stirling J. Thematic networks: an analytic tool for qualitative research. *Qual Res* 2001; **1**: 385–405.

23 National Health Research Ethics Council (NHREC). Payment of trial participants in South Africa: Ethical considerations for Research Ethics Committees. South African Department of Health, 2012 http://www.nhrec.org.za/docs/Documents/Paymentconsiderations.pdf (accessed March 10, 2017).

For peer review only

18. APPENDIX A: STUDY FLOW CHART

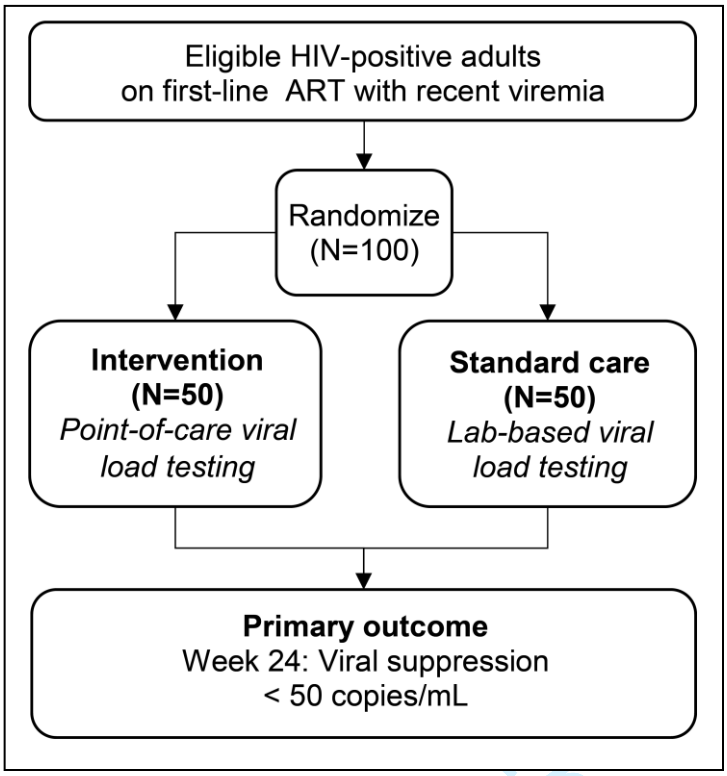

For peer review only

19. APPENDIX B: SCHEDULE OF STUDY PROCEDURES

	Enroll	Routine clinic visit follow-up[a]					Study exit
**Weeks in study**	**0**	**4**	**8**	**12**	**16**	**20**	**24**
Informed consent	X						
Locator information	X						
Demographics	X						
Medical history	X						
Vital signs	X	X	X	X	X	X	X
Physical examination	X						
Urine pregnancy test	X						
Eligibility screen	X						
Enhanced adherence counselling [b]	X	X	X	X	X[†]	X[†]	X[†]
Randomization	X						
Routine Department of Health testing [c]	X						
POC viral load (intervention arm)				X			
Laboratory viral load (standard of care arm)				X			
Reference viral load outcome measure							X
Evaluation of new POC assays[d]	X			X			X
Stored blood & HIV drug resistance testing[e]	X			X			X
Stored urine for ART drug level testing	X						

[a] Scheduled at participants and healthcare worker's discretion but typically every 28 days.
[b] Ongoing enhanced adherence counselling for participants with viral load ≥50 copies/mL.
[c] CD4 count testing (4 mLs EDTA), hepatitis B surface antigen (5 mLs SST), creatinine (5 mLs SST) and haemoglobin (4 mLs EDTA).
[d] Maximum of 8 mLs venous blood and/or a finger-prick capillary blood sample
[e§] Retrospective drug resistance testing for viraemic participants at enrolment, follow up or study exit (10 mLs EDTA)
ART = antiretroviral therapy, POC = point-of-care

For peer review only

20. APPENDIX C: SOUTH AFRICAN GUIDELINES FOR MANAGEMENT OF VIRAEMIA

Management of Viral Load Results in Infants, Children, Adolescents and Adults

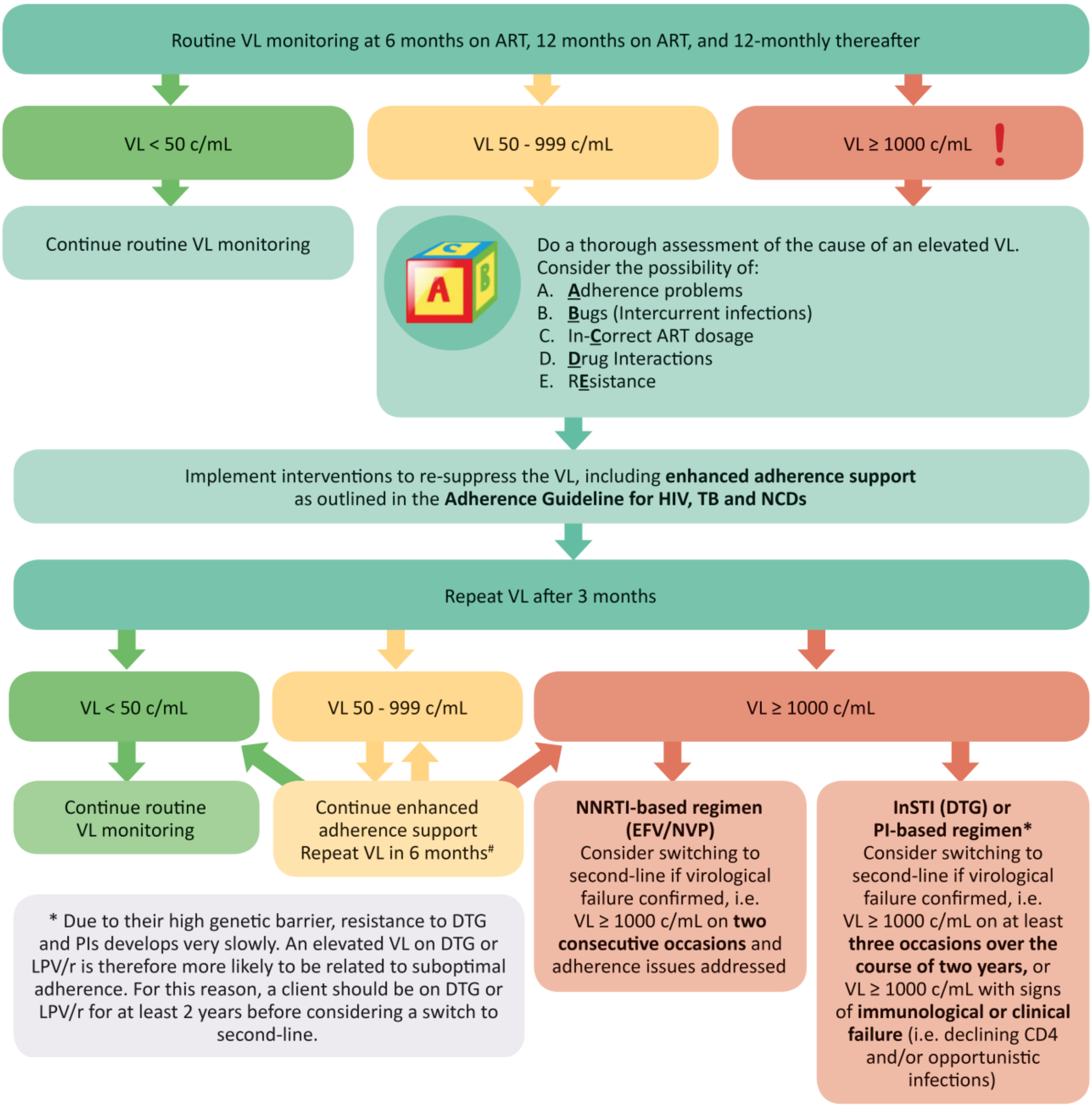

21. APPENDIX D: SOUTH AFRICAN GUIDELINES FOR CHOICE OF SECOND-LINE ART

Second-line ART Regimens for Adults with Confirmed Virological Failure

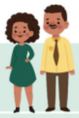

	First-Line Regimens			
	NNRTI-based Regimen		InSTI-based Regimen for > 2 years	
Regimen	TDF + 3TC/FTC + EFV/NVP		TDF + 3TC/FTC + DTG	
Resistance Testing	Resistance test not required		Resistance testing may be required under expert consultation [5]	
Resistance Test results	Not applicable		Not applicable	
HBV Co-infection Status [1]	HBV-negative	HBV-positive	HBV-negative	HBV-positive
New Regimen	AZT + 3TC/FTC + DTG[2]	TDF[1] + AZT + 3TC/FTC + DTG[2]	AZT + 3TC/FTC + LPV/r	TDF + 3TC/FTC + LPV/r [4]
	If DTG not suitable[2], AZT + 3TC/FTC + LPV/r	If DTG not suitable[2], TDF + 3TC + LPV/r[4]		

22. APPENDIX E: AMENDMENT HISTORY

Amendment No.	Protocol Version No.	Date issued	Author(s) of changes	Details of Changes made
1	1.1	10th November 2019	Jienchi Dorward	1. Addition of co-investigators Mrs H Ngobese and Dr Y Sookrajh<br>2. Addition of alternative, World Health Organization approved point-of-care viral load assays that could be used in the study<br>3. Addition of optional extended follow up of participants routinely collected clinic data and vital status in the South African National Population Register.<br>4. Change in the duration of blood storage from two to ten years<br>5. Addition of the potential evaluation of new point-of-care viral load assays<br>6. Minor clarifications regarding i) storage blood being drawn at week 12 visit, ii) tertiary objective c) changing from a proportion to time to event outcome iii) simplified description of procedures for invalid point-of-care results
2	2.0	24 June 2020	Jienchi Dorward	1. Broader range for sample size (80-180) to allow more flexibility due to COVID-19 in this feasibility study

peer review only

				2. Clarification that enhanced adherence counselling at a previous visit (rather than the current visit) is an exclusion.<br>3. Addition of randomisation stratified by first-line ART regimen at enrolment<br>4. Addition of stored urine samples at enrolment<br>5. Inclusion of validation of other point-of-care tests (including assays that use finger-prick capillary blood samples) that may improve HIV care, in addition to viral load assays.<br>6. Minor clarifications around i) the threshold of viraemia for retrospective HIV drug resistance testing and adherence counselling ii) the assays used for routine NHLS laboratory viral load testing iii) and the definition of the end of the study

For peer review only

SPIRIT
STANDARD PROTOCOL ITEMS: RECOMMENDATIONS FOR INTERVENTIONAL TRIALS

SPIRIT 2013 Checklist: Recommended items to address in a clinical trial protocol and related documents*

Section/item	Item No	Description	Addressed on page number
**Administrative information**			
Title	1	Descriptive title identifying the study design, population, interventions, and, if applicable, trial acronym	1
Trial registration	2a	Trial identifier and registry name. If not yet registered, name of intended registry	3 & 12
	2b	All items from the World Health Organization Trial Registration Data Set	3, 12, See PACTR
Protocol version	3	Date and version identifier	12
Funding	4	Sources and types of financial, material, and other support	16
Roles and responsibilities	5a	Names, affiliations, and roles of protocol contributors	1
	5b	Name and contact information for the trial sponsor	16
	5c	Role of study sponsor and funders, if any, in study design; collection, management, analysis, and interpretation of data; writing of the report; and the decision to submit the report for publication, including whether they will have ultimate authority over any of these activities	16
	5d	Composition, roles, and responsibilities of the coordinating centre, steering committee, endpoint adjudication committee, data management team, and other individuals or groups overseeing the trial, if applicable (see Item 21a for data monitoring committee)	NA

For peer review only

**Introduction**			
Background and rationale	6a	Description of research question and justification for undertaking the trial, including summary of relevant studies (published and unpublished) examining benefits and harms for each intervention	5
	6b	Explanation for choice of comparators	5
Objectives	7	Specific objectives or hypotheses	5 & 6
Trial design	8	Description of trial design including type of trial (eg, parallel group, crossover, factorial, single group), allocation ratio, and framework (eg, superiority, equivalence, noninferiority, exploratory)	6
**Methods: Participants, interventions, and outcomes**			
Study setting	9	Description of study settings (eg, community clinic, academic hospital) and list of countries where data will be collected. Reference to where list of study sites can be obtained	6
Eligibility criteria	10	Inclusion and exclusion criteria for participants. If applicable, eligibility criteria for study centres and individuals who will perform the interventions (eg, surgeons, psychotherapists)	6 & 7
Interventions	11a	Interventions for each group with sufficient detail to allow replication, including how and when they will be administered	8
	11b	Criteria for discontinuing or modifying allocated interventions for a given trial participant (eg, drug dose change in response to harms, participant request, or improving/worsening disease)	8
	11c	Strategies to improve adherence to intervention protocols, and any procedures for monitoring adherence (eg, drug tablet return, laboratory tests)	8
	11d	Relevant concomitant care and interventions that are permitted or prohibited during the trial	8
Outcomes	12	Primary, secondary, and other outcomes, including the specific measurement variable (eg, systolic blood pressure), analysis metric (eg, change from baseline, final value, time to event), method of aggregation (eg, median, proportion), and time point for each outcome. Explanation of the clinical relevance of chosen efficacy and harm outcomes is strongly recommended	9,10, Table 2
Participant timeline	13	Time schedule of enrolment, interventions (including any run-ins and washouts), assessments, and visits for participants. A schematic diagram is highly recommended (see Figure)	9, Table 1

For peer review only - http://bmjopen.bmj.com/site/about/guidelines.xhtml

Sample size	14	Estimated number of participants needed to achieve study objectives and how it was determined, including clinical and statistical assumptions supporting any sample size calculations	11
Recruitment	15	Strategies for achieving adequate participant enrolment to reach target sample size	7

Methods: Assignment of interventions (for controlled trials)

Allocation:

Sequence generation	16a	Method of generating the allocation sequence (eg, computer-generated random numbers), and list of any factors for stratification. To reduce predictability of a random sequence, details of any planned restriction (eg, blocking) should be provided in a separate document that is unavailable to those who enrol participants or assign interventions	7
Allocation concealment mechanism	16b	Mechanism of implementing the allocation sequence (eg, central telephone; sequentially numbered, opaque, sealed envelopes), describing any steps to conceal the sequence until interventions are assigned	7
Implementation	16c	Who will generate the allocation sequence, who will enrol participants, and who will assign participants to interventions	7
Blinding (masking)	17a	Who will be blinded after assignment to interventions (eg, trial participants, care providers, outcome assessors, data analysts), and how	7
	17b	If blinded, circumstances under which unblinding is permissible, and procedure for revealing a participant's allocated intervention during the trial	NA

Methods: Data collection, management, and analysis

Data collection methods	18a	Plans for assessment and collection of outcome, baseline, and other trial data, including any related processes to promote data quality (eg, duplicate measurements, training of assessors) and a description of study instruments (eg, questionnaires, laboratory tests) along with their reliability and validity, if known. Reference to where data collection forms can be found, if not in the protocol	10
	18b	Plans to promote participant retention and complete follow-up, including list of any outcome data to be collected for participants who discontinue or deviate from intervention protocols	10

Data management	19	Plans for data entry, coding, security, and storage, including any related processes to promote data quality (eg, double data entry; range checks for data values). Reference to where details of data management procedures can be found, if not in the protocol	10, 11
Statistical methods	20a	Statistical methods for analysing primary and secondary outcomes. Reference to where other details of the statistical analysis plan can be found, if not in the protocol	10, 11
	20b	Methods for any additional analyses (eg, subgroup and adjusted analyses)	10, 11
	20c	Definition of analysis population relating to protocol non-adherence (eg, as randomised analysis), and any statistical methods to handle missing data (eg, multiple imputation)	10, 11
**Methods: Monitoring**			
Data monitoring	21a	Composition of data monitoring committee (DMC); summary of its role and reporting structure; statement of whether it is independent from the sponsor and competing interests; and reference to where further details about its charter can be found, if not in the protocol. Alternatively, an explanation of why a DMC is not needed	NA
	21b	Description of any interim analyses and stopping guidelines, including who will have access to these interim results and make the final decision to terminate the trial	NA
Harms	22	Plans for collecting, assessing, reporting, and managing solicited and spontaneously reported adverse events and other unintended effects of trial interventions or trial conduct	NA
Auditing	23	Frequency and procedures for auditing trial conduct, if any, and whether the process will be independent from investigators and the sponsor	11
**Ethics and dissemination**			
Research ethics approval	24	Plans for seeking research ethics committee/institutional review board (REC/IRB) approval	12
Protocol amendments	25	Plans for communicating important protocol modifications (eg, changes to eligibility criteria, outcomes, analyses) to relevant parties (eg, investigators, REC/IRBs, trial participants, trial registries, journals, regulators)	12

For peer review only - http://bmjopen.bmj.com/site/about/guidelines.xhtml

Consent or assent	26a	Who will obtain informed consent or assent from potential trial participants or authorised surrogates, and how (see Item 32)	7
	26b	Additional consent provisions for collection and use of participant data and biological specimens in ancillary studies, if applicable	9
Confidentiality	27	How personal information about potential and enrolled participants will be collected, shared, and maintained in order to protect confidentiality before, during, and after the trial	10
Declaration of interests	28	Financial and other competing interests for principal investigators for the overall trial and each study site	16
Access to data	29	Statement of who will have access to the final trial dataset, and disclosure of contractual agreements that limit such access for investigators	16
Ancillary and post-trial care	30	Provisions, if any, for ancillary and post-trial care, and for compensation to those who suffer harm from trial participation	9
Dissemination policy	31a	Plans for investigators and sponsor to communicate trial results to participants, healthcare professionals, the public, and other relevant groups (eg, via publication, reporting in results databases, or other data sharing arrangements), including any publication restrictions	12
	31b	Authorship eligibility guidelines and any intended use of professional writers	12
	31c	Plans, if any, for granting public access to the full protocol, participant-level dataset, and statistical code	16
**Appendices**			
Informed consent materials	32	Model consent form and other related documentation given to participants and authorised surrogates	NA
Biological specimens	33	Plans for collection, laboratory evaluation, and storage of biological specimens for genetic or molecular analysis in the current trial and for future use in ancillary studies, if applicable	NA

*It is strongly recommended that this checklist be read in conjunction with the SPIRIT 2013 Explanation & Elaboration for important clarification on the items. Amendments to the protocol should be tracked and dated. The SPIRIT checklist is copyrighted by the SPIRIT Group under the Creative Commons "Attribution-NonCommercial-NoDerivs 3.0 Unported" license.

For peer review only - http://bmjopen.bmj.com/site/about/guidelines.xhtml
